# GWAS on longitudinal growth traits reveals different genetic factors influencing infant, child, and adult BMI

**DOI:** 10.1126/sciadv.aaw3095

**Published:** 2019-09-04

**Authors:** Alexessander Couto Alves, N. Maneka G. De Silva, Ville Karhunen, Ulla Sovio, Shikta Das, H. Rob Taal, Nicole M. Warrington, Alexandra M. Lewin, Marika Kaakinen, Diana L. Cousminer, Elisabeth Thiering, Nicholas J. Timpson, Tom A. Bond, Estelle Lowry, Christopher D. Brown, Xavier Estivill, Virpi Lindi, Jonathan P. Bradfield, Frank Geller, Doug Speed, Lachlan J. M. Coin, Marie Loh, Sheila J. Barton, Lawrence J. Beilin, Hans Bisgaard, Klaus Bønnelykke, Rohia Alili, Ida J. Hatoum, Katharina Schramm, Rufus Cartwright, Marie-Aline Charles, Vincenzo Salerno, Karine Clément, Annique A. J. Claringbould, BIOS Consortium, Cornelia M. van Duijn, Elena Moltchanova, Johan G. Eriksson, Cathy Elks, Bjarke Feenstra, Claudia Flexeder, Stephen Franks, Timothy M. Frayling, Rachel M. Freathy, Paul Elliott, Elisabeth Widén, Hakon Hakonarson, Andrew T. Hattersley, Alina Rodriguez, Marco Banterle, Joachim Heinrich, Barbara Heude, John W. Holloway, Albert Hofman, Elina Hyppönen, Hazel Inskip, Lee M. Kaplan, Asa K. Hedman, Esa Läärä, Holger Prokisch, Harald Grallert, Timo A. Lakka, Debbie A. Lawlor, Mads Melbye, Tarunveer S. Ahluwalia, Marcella Marinelli, Iona Y. Millwood, Lyle J. Palmer, Craig E. Pennell, John R. Perry, Susan M. Ring, Markku J. Savolainen, Fernando Rivadeneira, Marie Standl, Jordi Sunyer, Carla M. T. Tiesler, Andre G. Uitterlinden, William Schierding, Justin M. O’Sullivan, Inga Prokopenko, Karl-Heinz Herzig, George Davey Smith, Paul O'Reilly, Janine F. Felix, Jessica L. Buxton, Alexandra I. F. Blakemore, Ken K. Ong, Vincent W. V. Jaddoe, Struan F. A. Grant, Sylvain Sebert, Mark I. McCarthy, Marjo-Riitta Järvelin, Early Growth Genetics (EGG) Consortium

**Affiliations:** 1 Department of Epidemiology and Biostatistics, MRC-PHE Centre for Environment and Health, School of Public Health, Imperial College London, London, UK.; 2 School of Biosciences and Medicine, Faculty of Health and Medical Sciences, University of Surrey, Surrey, UK.; 3 Department of Obstetrics and Gynaecology, University of Cambridge, Cambridge, UK.; 4 NIHR Cambridge Biomedical Research Centre, Cambridge, UK.; 5 MRC Unit for Lifelong Health and Ageing at UCL, University College London, London, UK.; 6 The Generation R Study Group, Erasmus MC, University Medical Center Rotterdam, Rotterdam, Netherlands.; 7 Department of Paediatrics, Erasmus MC, Sophia Children’s Hospital, Rotterdam, Netherlands.; 8 Division of Obstetrics and Gynaecology, The University of Western Australia, Perth, Western Australia, Australia.; 9 The University of Queensland Diamantina Institute, The University of Queensland, Woolloongabba, Queensland, Australia.; 10 Department of Medical Statistics, London School of Hygiene and Tropical Medicine, London, UK.; 11 Department of Genomics of Common Disease, School of Public Health, Imperial College London, Hammersmith Hospital, London, UK.; 12 Centre for Pharmacology and Therapeutics, Division of Experimental Medicine, Department of Medicine, Imperial College London, Hammersmith Hospital, London, UK; 13 Department of Clinical and Experimental Medicine, School of Biosciences and Medicine, University of Surrey, Surrey, UK.; 14 Division of Human Genetics, The Children’s Hospital of Philadelphia, Philadelphia, PA, USA.; 15 Institute of Biomedicine, Department of Physiology, University of Eastern Finland, Kuopio, Finland.; 16 Institute for Molecular Medicine Finland, University of Helsinki, Helsinki, Finland.; 17 Institute of Epidemiology I, Helmholtz Zentrum München, German Research Center for Environmental Health, Munich Neuherberg, Germany.; 18 Division of Metabolic Diseases and Nutritional Medicine, Dr von Hauner Children’s Hospital, Ludwig-Maximilians University Munich, Munich, Germany.; 19 MRC Integrative Epidemiology Unit at the University of Bristol and NIHR Bristol Biomedical Research Center, Bristol, UK.; 20 Population Health Science, Bristol Medical School, University of Bristol, Bristol, UK.; 21 Center for Life Course Health Research, Faculty of Medicine, University of Oulu, Oulu, Finland.; 22 Department of Genetics and Institute for Biomedical Informatics, Perelman School of Medicine, University of Pennsylvania, Philadelphia, PA, USA.; 23 Genomics and Disease Group, Bioinformatics and Genomics Programme, Centre for Genomic Regulation (CRG), Barcelona, Catalonia, Spain.; 24 Pompeu Fabra University (UPF), Barcelona, Catalonia, Spain.; 25 Hospital del Mar Medical Research Institute (IMIM), Barcelona, Catalonia, Spain.; 26 Spanish Consortium for Research on Epidemiology and Public Health (CIBERESP), Madrid, Spain.; 27 Sidra Medical and Research Center, Doha, Qatar.; 28 Center for Applied Genomics, Abramson Research Center, The Children’s Hospital of Philadelphia, Philadelphia, PA, USA.; 29 Department of Epidemiology Research, Statens Serum Institut, Copenhagen, Denmark.; 30 Aarhus Institute of Advanced Studies (AIAS), Aarhus University, Aarhus, Denmark.; 31 UCL Genetics Institute, University College London, London, UK.; 32 Institute for Molecular Bioscience, University of Queensland, Brisbane, Queensland, Australia.; 33 Translational Laboratory in Genetic Medicine (TLGM), Agency for Science, Technology and Research (A*STAR) Singapore, Singapore.; 34 MRC Lifecourse Epidemiology Unit, University of Southampton, Southampton General Hospital, Southampton, UK.; 35 NIHR Southampton Biomedical Research Centre, University of Southampton and University Hospital Southampton NHS Foundation Trust, Southampton, UK.; 36 Medical School, Royal Perth Hospital, University of Western Australia, Perth, Western Australia, Australia.; 37 COPSAC, The Copenhagen Prospective Studies on Asthma in Childhood, Faculty of Health Sciences, University of Copenhagen, Copenhagen, Denmark.; 38 CRNH Ile de France, Hôpital Pitié-Salpêtrière, Paris, France.; 39 Obesity, Metabolism, and Nutrition Institute and Gastrointestinal Unit, Massachusetts General Hospital, Boston, MA, USA.; 40 Department of Medicine, Harvard Medical School, Boston, MA, USA.; 41 Institute of Human Genetics, Helmholtz Center Munich, German Research Center for Environmental Health, Neuherberg, Germany.; 42 Institute of Human Genetics, Technische Universität München, München, Germany.; 43 Institute for Reproductive and Developmental Biology, Imperial College London, London, UK.; 44 Inserm, UMR 1153 (CRESS), Paris Descartes University, Villejuif, Paris, France.; 45 University Medical Centre Groningen, Department of Genetics, Antonius Deusinglaan 1, 9713 AV Groningen, Netherlands.; 46 Department of Epidemiology, Erasmus MC, University Medical Center Rotterdam, Rotterdam, Netherlands.; 47 Department of Mathematics and Statistics, University of Canterbury, Christchurch, New Zealand.; 48 Department of General Practice and Primary Health Care, University of Helsinki, and Helsinki University Hospital, Helsinki, Finland.; 49 Department of Chronic Disease Prevention, National Institute for Health and Welfare, Helsinki, Finland.; 50 Folkhalsan Research Center, Helsinki, Finland.; 51 MRC Epidemiology Unit, University of Cambridge School of Clinical Medicine, Institute of Metabolic Science, Cambridge Biomedical Campus, Cambridge, UK.; 52 Institute of Biomedical and Clinical Science, University of Exeter Medical School, University of Exeter, Royal Devon and Exeter Hospital, Exeter, UK.; 53 National Institute for Health Research, Imperial College Biomedical Research Centre, London, UK.; 54 Health Data Research UK London, Imperial College London, London, UK.; 55 Department of Pediatrics, Perelman School of Medicine, University of Pennsylvania, Philadelphia, PA, USA.; 56 Institute of Diabetes, Obesity and Metabolism, Perelman School of Medicine, University of Pennsylvania, Philadelphia, PA, USA.; 57 School of Psychology, College of Social Science, University of Lincoln Brayford Pool Lincoln, Lincolnshire, UK.; 58 Human Genetics and Medical Genomics, Faculty of Medicine, University of Southampton, Southampton, UK.; 59 South Australian Health and Medical Research Institute, Adelaide, South Australia, Australia.; 60 Great Ormond Street Hospital Institute of Child Health, University College London, London, UK.; 61 Australian Centre for Precision Health, University of South Australia Cancer Research Institute, North Terrace, Adelaide, South Australia, Australia.; 62 NIHR Southampton Biomedical Research Centre, University of Southampton and University Hospital Southampton NHS Foundation Trust, Southampton, UK.; 63 Wellcome Centre for Human Genetics, University of Oxford, Oxford, UK.; 64 Cardiovascular Medicine Unit, Department of Medicine, Karolinska Institute, Stockholm, Sweden.; 65 Research Unit of Mathematical Sciences, University of Oulu, Oulu, Finland.; 66 Research Unit of Molecular Epidemiology, Helmholtz Zentrum München, German Research Center for Environmental Health, Neuherberg, Germany.; 67 German Center for Diabetes Research (DZD), Neuherberg, Germany.; 68 Kuopio Research Institute of Exercise Medicine, Kuopio, Finland.; 69 Department of Clinical Physiology and Nuclear Medicine, Kuopio University Hospital, Kuopio, Finland.; 70 Department of Clinical Medicine, University of Copenhagen, Copenhagen, Denmark.; 71 Department of Medicine, Stanford University Medical School, Stanford, CA, USA.; 72 ISGlobal, Centre for Research in Environmental Epidemiology (CREAL), Barcelona, Spain.; 73 Clinical Trial Service Unit and Epidemiological Studies Unit (CTSU), University of Oxford, Old Road Campus, Oxford, UK.; 74 Medical Research Council Population Health Research Unit (MRC PHRU) at the University of Oxford, Oxford, UK.; 75 School of Public Health and Robinson Research Institute, University of Adelaide, Adelaide, Australia.; 76 Avon Longitudinal Study of Parents and Children, School of Social and Community Medicine, University of Bristol, Bristol, UK.; 77 Division of Internal Medicine, and Biocenter of Oulu, Faculty of Medicine, Oulu University, Oulu, Finland.; 78 Department of Internal Medicine, Erasmus MC, University Medical Center Rotterdam, Rotterdam, Netherlands.; 79 Liggins Institute, University of Auckland, Auckland, New Zealand.; 80 A Better Start—National Science, Challenge, University of Auckland, Auckland, New Zealand.; 81 Oxford Centre for Diabetes, Endocrinology and Metabolism, University of Oxford, Churchill Hospital, Headington, Oxford, UK.; 82 Biocenter Oulu, University of Oulu, Oulu, Finland.; 83 Research Unit of Biomedicine, University Oulu, Oulu, Finland.; 84 Medical Research Center and Oulu University Hospital, University of Oulu, Oulu, Finland.; 85 Department of Gastroenterology and Metabolism, Poznan University of Medical Sciences, Poznan, Poland.; 86 MRC Social, Genetic and Developmental Psychiatry Centre, Institute of Psychiatry, King’s College London, De Crespigny Park, London, UK.; 87 School of Life Sciences, Pharmacy and Chemistry, Kingston University, Kingston upon Thames, UK.; 88 Department of Life Sciences, College of Health and Life Sciences, Brunel University London, London, UK.; 89 Section of Investigative Medicine, Division of Diabetes, Endocrinology and Metabolism, Imperial College London, London, UK.; 90 Oxford NIHR Biomedical Research Centre, Oxford University Hospitals NHS Foundation Trust, John Radcliffe Hospital, Oxford, UK.; 91 Unit of Primary Care, Oulu University Hospital, Oulu, Finland.

## Abstract

Early childhood growth patterns are associated with adult health, yet the genetic factors and the developmental stages involved are not fully understood. Here, we combine genome-wide association studies with modeling of longitudinal growth traits to study the genetics of infant and child growth, followed by functional, pathway, genetic correlation, risk score, and colocalization analyses to determine how developmental timings, molecular pathways, and genetic determinants of these traits overlap with those of adult health. We found a robust overlap between the genetics of child and adult body mass index (BMI), with variants associated with adult BMI acting as early as 4 to 6 years old. However, we demonstrated a completely distinct genetic makeup for peak BMI during infancy, influenced by variation at the *LEPR/LEPROT* locus. These findings suggest that different genetic factors control infant and child BMI. In light of the obesity epidemic, these findings are important to inform the timing and targets of prevention strategies.

## Teaser

Longitudinal data find a new variant controlling BMI in infancy and reveal genetic differences between infant and adult BMI.

## INTRODUCTION

Childhood obesity and its relation to later adult health, social inequality, and psychosocial well-being remain one of the most important unsolved health concerns of the 21st century (*1*). Epidemiological studies have revealed unambiguous associations between alterations of childhood body mass index (BMI) trajectory and risk of adult obesity and multimorbidities, including type 2 diabetes (*2*) and other cardiometabolic diseases (*3*). From a life-course perspective, genetic and environmental factors driving child growth may have a lasting influence on maintaining health (*4*). Within this framework, identification of the genetic determinants of the critical periods in child development is important for understanding the mechanisms underpinning adult health and preventing disease development.

To date, we have gained considerable insights into the shared genetic makeup of childhood and adult BMI (*5*, *6*). These previous studies were designed to identify genetic variants associated with BMI and obesity acting through the ages of 2 to 18 years. However, BMI does not remain constant, or follow a linear pattern throughout life, particularly not from birth until the age of adiposity rebound (AR) (*7*, *8*). On the contrary, the BMI trajectory in healthy individuals (fig. S1) encompasses three periods characterized by (i) a rapid increase in BMI up to the age of 9 months [adiposity peak (AP)], (ii) a rapid decline in BMI up to the age of 5 to 6 years [adiposity rebound timepoint (AR)], followed by (iii) a steady increase until early adulthood, when BMI growth rate decelerates. We have yet to determine whether changes in timing, velocity, or amplitude of this trajectory, during infancy and childhood, are influenced by specific genetic factors, acting at different developmental stages. The identification of genetic determinants of early growth traits is a fundamental step toward understanding the etiology of obesity and could be important in informing future strategies to prevent and treat it.

The present study set out to model sex-specific individual postnatal growth velocity and BMI curves in children using high-density longitudinal data collected from primary health care or clinical research visits. We first conducted a genome-wide association study (GWAS) on six harmonized early growth traits: peak height velocity (PHV), peak weight velocity (PWV), age at AP (Age-AP), BMI at AP (BMI-AP), age at AR (Age-AR), and BMI at AR (BMI-AR). We then analyzed the GWAS summary statistics for these six early growth traits to gain insights into the genes and molecular pathways involved and to assess the overlap between the genetic etiology of early growth traits and adult phenotypes. In particular, we tracked the changes in the genetic determinants of BMI occurring throughout infancy, later childhood, and adulthood.

## RESULTS

**Fig. 1:**
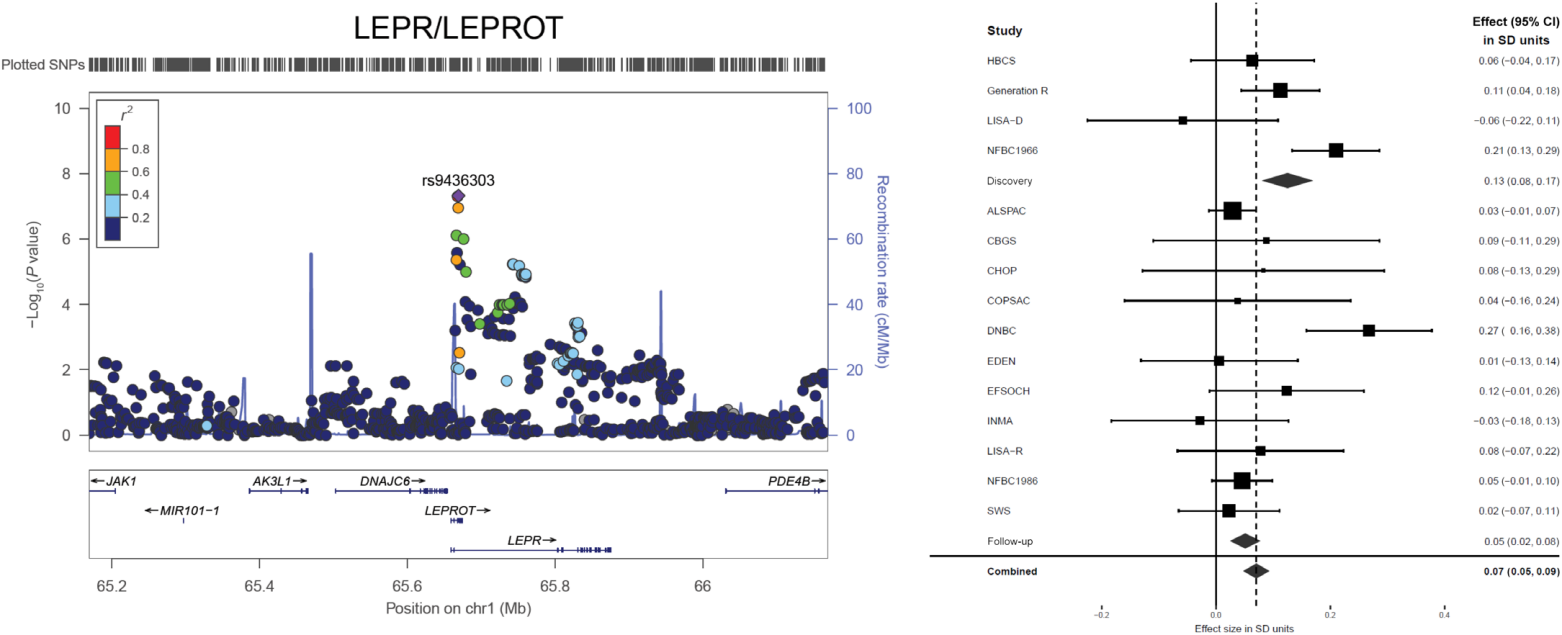
Regional association and forest plot of the novel genome-wide significant locus associated with BMI-AP. Purple diamond indicates the most significantly associated SNP in stage 1 meta-analysis, and circles represent the other SNPs in the region, with coloring from blue to red corresponding to *r*^2^ values from 0 to 1 with the index SNP. The SNP position refers to the National Center for Biotechnology Information (NCBI) build 36. Estimated recombination rates are from HapMap build 36. Forest plots from the meta-analysis for each of the identified loci are plotted abreast. Effect size [95% confidence interval (CI)] in each individual study, discovery, follow-up, and combined meta-analysis stages is presented from fixed-effects models (heterogeneity of the SNP rs9436303 in *LEPR/LEPROT*; see fig. S6). At this locus, there was heterogeneity between the studies in discovery (*I*^2^ = 72.1%, *P* = 0.01) and combined stage (*I^2^* = 59.3%, *P* = 0.002) fixed-effects meta-analyses that was mainly due to LISA-D, EDEN, and the larger well-defined NFBC1966 study (fig. S6, A and D). Removing the studies that showed inflated results from meta-analyses did not change the point estimates (fig. S6, C, F, and G). Both fixed- and random-effects models gave very similar results (fig. S6, B and E).

We conducted two-stage meta-analyses of GWASs on six early growth traits: PHV (in centimeters per month), PWV (in kilograms per month), Age-AP (in years), BMI-AP (in kilograms per square meter), Age-AR (in years), and BMI-AR (in kilograms per square meter). Figure S2 summarizes the study design, while participant characteristics, genotyping arrays, imputation and quality control for the discovery, and follow-up studies are presented in tables S1 and S2 and fig. S3. In the discovery stage (stage 1), we meta-analyzed GWAS from four population-based studies comprising between 6051 and 7215 term-born children of European ancestry that had both genetic and early growth trait data (stage 1; see Methods, table S1, and fig. S4). From the stage 1 inverse variance meta-analyses, we selected a total of eight loci with either *P* < 1 × 10^−7^ or *P* < 1 × 10^−5^ in/near genes known to be associated with obesity and metabolic traits in published GWAS or candidate gene studies. Table S3 shows selection criteria, false discovery rate (FDR), and bias-reduced effect size estimates for the selected single-nucleotide polymorphisms (SNPs). In stage 2 meta-analysis, we followed up these results in up to 16,550 term-born children from up to 11 additional studies (stage 2; see Methods and table S2). In the combined stage 1 + 2 meta-analysis of the discovery and follow-up studies (including up to 22,769 children), we identified a common variant in each of the four independent loci, associated at *P* < 5 × 10^−8^ with one or more of the early growth traits (Table 1, Fig. 1, and fig. S5).

**Table 1:** Summary statistics of the eight independent SNPs associated with PWV in infancy, BMI-AP in infancy, Age-AR, and BMI-AR in discovery (stage 1) and follow-up (stage 2) and in combined meta-analyses.

					**Stage 1 (*n* = 7,215)**		**Stage 2 (*n* = 16,550)**		**Combined (*n* = 22,769)**	
**Index SNP**	**Chromosome** position^*^	**In/near** **gene**	**Effect allele/** **other allele**	**Effect allele** **frequency**	**Effect** **size (SE)**	***P***	**Effect size** **(SE)**	***P***	**Effect** **size (SE)**	***P***
PWV (kg/month)^†^										
rs2860323	chr2:614210	*TMEM18*	G/A	0.12	0.09 (0.02)	5.9 × 10^−5^	0.02 (0.02)	4.7 × 10^−1^	0.06 (0.02)	3.9 × 10^−4^
BMI-AP (kg/m2)^†^										
rs9436303	chr1:65430991	*LEPR/LEPROT*	G/A	0.22	0.13 (0.02)	**4.7 × 10**^**−8**^	0.05 (0.01)	6.7 × 10^−4^	0.07 (0.01)	**8.3 × 10**^**−9**^
rs10515235	chr5:96323352	*PCSK1*	A/G	0.21	0.09 (0.02)	9.7 × 10^−7^	0.03 (0.01)	1.5 × 10^−2^	0.05 (0.01)	2.4 × 10^−6^
Age-AR (years)^†^										
rs1421085	chr16:53767042	*FTO*	C/T	0.25	−0.10 (0.02)	**6.1 × 10**^**−8**^	−0.13 (0.01)	**7.1 × 10**^**−24**^	−0.12 (0.01)	**3.1 × 10**^**−30**^
rs2956578	chr5:36497552	Intergenic region^‡^	G/A	0.31	0.11 (0.02)	**6.7 × 10**^**−8**^	0.00 (0.01)	8.3 × 10^−1^	0.04 (0.01)	1.1 × 10^−3^
rs2817419	chr6:50845193	*TFAP2B*	A/G	0.76	−0.10 (0.02)	2.9 × 10^−6^	−0.07 (0.01)	1.8 × 10^−6^	−0.08 (0.01)	**4.4 × 10**^**−11**^
BMI-AR (kg/m2)^†^										
rs10938397	chr4:45180510	*GNPDA2*	G/A	0.35	0.09 (0.02)	5.4 × 10^−6^	0.05 (0.01)	3.1 × 10^−4^	0.06 (0.01)	**2.9 × 10**^**−8**^
rs2055816	chr11:85406487	*DLG2*	C/T	0.25	−0.13 (0.02)	1.4 × 10^−7^	−0.03 (0.02)	1.8 × 10^−1^	−0.07 (0.02)	5.1 × 10^−6^

*SNP positions are according to dbSNP build 147.

†The effect size is the change in SDs per effect allele from linear regression, adjusted for child’s sex and principal components (PCs) assuming an additive genetic model. BMI-AP was additionally adjusted for gestational age (GA). PWV, BMI-AP, and BMI-AR were log-transformed because of skewness in their distribution. Original phenotype measurement units are denoted in parentheses. None of the loci for PHV passed the selection criteria for stage 2 follow-up. *P* values for discovery and combined analysis are shown in bold if genome-wide significant (*P* < 5 × 10^−8^). The maximum sample size used in meta-analyses of each stage is shown in parentheses. Results are from inverse-variance fixed-effects meta-analysis of European ancestry children. The effect allele for each SNP is labeled on the positive strand according to HapMap.

‡Intergenic region between *RANBP3L* and *SLC1A3.*

### AR SNPs associate with adult BMI

Three of the four SNPs were associated with Age-AR and BMI-AR. These three variants were previously associated (*P* < 5 × 10^−8^) with adult BMI and adult weight in the literature (table S4) and in the UK Biobank PheWAS (phenome-wide association study) (*9*) (table S5), as well as with several adiposity-related phenotypes in PhenoScanner (*10*) (see Methods). The lead SNPs at each of these three loci were the following: rs1421085 at the locus harboring *FTO* (encoding a 2-oxoglutarate–dependent demethylase) and rs2817419 at the locus harboring *TFAP2B (*encoding transcription factor AP-2β) associated with Age-AR, and rs10938397 near *GNPDA2* (encoding adiposity regulating glucosamine-6-phosphate deaminase) locus associated with BMI-AR (Table 1 and fig. S5). Each lead SNP (rs1421085, rs2817419, and rs10938397) associated with Age-AR and BMI-AR explains approximately 0.2% of variance in the relevant early growth trait (see Methods).

### A new variant in *LEPR/LEPROT* associated with BMI-AP

The BMI-AP–associated SNP rs9436303 (Fig. 1 and Table 1) at the locus harboring *LEPR/LEPROT* (encoding the leptin receptor and the leptin receptor overlapping transcript) is novel. This novel variant is robustly associated with BMI-AP after applying a conservative bias-reducing correction for winner’s curse and a multiple testing correction for six phenotypes (α′ = 10^−8^; see Methods and table S3). The risk allele (G) of this variant increases both BMI-AP and adult plasma soluble leptin receptor levels (*P* = 1.19 × 10^−9^) (table S4) (*11*). The *LEPR/LEPROT* locus is in a chromosomal region, 1p31.3, that harbors another independent signal [ rs11208659: minor allele frequency (MAF) = 0.06; distance = 82.6 kilo–base pairs; *R*^2^ = 0.01] associated with early-onset obesity (*12*), but our SNP rs9436303 is associated with BMI-AP independently of this variant [linkage disequilibrium (LD) *R*^2^ = 0.01 and see conditional analysis in table S6]. There was some effect heterogeneity between studies for this variant (fig. S6, A and D), but excluding the two studies with inflated estimates eliminated heterogeneity (*I*^2^ = 0) in the stage 1 + 2 meta-analysis (fig. S6, C and F) without a substantial impact on effect sizes or significance levels. This SNP explains 0.3% of variance in BMI-AP (see Methods).

The SNP rs9436303 overlaps a regulatory region in a *LEPR* intron and is downstream from a processed transcript of *LEPROT* gene (table S7). *LEPROT* and *LEPR* overlap and share the same promoter but encode distinct transcripts with specific biological functions (*13*). The known biological function and molecular mechanism of the proteins encoded by the nearest genes in the four loci discovered are given in table S8. However, as with most GWAS-identified loci, the expression of these genes may not necessarily be influenced by the underlying causal variant/s tagged by the GWAS SNP, so we sought further evidence that the BMI-AP–associated variants influence expression in the following section.

### Cis colocalization of GWAS and expression quantitative trait locus signals

**Fig. 2:**
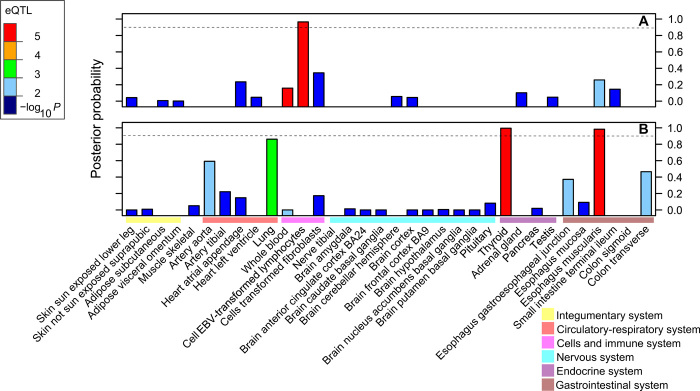
Tissue-specific posterior probabilities (PPs) of colocalization for *LEPR* and *LEPROT*. PP of eQTL and GWAS SNP sharing a causal variant regulating the gene expression levels of (A) *LEPR* and (B) *LEPROT*. Colocalization reported for GTEX eQTLs data in 34 tissues that express at least one of the genes. Bar plot color-coded according to the –log_10_ *P* value eQTL direct lookup in the corresponding GTEx tissue of the GWAS SNP. *LEPR* and *LEPROT* eQTLs colocalized with BMI-AP variant rs9436303.

To identify GWAS and expression quantitative trait loci (eQTLs) signals that share the same causal variants, we performed Bayesian colocalization analyses (*14*) using our stage 1 GWAS meta-analysis summary statistics and eQTL data from 44 postmortem tissues generated by the Genotype-Tissue Expression (GTEx) consortium (see Methods) (*15*). The lead GWAS variants with high (>95%) posterior probability (PP) of colocalization were followed-up in five separate studies (see Methods) using cis-eQTL data from five ex vivo tissues and combined with genomic annotation data (tables S9 and S10). In these analyses, we found high PPs of colocalization with local causal variants (>95%) driving the expression of *LEPR* and *LEPROT* (Table 2 and fig. S7). The colocalization results for each gene are markedly tissue specific (Fig. 2 and fig. S8). In ex vivo samples, the *LEPR/LEPROT* variant was in high LD with the top eQTLs of *LEPR* and *LEPROT* genes in omental fat, subcutaneous fat, and whole blood (table S9). Direct lookup of *LEPR/LEPROT* variant in eQTL data indicated that the G allele of this variant that raised BMI-AP in our GWAS up-regulated the NM017526 transcript of *LEPROT* and down-regulated the AK023598 transcript from the same gene in adult tissues (table S10). This observation was consistent across two different eQTL studies and four tissues, suggesting the involvement of alternative splicing of a cassette exon. The *LEPR/LEPROT* variant overlapped DNA binding motifs of transcription factors and regulatory regions, as well as enhancer and promoter histone marks in multiple tissues (fig. S9). In Avon Longitudinal Study of Parents and Children (ALSPAC), the same *LEPR/LEPROT* variant was associated with higher DNA methylation levels of a *LEPR* intron measured in blood samples taken from mother and offspring. In particular, associations were found during mother’s pregnancy and in offspring’s adolescence, but not at offspring’s birth, at childhood, or in mother’s middle age (table S11) (*16*). This observation might be consistent with the regulation of a constitutively expressed transcript, which is also supported by evidence that lower *LEPR* intron DNA methylation levels were associated with higher serum leptin concentrations (*17*). Together, these results suggest that shared causal variants in these loci regulate BMI trajectory at AP, orchestrate changes in gene expression in different tissues, and modulate methylation of the nearest genes during mother’s pregnancy and at specific developmental stages of the offspring.

**Table 2:** GWAS loci colocalized with eQTL in postmortem tissues from the GTEx data. Colocalization results refer to GWAS and eQTL SNP. PP, posterior probability

**Chr**	**Nearest gene**	**Trait**	**GWAS SNP**	**GWAS SNP** ***P* value**	**Tissue**	**eQTL SNP**	**eQTL** ***P* value**	**eQTL gene**	**Top eQTL** SNP*(*R*^2^)	**Colocalization** PP (%)**
1	*LEPR/LEPROT*	BMI-AP	rs9436303	8.3 × 10^−9^	Thyroid	rs9436301	7.9 × 10^−7^	*LEPROT*	rs9436745 (0.78)	99
					Esophagus muscularis	rs1887285	1.6 × 10^−6^	*LEPROT*	rs9436745 (0.78)	98
					Cell EBV- transformed lymphocytes	rs1887285	1.2 × 10^−7^	*LEPR*	rs77848204 (0.22)	96
6	*TFAP2B*	Age-AR	rs2817419	4.4 × 10^−11^	Testis	rs2635727	2.9 × 10^−7^	*TFAP2B*	rs2635727 (0.91)	99
					Sun-exposed skin lower leg	rs2635727	4.2 × 10^−6^	*TFAP2B*	rs2635727 (0.91)	98

*R^2^ values between GWAS SNP and GTEx top eQTL SNP for each gene (eGene) are shown for reference. Only results with a ** posterior probability (PP) of a shared causal variant of >95% are reported.

### Genetic determinants of adult BMI overlap with those determining AR but not AP

**Fig. 3:**
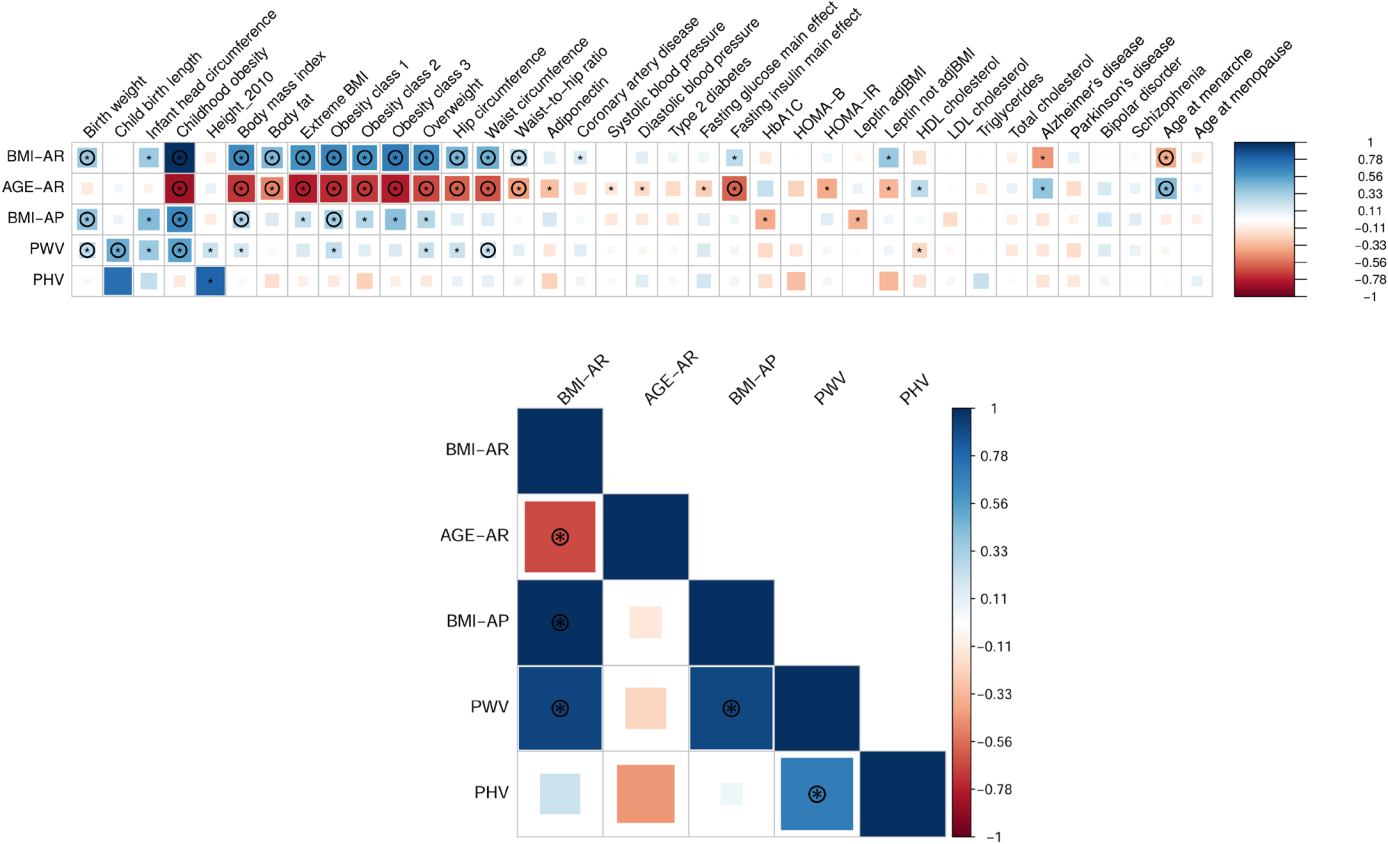
Genetic correlations between five early growth traits and a subset of 37 phenotypes. Only a selected list of 37 phenotypes is represented on the correlation matrix. Genetic correlation results for all 72 phenotypes are given in table S16. Blue, positive genetic correlation; red, negative genetic correlation. The correlation matrix underneath represents the genetic correlation among the five early growth traits themselves. The size of the colored squares is proportional to the *P* value, where larger squares represent a smaller *P* value. Genetic correlations that are different from 0 at *P* < 0.05 are marked with an asterisk. The genetic correlations that are different from 0 at an FDR of 1% are marked with a circle. Genetic correlations estimated with stage 1 meta-analysis GWAS summary statistics from the current and literature studies using LD score regression.

In our study, Age-AR and BMI-AR have moderate to very strong genetic correlations with adult BMI and other adult adiposity-related phenotypes, but BMI-AP does not (see Methods, Fig. 3, and table S12). Age-AR and BMI-AR had genetic correlations with multiple (more than four) adult complex phenotypes, including adult waist circumference (Age-AR *r*_g_ = −0.62; BMI-AR *r*_g_ = 0.48) and adult body fat percentage (Age-AR *r*_g_ = −0.49; BMI-AR *r*_g_ = 0.44). Adult BMI and adult obesity had strong genetic correlations with BMI-AR (*r*_g_ = 0.64 and *r*_g_ = 0.66) and Age-AR (*r*_g_ = −0.72 and *r*_g_ = −0.75) but weak correlation with BMI-AP (*r*_g_ = 0.29 and *r*_g_ = 0.33). The traits with genetic and phenotypic correlations that were directionally consistent (note S1**)** are reported in table S13. Genetic correlations of Age-AP with other traits could not be quantified because of low mean χ^2^ of the GWAS summary statistics. In summary, genetic correlation analyses suggest that the genetic factors influencing adult BMI, body fat percentage, waist circumference, and obesity are also associated with BMI-AR and Age-AR, but their overlap with BMI-AP is either absent or weak.

### Genetic risk score for adult BMI is associated with Age-AR and BMI-AR but not with Age-AP and BMI-AP

**Fig. 4:**
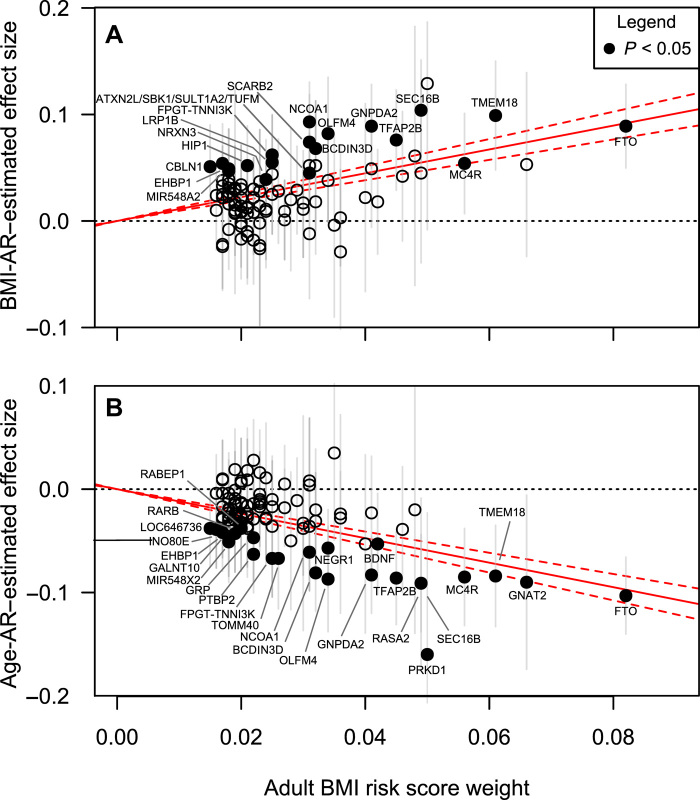
Adult BMI GRS analysis of early growth traits. Scatter plots show the effect size estimates (SD units) of the 97 adult BMI-associated SNP in GIANT consortium in the *x* axis and the corresponding effect size estimates (SD units) of the looked-up SNP of stage 1 meta-analysis GWAS on (**A**) BMI-AR and (**B**) Age-AR in the *y* axis. The effect size of the adult BMI increasing allele is plotted. The solid red line is the estimated effect of the GRS on the early growth phenotype, taking into account the uncertainty of the point estimates. The dashed line is the 95% CI of the predicted effect. Stage 1 meta-analysis GWAS SNPs with *P* < 0.05 are plotted with a solid circle and labeled with the nearest gene name. The scatter plots of the other early growth phenotypes are given in fig. S10.

To gain further insight into the observed genetic correlations with adult BMI and to understand the developmental timing of the adult BMI-associated variants, we constructed an adult BMI genetic risk score (GRS) based on the 97 adult BMI SNPs identified by the Genetic Investigation of Anthropometric Traits (GIANT) consortium (*18*) (Fig. 4 and table S14) and applied it to the six early growth traits (see Methods). The adult BMI variants and the GRS were consistently and robustly associated with Age-AR (*h*^2^_grs_ = 0.035, *P* = 2.6 × 10^−48^) and BMI-AR (*h*^2^_grs_ = 0.030, *P* = 1.7 × 10^−41^) but not with other early growth traits (Fig. 4 and table S15). In the remaining four early growth traits, the GRS explained a negligible proportion of variance (*h*^2^_grs_ < 0.001), and the adult BMI variants had inconsistent genetic effects (fig. S10 and table S15). In particular, the adult BMI variant effects on BMI-AP and PWV were highly heterogeneous (*P*_het_ < 2 × 10^−4^), with evidence of horizontal pleiotropy (MR-PRESSO; *P* < 2 × 10^−4^). This suggests that, in contrast with their effects on Age-AR and BMI-AR, the top loci associated with adult BMI do not have robust associations with the remaining four early growth traits. Thus, the underlying genetic determinants of adult BMI might differ from those influencing BMI-AP. Together, these data indicate that many GWAS variants associated with adult BMI have effects that begin in later childhood (4 to 6 years), as early as the Age-AR but not as early as AP (around 9 months).

### Gene set analyses suggest little overlap between pathways and networks controlling AP and AR

To combine information on the effects of common variants in biological pathways and networks underlying early growth, we applied a gene set enrichment analysis [Meta-Analysis Gene-set Enrichment of variaNT Associations (MAGENTA)] (*19*) to the discovery stage GWAS results (see Methods). We identified enrichment of gene sets (tables S16 and S17) but did not find evidence for overlap of enriched pathways and networks among early growth traits. Age-AR–associated regions are involved in the insulin-like growth factor 1 (IGF-1) signaling pathway (FDR < 0.05). The IGF-1 signaling pathway has a well-established role both in growth and in the regulation of energy metabolism through the activation of phosphatidylinositol 3-kinase (PI3K)/AKT pathway via either the insulin or the IGF-1 receptors (*20*).

### SNP heritability of Age-AR and BMI-AR is larger than BMI-AP

We estimated the chip SNP heritability (the proportion of variance explained by common SNPs) for the six early growth traits using LD score regression (LDSC) (see Methods). The heritability estimates for BMI-AR (*h*^2^_snp_ = 0.38), Age-AR (*h*^2^_snp_ = 0.36), PWV (*h*^2^_snp_ = 0.32), and BMI-AP (*h*^2^_snp_ = 0.29) were statistically significant (*P* < 0.05; Table 3). LDSC and SumHer (*21*) SNP heritability estimates (table S18) ranked these phenotypic heritabilities in a similar manner. The BMI-AP and BMI-AR estimates compared well with LDSC estimates for adult BMI (*h*^2^_snp_ = 0.27) in a much larger sample of the UK Biobank (*N* = 152,736). Twin and family study heritability estimates for BMI-AP (*h*^2^ = 0.75 to 0.78) (*22*, *23*) and BMI-AR (*h*^2^ = 0.4 to 0.6) (*24*, *25*) were higher than the SNP heritability estimated here. However, the ratio of the SNP heritability obtained from LDSC and the total heritability obtained from family and twin studies suggests that a considerable (39 to 95%; see Methods) proportion of BMI heritability can be attributed to common variants. As the LDSC heritability estimates of BMI-AP, BMI-AR, and adult BMI are comparable, the differences in the genetic etiology observed in our study cannot be trivially attributed to large disparities in the variance explained by genetic factors. Hence, together, these data suggest that distinct, heritable developmental processes control the BMI trajectory at AP and AR.

**Table 3:** SNP heritability of the early growth traits. SNP heritability estimated with LD score using all common SNPs (MAF > 0.01) in stage 1 GWAS meta-analysis.

**Trait**	**Estimated** **heritability**	**SE**	**95% CI**		**Mean χ^2^**	***P***
BMI-AP	0.29	0.08	0.13	0.46	1.03	4.7 × 10^−4^
BMI-AR	0.38	0.08	0.22	0.53	1.013	2.7 × 10^−6^
Age-AP	−0.03	0.08	−0.18	0.13	1.001	7.4 × 10^−1^
Age-AR	0.36	0.08	0.20	0.52	1.007	1.1 × 10^−5^
PHV	0.11	0.07	−0.03	0.25	1.006	1.3 × 10^−1^
PWV	0.32	0.07	0.18	0.45	1.011	2.5 × 10^−6^

## DISCUSSION

There are few reports of studies investigating the genetic bases of these well-established growth and BMI trajectories (*26*, *27*), and to our knowledge, our study is the largest genome-wide meta-analyses of early growth traits so far. In the present study, we identified four variants at four independent loci associated with three early growth traits, determined by modeling growth trajectories using high-density longitudinal data for height and weight. Our study provides insights into the developmental timings at which the genetic makeup of early and later measures of BMI overlaps or differs, and contributes to understanding the mechanisms and molecular pathways of early growth patterns.

The three common variants at *FTO*, *TFAP2B*, and *GNPDA2,* associated with timing of adiposity rebound and/or BMI-AR, are robustly associated with adult BMI and other adiposity traits. In contrast, the newly discovered variant at the *LEPR/LEPROT* locus associated with BMI-AP did not associate with other growth traits reported here, or in previous studies on childhood/adult BMI and obesity. This may indicate that genetic variants involved in adult BMI only start influencing BMI after AP and are robustly associated with child BMI by the time of AR. This is further corroborated by two additional lines of evidence provided by our study: (i) We observed strong genetic correlations of adult BMI, body fat percentage, and waist circumference with Age-AR and BMI-AR but not with Age-AP and BMI-AP, and (ii) the GRS constructed using adult BMI variants was robustly associated with Age-AR and BMI-AR but not with Age-AP and BMI-AP.

The difference in the genetic determinants of BMI-AP and BMI-AR and onward may be attributed to three factors: (i) BMI explains a relatively small proportion of body fat percentage (*R*^2^ < 0.3) in infancy (0 months < age ≤ 7 months) (*28*) but increasingly larger proportions (0.36 < *R*^2^ ≤ 0.8) in childhood (2 years ≤ age < 18 years) (*29*, *30*) and adulthood (*R*^2^ ≈ 0.8; age, >18 years) (*31*); (ii) the genes involved in the regulation of BMI during infancy seem to differ from those acting in later childhood onward, which suggests distinct biological processes acting throughout these developmental stages; and (iii) sustained changes in the infant environment after weaning and onward may progressively unmask the effects of adult BMI variants. Consistent with this view, there is some evidence that infants’ and children’s environment modifies the effect of genetic factors. In particular, having been breastfed modifies the strength of association of the *FTO* variant with BMI (*32*) and with BMI growth trajectories (*27*). On the other hand, the adult risk alleles of the *FTO* and *MC4R* variants are not associated with increased infant BMI (*26*), but *FTO’s* strength of association with BMI progressively increases in later childhood (4 to 11 years) (*24*). Likewise, BMI heritability increases throughout childhood up to young adulthood (4 to 19 years) (*22*, *24*, *25*), as offspring BMI starts resembling adult BMI as an anthropometric marker of adiposity, and as the shared environment between adults and offspring progressively increases. Consistently, BMI heritability increased between AP and AR, and a considerable proportion of heritability was explained by common variants in our study. The increase in BMI heritability with age might be explained by correlations between genotype and environment. Small genetic differences are magnified as children progressively select, modify, and create environments correlated with their genetic propensities, which, in turn, unmask the effects of other genetic variants in a feedforward loop. These processes gradually may increase the phenotype variance explained by genetic factors and thereby increase BMI heritability. All in all, our study supports the accrual of shared genetic determinants between later childhood and adult BMI (*5*, *6*), but not with infant BMI.

In our study, the IGF-1 pathway that links diet with growth was enriched for variants associated with Age-AR, but not Age-AP, in the MAGENTA analysis. Higher IGF-1 levels, via genetic and/or nutritional factors, can reduce growth hormone (GH) levels via a negative feedback (*33*). Subsequent lower circulating levels of GH can suppress lipolysis and contribute to fat accumulation (*34*), changing BMI trajectories and Age-AR, and, thereby, increasing risk of obesity and metabolic disorders. The regulation of the GH/IGF-1 axis is modulated by leptin and adiponectin levels, two hormones regulated by *LEPR/LEPROT* and *TFAP2B* genes, respectively (*35*).

The variant at *LEPR/LEPROT* colocalized with causal variants regulating the expression of *LEPR* and *LEPROT* in different tissues. *LEPROT* and the *LEPR* genes share the same promoter but encode distinct transcripts (*13*). *LEPROT* is cotranscribed with the *LEPR*, and both are expressed in multiple tissues with different functionalities. LEPR is widely distributed in peripheral tissues, shows signaling capability, and is thought to transport leptin across the blood-brain barrier (*25*). Some LEPR isoforms may function in leptin clearance or buffering (soluble LEPR). In our eQTL data, the G allele that raises BMI-AP up-regulates the NM017526 transcript of *LEPROT* but down-regulates AK023598 transcript from the same gene in adult tissues. This observation was consistent across the different eQTL studies and tissues, suggesting that this variant may regulate the alternative splicing of a cassette exon in adult blood and subcutaneous and omental adipose tissue. In addition, the *LEPR/LEPROT* variant was associated with methylation levels in the *LEPR* intron during mother’s pregnancy and at specific developmental stages of the offspring. Together, this functional analysis suggests that distinct molecular mechanisms in different tissues are involved in the expression regulation of these genes at different developmental stages.

*LEPROT* and the *LEPR* downstream mechanisms involved on the regulation of BMI are likely to be developmental stage dependent. In humans, loss-of-function mutations in the *LEPR* markedly increase weight of infants after birth that persists through adulthood (*36*). However, the regulatory elements of *LEPROT* and *LEPR* tagged by our GWAS SNP are not associated with BMI or any measure of adiposity in adults or in later childhood, despite being associated with BMI in infancy and involved in the control of the circulating levels of the soluble LEPR in adults. Hence, the regulatory variant identified is involved in the regulation of adult LEPR through a mechanism that does not alter BMI after later childhood (age, >4 years). More work is necessary to identify the impact of *LEPROT* mutations in weight gain and growth, as well as in the identification of the tissues and regulatory elements of the different LEPR isoforms.

Our study has limitations that should be taken into consideration when interpreting the data. First, dense longitudinal growth and GWAS data are only available in a few population studies worldwide, so we had limited power to detect genetic variants with smaller effects and/or low allele frequencies. Nevertheless, a post hoc power analysis showed that we are well powered to detect the reported effect sizes in the discovery sample (β = 0.065 SD units; power, 80%; significance level *P* < 5 × 10^−8^; see Methods). As a sex-stratified analysis would have halved the sample size, the analysis of sex-specific effects was left outside the scope of the paper. As in every joint meta-analysis GWAS, the final estimates may have suffered from winner’s curse (*37*). In our study, the follow-up sample is twice the size of the discovery sample. Consequently, the final joint analysis estimates are very close to the follow-up estimates and are thus potentially less biased. Second, it is noteworthy that these derived growth traits are likely to be influenced by a degree of measurement error and some heterogeneity, as some studies have fewer repeated measures around the time points being estimated. Ideally, regression would be weighted by the inverse variance of the phenotypes derived from the growth models. However, the variances for the derived outcomes could not be directly estimated because we used a model with random effects. The fact that we did not use inverse-weighted regression will increase SEs and decrease the power to detect associations. Despite this, we were still able to find genetic variants showing robust associations with these derived growth traits. Third, as the current approach implemented in MAGENTA focus on a fixed cutoff (the 95% percentile of the *P* value), our analysis has possibly missed enriched gene sets. Nevertheless, the top 10 gene sets that did not reach significance (FDR, >0.05) were reported. Last, we did not identify any variants associated with PHV, PWV, and Age-AP at genome-wide levels of significance, and this may be due to a combination of smaller genetic effects on growth at this stage of development, due to reduced statistical power because of smaller sample size, or because environmental factors masked the genetic influences at this age. The interplay between genetic variants, infant feeding, and other environmental factors also warrants additional research (*27*).

**Fig. 5:**
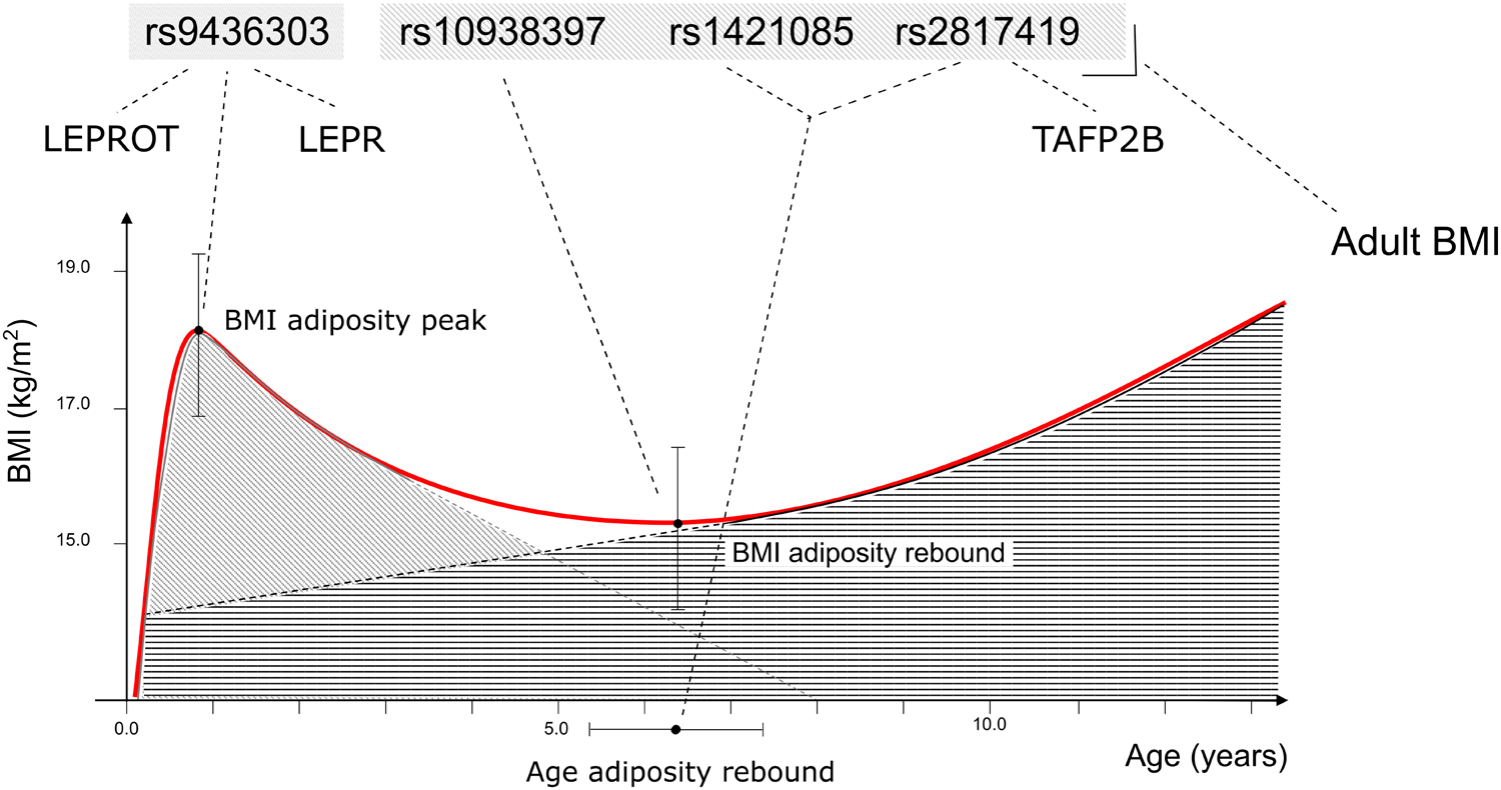
Proposed model of child BMI suggesting the superimposition of two biological phenomena under the genetic control of different loci. The schematic diagram shows the four genome-wide significant loci associated with early childhood growth traits and highlights the fact that only SNPs associated with phenotypes ascertained at AR are associated with adult BMI. The red curve represents the mean BMI trajectory from birth to puberty in the NFBC1966 cohort.

In conclusion, this longitudinal GWAS study, based on derived traits from growth modeling, has uncovered a completely new variant in *LEPR/LEPROT* locus that specifically associates with BMI at the peak of adiposity in infancy. The present study identified two BMI developmental stages in infancy and later childhood with distinct genetic makeup. Our results support the notion that genetic determinants of adult BMI progressively start acting in later childhood but not necessarily before the AP in infancy (*5*, *6*). This finding may corroborate a model of BMI development consisting of the superimposition of two biological processes with distinct genetic drivers (Fig. 5), which, in turn, suggests that interventions in childhood aiming to modify BMI and achieve long-lasting reductions in the risk of adult obesity need to take into account the developmental stage. We believe that the identification of genetic factors underpinning the BMI trajectory is a fundamental step toward understanding the etiology of obesity and may inform strategies to prevent and treat it.

## METHODS

### Longitudinal growth modeling and derivation of early growth traits

Early growth traits were derived from sex-specific individual growth curves using mixed-effects models of height, weight, and BMI measurements from birth to 13 years (fig. S1). All height and weight data were collected prospectively via either self-reported data or clinical measurements (tables S1 and S2). These traits were derived separately in each cohort (note S2).

#### Derivation of PHV and PWV

The methods for growth modeling and derivation of growth parameters from the fitted curves are described in detail in a previous publication (*38*). Parametric Reed1 growth model was fitted in sex-stratified nonlinear random-effect model as described previously (*39*). Term-born singletons (defined as ≥37 completed weeks of gestation) with at least three height or weight measurements from birth to 24 months of age were included in the Reed1 model fitting. Maximum-likelihood method for best fitting curves for each individual was used to estimate the growth parameter, PHV (in centimeters per month), and PWV (in kilograms per month).

#### Derivation of Age-AP, Age-AR, BMI-AP, and BMI-AR

The methods used for growth modeling of age and BMI have been previously described in detail by Sovio *et al.* (*26*). Because of the specificity of longitudinal changes in BMI, i.e., succession of peak and nadir as described in fig. S1, the data were divided into two age windows for modeling: (i) growth in infancy using height and weight data from 2 weeks to 18 months of age and (ii) growth in childhood using growth and weight data from 18 months to 13 years of age. Each cohort contributed most data available within any of these two age windows. In studies where the data available consisted of both height and weight data within a given window, the data point nearest to the mid time points of that window was used as a proxy for the BMI measurement. Before model fitting, age was centered using the median age of the relevant age window. For example, in the infant growth model at 0 to 1.5 years, the median age was 0.75 years (which was close to the average Age-AP), and in the childhood growth model at >1.5 to 13 years, the median age was 7.25 years (on average shortly after AR). Linear mixed-effects models were then fitted for log-transformed BMI. We used sex and its interaction with age as covariates, with random effects for intercepts (i.e., baseline BMI) and linear slope (i.e., linear change in BMI) over time. In addition to linear age effect, quadratic and cubic terms for age were included in the fixed effects to account for nonlinearity of BMI change over time.

#### Growth in infancy

The following model was used to calculate the Age-AP and BMI-AP, and the analysis was restricted to singletons with BMI measures from 2 weeks to 18 months of age. The model is as follows

log(BMI) = β_0_ + β_1_ Age + β_2_ Age^2^ + β_3_ Age^3^ + β_4_ Sex + *u*_0_ + *u*_1_ (Age) + ε

where BMI is expressed in kilograms per square meter and age in years. β_0_, β_1_, β_2_, β_3_, and β_4_ are the fixed-effects terms, *u*_0_ and *u*_1_ are the individual-level random effects, and ε is the residual error. The Age-AP was calculated from the model as the age at maximum BMI between 0.25 and 1.25 years according to preliminary research (*38*).

#### Growth in childhood

The model used to measure the age and BMI-AR in childhood is as follows

log(BMI) = β_0_ + β_1_ Age + β_2_ Age^2^ + β_3_ Age^3^ + β_4_ Sex +β_5_ Age × Sex + β_6_ Age^2^ × Sex + *u*_0_ + *u*_1_ (Age) + ε

where BMI is expressed in kilograms per square meter and age in years. β_0_, β_1_, β_2_, β_3_, β_4_, β_5_, and β_6_ are the fixed-effects terms, *u*_0_ and *u*_1_ are the individual-level random effects, and ε is the residual error. Age-AR was calculated as the age at minimum BMI between 2.5 and 8.5 years according to preliminary research (*38*).

### Stage 1 GWASs, genotyping, and imputation

Stage 1 genome-wide association analyses included up to 7215 children of European descent from five studies (four studies for each early growth trait) that had growth data and genome-wide data. These included the Helsinki Birth Cohort Study (Finland), Northern Finland Birth Cohort 1966 (NFBC1966; Finland), Lifestyle-Immune System–Allergy Study (LISA; Germany), The Western Australian Pregnancy Cohort Study (Raine, Australia), and Generation R (The Netherlands) (figs. S2 and S3). Informed consent was obtained from all study participants (or parental consent, as appropriate), and the local ethics committees as appropriate approved all study protocols. Study characteristics, genotyping platform, imputation and association test software used, as well as sample and genotyping and imputation quality control steps in each stage 1 study are given in table S1. Stage 1 consisted of a GWAS based on ~2.5 million directly genotyped or imputed SNPs. Imputation of nongenotyped SNPs was undertaken either with MACH or with IMPUTE and were imputed to HapMap phase 2 CEU reference panel after excluding genotyped SNPs with a MAF of <1%, call rate of at least ≥95%, and a Hardy-Weinberg equilibrium (HWE) *P* value cutoff as given in table S1.

### Stage 1 genome-wide association analyses and meta-analyses

According to the availability of dense enough data for growth modeling, a total of up to 7215, 6222, 6219, and 6051 children were used to analyze PHV/PWV, Age-AP, BMI-AP, and Age-AR/BMI-AR, respectively (fig. S2). We only included children who were born between 37 and 41 completed weeks of gestation (i.e., term born) from singleton pregnancies and children who had more than three growth measurements available within the age range in question. Gestational age (GA) was either defined from the date of the last menstrual period or ultrasound scans depending on the study. All six early growth traits except for Age-AP and Age-AR were naturally log-transformed to reduce skewness, and all traits were converted to *z*-scores before association testing to facilitate the comparison of results across the studies. We tested the directly genotyped and imputed variants for association with each of the six early growth traits in a linear regression model, assuming an additive genetic effect. The regression models were adjusted for sex and principal components (PCs) derived from the genome-wide data to adjust for potential population substructure (the necessary number of PCs included varied by study). GA is a marker of multiple factors influencing pregnancy that may influence the child growth trajectory. Regression of all phenotypes on GA adjusting for sex produced significant associations, apart from BMI-AR and Age-AR, which showed significant associations with sex only. On the basis of this observation, we adjusted all GWAS analyses for GA and sex apart from BMI-AR and Age-AR, which were adjusted for sex only. The risk of introducing collider bias was dismissed because gestational effects occur before birth, and the loci found did not overlap with GA signals in the GWAS catalog or PhenoScanner. The genome-wide association analyses (i.e., stage 1) were performed using either SNPTEST or MACH2QTL in each cohort, and data exchange facilities were provided by the AIMS server (*40*). All stage 1 study beta estimates and their SEs were meta-analyzed using the inverse-variance fixed-effects method in the METAL software (*41*). SNPs with poor imputation quality (e.g., *r*^2^ < 0.3 for MACH and “proper_info” score < 0.4 for IMPUTE) and/or an HWE *P* < 1 × 10^−4^ were excluded before the meta-analyses. Double genomic control (*42*) was applied: first, to adjust the statistics generated within each cohort and, second, to adjust the overall meta-analysis statistics. Results are reported as a change in SD units per risk allele as reported in Table 1.

### Selection of SNPs for stage 2 follow-up

All loci reaching *P* < 1 × 10^−7^ from stage 1 GWAS of each early growth trait were selected for follow-up in stage 2. These included the two SNPs associated with Age-AR in the *FTO* locus (rs1421085) and in the intergenic region between *RANBP3L* and *SLC1A3* (rs2956578), and the SNP associated with BMI-AP in *LEPR/LEPROT* (rs9436303). Four further SNPs [one SNP associated with BMI-AP near *PCSK1* (rs10515235), one SNP associated with Age-AR in *TFAP2B* (rs2817419), and two SNPs associated with BMI-AR near *GNPDA2* (rs10938397) and in *DLG2* (rs2055816)] were selected for follow-up on the basis of showing an association with an early growth trait at *P* < 1 × 10^−5^ and being in/near genes with established links to adiposity and metabolic phenotypes except for *DLG2*, a possible candidate gene involved in glucose metabolism (*43*). In addition, one locus with a plausible association (*P* = 5.91 × 10^−5^) with PWV, near *TMEM18* (rs2860323), was also selected for follow-up based on previous reports showing an association with severe early-onset obesity (*12*) and its association with BMI in adulthood (*44*) and childhood (*6*) (table S3). No loci for PHV or Age-AP passed the *P* value threshold or other selection criteria used for follow-up. Table S3 shows the SNP selection criteria and proxies used in more detail.

### Stage 2 follow-up of lead SNPs

For follow-up of lead signals selected from stage 1, we used data from up to 16,550 children of European descent from 12 additional population-based studies (up to 11 studies for each early growth trait), namely, the ALSPAC (United Kingdom), Cambridge Baby Growth Study (United Kingdom), Children’s Hospital of Philadelphia (United States), Copenhagen Prospective Study on Children (Denmark), Danish National Birth Cohort (Denmark), Étude des Déterminants pré- et postnatals du développement et de la santé de l’ENfant (EDEN; France), The Exeter Family Study of Childhood Health (United Kingdom), INfancia y Medio Ambiente Project (Spain), Lifestyle-Immune System–Allergy Study [LISA (R), Germany], Northern Finland Birth Cohort Study 1986 (Finland), The Physical Activity and Nutrition in Children (Finland), and Southampton Women’s Survey (United Kingdom). We used de novo SNP genotyped or imputed data for the eight SNPs (or proxies of *r*^2^ > 0.8) selected from stage 1 and tested their association in a total of 5367, 16,550, 12,256, and 12,192 children of European ancestry with PWV, BMI-AP, Age-AR, and BMI-AR, respectively (Fig. 2). Direct genotyping was performed in some follow-up studies by KBiosciences Ltd. (Hoddesdon, United Kingdom) using their own novel system of fluorescence-based competitive allele-specific polymerase chain reaction (KASPar). The call rates for all genotyped SNPs were >95%. Study characteristics, genotyping platform, imputation and association test software used, as well as sample and genotyping and imputation quality control steps in each stage 1 study are given in table S2. We used the same methods as in stage 1 for sample selection, genotyping quality control, association testing, and meta-analysis.

### Combined analysis of stage 1 and stage 2 samples

All stage 1 and 2 results were meta-analyzed using the inverse-variance fixed-effects method in either METAL (*41*) or R (version 3.2.0; www.r-project.org/). In these combined analyses, loci reaching *P* < 5 × 10^−8^ were considered as genome-wide significant, and loci reaching *P* < 5 × 10^−6^ were considered as a suggestive association. Heterogeneity between studies was tested by Cochran’s *Q* tests, and the proportion of variance due to heterogeneity was assessed using *I*^2^ index for each individual SNP at each stage.

### Estimation of genetic variance explained

The variance explained (*h*^2^) by each SNP was calculated using the risk allele frequency (*f*) and beta (β) from the meta-analyses using the formula *h*^2^ = β^2^ (1 − *f*)2*f*.

### Stage 1 (discovery stage) GWAS FDR

We applied Efron’s method (*45*) to the *P* values of all common SNPs (MAF > 1%) in stage 1 GWAS to estimate the FDR of the prioritized SNPs.

### Bias-reducing correction for winner’s curse

We applied the method of Zhong and Prentice (*46*) to the eight signals prioritized in our discovery GWAS and reported bias-reduced estimates for the following discovery cutoffs *P* < 10^−5^, *P* < 10^−6^, and *P* < 10^−7^.

### GWAS multiple testing correction for six phenotypes

The standard joint meta-analysis genome-wide significance cutoff (α = 5 × 10^−8^) was corrected using Bonferroni method for the number of independent tests. First, we applied the method of Li and Ji (*47*) to the phenotypic correlation matrix of the NFBC1966 (*N* > 2500) to estimate the number of “independent” phenotypes (*m* = 5). The corrected cutoff was estimated with Bonferroni method applied to five independent tests (α’ = α /5 = 10^−8^).

### Analysis of the phenotypic effects of the lead GWAS SNPs in published genetic studies

The phenotypic implication of the lead GWAS SNP on 778 phenotypes was obtained from the Gene ATLAS (*9*) PheWAS conducted on 452,264 white British individuals from UK Biobank available at http://geneatlas.roslin.ed.ac.uk. In addition, we looked up the lead GWAS SNP or a proxy on published large-scale GWAS datasets using PhenoScanner (*10*) available at www.phenoscanner.medschl.cam.ac.uk/phenoscanner. Only proxy SNPs from the 1000 genomes panel in high LD (*R*^2^ > 0.8) with the lead GWAS SNP were considered. We then searched for all SNPs with phenotypic associations in the same chromosomal region of our lead GWAS signals using the GWAS catalog (*48*) available at www.ebi.ac.uk/gwas/. We reported associations obtained from these analyses with significance cutoff *P* < 5 × 10^−8^. Last, we searched for non-GWAS genetic studies, i.e., family, pedigree, and clinical studies with PubMed queries available at www.ncbi.nlm.nih.gov/pubmed/.

### Conditional analyses

We conducted conditional analysis of the lead and proxy SNP expected dosages using a linear regression model adjusted for sex and GA in 3459 children from the NFBC1966 study. We considered two models to assess the effect of both SNPs. First, the early growth trait is regressed on the lead SNP adjusting for the study covariates sex and GA. Second, the proxy SNP is added to the previous model. The lead and proxy SNP effects are considered independent if the effect size estimate of the lead SNP in model 2 did not vary more than 20% of the effect estimate of model 1, and the corresponding *P* value reached a nominal significance (α = 0.05).

### Variance effect prediction analysis

We obtained information about the putative effect of the lead GWAS SNPs using VEP tool (*49*) available at www.ensembl.org/Homo_sapiens/Tools/VEP. The analysis included pathogenicity, splicing, and conservation predictions as well as regulatory annotations.

### Overlap of the genetic makeup of early growth traits with adult and childhood phenotypes

To gain insights into the potential overlap in the genetic makeup of early growth traits with adult and childhood phenotypes, we searched databases and the literature for the phenotypic implications of our four GWAS SNPs. First, we retrieved from the Gene Atlas (*9*) PheWAS in the UK Biobank data all phenotypic associations (*P* < 5 × 10^−8^) with our four GWAS SNPs (table S4). Second, we retrieved from the PhenoScanner (*10*) database all SNPs in the literature with phenotypic associations (*P* < 5 × 10^−8^) and in high LD (*R*^2^ > 0.8) with our four GWAS lead variants (table S5). Third, we systematically searched in the GWAS catalog (*48*) database all SNPs with phenotypic associations (*P* < 5 × 10^−8^) in the chromosomal regions of our four GWAS lead variants.

### Bayesian colocalization

Colocalization analyses were performed using our stage 1 GWAS results with multitissue eQTL results from GTEx data (www.gtexportal.org/home/datasets) (*15*). For each GTEx tissue (*n* = 48 tissues), we first identified all genes with significant cis-eQTLs at <5% of FDR. For each such gene, we retrieved the GWAS summary statistics for each of the three traits (BMI-AP, BMI-AR, and Age-AR), for all SNPs in common between the GWAS and the eQTL data [typically everything within 1 megabase (Mb) of the gene transcription start site]. If the GWAS locus contained one or more eQTL variant at *P* < 5 × 10^−6^, then we implemented the computational procedure outlined in the *coloc* package in R (https://github.com/chr1swallace/coloc/blob/master/R/coloc-package.R) (*50*) with default parameters and using the MAFs of European ancestry individuals from 1000 Genomes study.

### Expression quantitative trait locus

We searched for cis-eQTLs in liver, skin, whole blood, subcutaneous fat, and omental fat ex vivo tissues made available by the MuTHER (*51*), KORA (*52*), DeCode (*53*), Lee Kaplan (*54*), and BIOS (*55*) studies. The association analyses were performed with the GWAS lead SNP following the procedure described previously (*56*). The analysis of eQTLs was limited to genes in cis within a ±1-Mb window of the lead SNP. For each GWAS lead SNP, we separately reported the top eQTL in the locus and the coincident cis-eQTLs with significance cutoffs of *P* < 1 × 10^−3^ and an FDR of <5%, respectively. For studies where gene expression was measured using a microarray technology, the microarray probes were annotated with information accessed on ProbeDB (available at www.ncbi.nlm.nih.gov/probe/). If the probe IDs were not available in the ProbeDB, then we aligned the probe sequence to HG38 with blast algorithm available at https://blast.ncbi.nlm.nih.gov/Blast.cgi and then annotated the transcripts overlapping the genomic coordinates using consensual information in GenBank, RefSeq, ENCODE (Encyclopedia of DNA Elements), and UCSC (University of California, Santa Cruz) databases.

### Methylation quantitative trait locus

We searched for cis-methylation QTL in blood at five different life stages using mQTLdb (www.mqtldb.org/). Methylation QTL data were generated as previously described (*16*) using ALSPAC study data. Only GWAS SNPs that colocalized with eQTL data were looked up.

### Genetic correlations using LD score regression analyses

We used the LD hub (*57*) available at http://ldsc.broadinstitute.org to quantify the genetic correlation between each of the six early growth traits and a selection of 49 disease/traits of interest from 33 GWAS studies in the following precompiled categories: education, anthropometric traits, lipids, glycemic traits, bone mineral density, neurological/psychiatric diseases, and other traits (including adiponectin, coronary artery disease, type 2 diabetes, and menarche). We carried out the LD score regression analyses for blood pressure traits using the Python scripts provided on the developer’s website at https://github.com/bulik/ldsc. Before running the LD score regression analyses, each summary statistics file was reformatted using the munge_sumstats.py Python script, which filtered the SNPs to HapMap 3 SNPs as recommended on the developer’s website to minimize any bias from poor imputation quality. SNPs were also excluded if an MAF of <0.01, ambiguous strand, duplicate rsID, and reported sample size are less than 60% of the total available. If the sample size for each SNP was available, then we used the –N-col to specify the relevant sample size column in the GWAS summary statistics file, and when no sample size column was available, we used the maximum sample size reported in the GWAS meta-analysis. After the GWAS summary statistics files were reformatted, we then used the ldsc.py Python script to run the LD score regression analyses between each of the six early growth traits and systolic blood pressure and diastolic blood pressure. The precomplied European LD scores calculated from 1000 Genomes data available on the developer’s website were used for LD score regression.

### Adult BMI GRS

We calculated a weighted GRSs of adult BMI with the 97 SNPs associated with BMI at genome-wide levels of significance in the GIANT consortium (*18*) using the R package *gtx* and following the procedure described in (*58*). Briefly, a risk score estimating the pleiotropic effect of adult BMI variants on each early growth trait was inferred from summary statistics obtained from the stage 1 GWAS meta-analyses. Risk score models with evidence of heterogeneity (*P*_het_ < 0.05, only BMI-AP) were refitted using a downwards elimination of SNPs with largest effect size until the model is not heterogeneous (*P*_het_ > 0.05). In addition, we estimated evidence of horizontal pleiotropy between adult BMI and each early growth trait with the package MR-PRESSO (*59*).

### Pathway enrichment analysis

To explore the pathways associated with early growth traits, we applied MAGENTA (version 2) (*19*) to the stage 1 GWAS results. Briefly, each gene in the genome was mapped to a single SNP with the lowest *P* value within a 110-kb upstream or 40-kb downstream window of the gene. The corresponding *P* value, representing each gene, was corrected for confounding factors such as gene size, LD patterns, SNP density, and other genetic factors. The adjusted *P* values were ranked, and the observed number of genes in a given pathway above a specified *P* value threshold (75th and 95th percentiles used) was calculated. This number was compared with that from repeating the process based on 10,000 randomly permuted pathways of identical size. In doing so, an empirical gene set enrichment association (GSEA) *P* value for each pathway was computed. In our study, individual pathways with an FDR of <0.05 and nominal GSEA *P* < 0.05 were deemed significant, and, unless otherwise stated, results for the 95th percentile cutoff analysis were reported.

### SNP heritability

We estimated the SNP heritability, the proportion of variance explained by common SNPs (MAF > 1%), with LD score as implemented in LD hub and using our six stage 1 GWAS meta-analyses on early growth traits (see the next paragraph for detailed information on postprocessing of GWAS data for LD score analysis). LD score regression estimates were obtained using a regression model with intercept, which aims at correcting for systematic confounders in GWAS summary statistics such as population stratification. In addition, we provided SumHer (*21*) SNP heritability estimates due to the current debate on the (mainly downward) bias of LD score regression estimates. SumHer estimates were obtained using a regression model including an intercept. The estimates of heritability using family and twin studies were obtained from the literature using PubMed searches complemented with Google scholar. The proportion of heritability explained by common SNPs is the ratio of the SNP heritability obtained from LD score regression and the overall heritability obtained from family and twin studies.

### Post hoc power analysis

We conducted a post hoc power analysis to determine the effect size in SD units we are powered to detect (power, 80%). The following experimental setup is considered to parameterize the null hypothesis: stage 1 meta-analysis sample size (*n* = 6222), the smallest minimum allele frequency observed among the four lead GWAS SNPs (MAF = 0.22; most conservative), imputation quality *R*^2^ = 0.8, significance level *P* < 5 × 10^−8^, and genotypes assumed to be in HWE. Analysis was conducted as previously described (*60*). Briefly, the noncentrality parameter (NCP) gives the expected value of the test statistic under the null hypothesis parameterized above. The power to detect an effect size *b* > NCP is the probability of obtaining an effect larger or equal to NCP under the alternative hypothesis parameterized by the normal distribution with mean *b* and SD set equal to the SE of NCP.

## Supplementary Material

http://advances.sciencemag.org/cgi/content/full/5/9/eaaw3095/DC1

Download PDF

Table S1

Table S4

Table S12

GWAS on longitudinal growth traits reveals different genetic factors influencing infant, child, and adult BMI

## SUPPLEMENTARY MATERIALS

Supplementary material for this article is available at http://advances.sciencemag.org/cgi/content/full/5/9/eaaw3095/DC1 Table S1. Study characteristics, exclusions, genotyping, quality control, and imputation of stage 1 studies. Table S2. Study characteristics, exclusions, genotyping, and quality control in stage 2 studies. Table S3. The SNP selection criteria used for selecting loci from stage 1 GWAS meta-analysis data and proxies used for follow-up in stage 2. Table S4. The association of the four genome-wide significant SNPs or proxies in high LD (*R*^2^ > 0.8) with other phenotypes in published GWASs retrieved from PhenoScanner database. Table S5. The association of the four genome-wide significant SNPs with other phenotypes in the Gene Atlas PheWAS on the UK Biobank data. Table S6. Conditional analysis in the NFBC1966 data (*N* = 2585) of the BMI-AP association with the lead GWAS SNP rs9436303 adjusting for the early-onset obesity SNP rs11208659. Table S7. Variant effect prediction of the four genome-wide significant SNPs. Table S8. Biological and functional mechanisms of the nearest genes to the four genome-wide significant SNPs. Table S9. Top eQTLs (FDR < 1%) in five ex vivo tissues in high LD (*R*^2^ > 0.8) with the lead GWAS SNPs in *LEPR/LEPROT* locus. Table S10. Direct lookup on eQTL (*P* < 0.001) data in five ex vivo tissues of the lead GWAS SNPs in *LEPR/LEPROT* locus. Table S11. Direct lookup of the lead GWAS SNPs in LEPR/LEPROT and TFAP2B locus on methylation QTL (FDR < 1%) in blood drawn at five different life stages: mother’s pregnancy (~29.2 years, SD = 4.4 years) and middle age (~47.5 years, SD = 4.5 years), and offspring’s birth (0 years), childhood (~7.5 years, SD = 0.15 years), and adolescence (~17.1 years, SD = 1.0 years). Table S12. Cross-trait genetic correlations between five early growth traits and 80 other GWAS phenotypes from LD score regression analyses. Table S13. The directional consistency between phenotypic and genetic correlations for the same trait. Table S14. Lookup of the GIANT consortium BMI-associated SNPs on the stage 1 GWAS meta-analyses of the six early growth traits. Table S15. The GRS of adult BMI using SNP weights from the GIANT consortium applied to the early growth trait summary statistics from the stage 1 GWAS meta-analyses. Table S16. Gene set enrichment analysis (MAGENTA) of biological pathways based on the discovery GWAS. Table S17. Detailed description of IGF-1 signaling pathway associated with AGE-AR (FDR < 0.05) in MAGENTA gene set enrichment analysis. Table S18. SNP heritability of the early growth traits estimated with SumHer and LD score. Table S19. Individual contributions of authors. Fig. S1. Graphical illustration of height and weight growth patterns and the derived measures of early growth traits used in the present study. Fig. S2. Summary of study design. Fig. S3. The participating studies with their geographical location. Fig. S4. The Manhattan plot and quantile-quantile plot of the association *P* values for the six early growth phenotypes from stage 1 genome-wide association analyses. Fig. S5. Regional association and forest plots of the three genome-wide significant loci associated with early growth traits that have been previously linked with adult BMI. Fig. S6. Heterogeneity analyses of the GWAS lead SNP rs9436303 at *LEPR/LEPROT* locus. Fig. S7. Regional plots of the GWAS and GTEx cis-eQTL data used in the colocalization analysis of the early growth–associated loci. Fig. S8. Tissue-specific PPs of colocalization of *TFAP2B*. Fig. S9. Genomic annotation analysis of the colocalized variants involved in the regulation of *LEPR*, *LEPROT*, and *TFAP2B* gene expression. Fig. S10. Adult BMI GRS analysis of early growth traits. Note S1. Literature search for epidemiological associations between early growth traits and childhood and adult traits. Note S2. Cohort description (see also tables S1 and S2 for genotyping details and figs. S1 to S3). Note S3. Funding and acknowledgments by the study. References (*61*–*104*)
