# Supplementary material for: GWAS on longitudinal growth traits reveals different genetic factors influencing infant, child, and adult BMI
Source: Sci Adv. 2019 Sep 4;5(9):eaaw3095. doi: 10.1126/sciadv.aaw3095 (PMC6904961; doi:10.1126/sciadv.aaw3095)
Supplement: http://advances.sciencemag.org/cgi/content/full/5/9/eaaw3095/DC1 [file supp_5_9_eaaw3095__index.html]

Science Advances | Science AdvancesAAASSearchScience AdvancesMenu

## Supplementary Materials

**The PDF file includes:**

- Legends for tables S1 and S2
- Table S3. The SNP selection criteria used for selecting loci from stage 1 GWAS meta-analysis data and proxies used for follow-up in stage 2.
- Legends for tables S4 and S5
- Table S6. Conditional analysis in the NFBC1966 data (*N* = 2585) of the BMI-AP association with the lead GWAS SNP rs9436303 adjusting for the early-onset obesity SNP rs11208659.
- Legend for table S7
- Table S8. Biological and functional mechanisms of the nearest genes to the four genome-wide significant SNPs.
- Table S9. Top eQTLs (FDR < 1%) in five ex vivo tissues in high LD (*R*2 > 0.8) with the lead GWAS SNPs in LEPR/LEPROT locus.
- Table S10. Direct lookup on eQTL (*P* < 0.001) data in five ex vivo tissues of the lead GWAS SNPs in LEPR/LEPROT locus.
- Legends for tables S11 and S12
- Table S13. The directional consistency between phenotypic and genetic correlations for the same trait.
- Legends for tables S14 and S15
- Table S16. Gene set enrichment analysis (MAGENTA) of biological pathways based on the discovery GWAS.
- Table S17. Detailed description of IGF-1 signaling pathway associated with AGE-AR (FDR < 0.05) in MAGENTA gene set enrichment analysis.
- Legends for tables S18 and S19
- Fig. S1. Graphical illustration of height and weight growth patterns and the derived measures of early growth traits used in the present study.
- Fig. S2. Summary of study design.
- Fig. S3. The participating studies with their geographical location.
- Fig. S4. The Manhattan plot and quantile-quantile plot of the association *P* values for the six early growth phenotypes from stage 1 genome-wide association analyses.
- Fig. S5. Regional association and forest plots of the three genome-wide significant loci associated with early growth traits that have been previously linked with adult BMI.
- Fig. S6. Heterogeneity analyses of the GWAS lead SNP rs9436303 at *LEPR/LEPROT* locus.
- Fig. S7. Regional plots of the GWAS and GTEx cis-eQTL data used in the colocalization analysis of the early growth–associated loci.
- Fig. S8. Tissue-specific PPs of colocalization of *TFAP2B*.
- Fig. S9. Genomic annotation analysis of the colocalized variants involved in the regulation of LEPR, LEPROT, and TFAP2B gene expression.
- Fig. S10. Adult BMI GRS analysis of early growth traits.
- Note S1. Literature search for epidemiological associations between early growth traits and childhood and adult traits.
- Note S2. Cohort description (see also tables S1 and S2 for genotyping details and figs. S1 to S3).
- Note S3. Funding and acknowledgments by the study.
- References (*61*–*104*)

Download PDF

**Other Supplementary Material for this manuscript includes the following:**

- Table S1 (Microsoft Excel format). Study characteristics, exclusions, genotyping, quality control, and imputation of stage 1 studies.
- Table S2 (Microsoft Excel format). Study characteristics, exclusions, genotyping, and quality control in stage 2 studies.
- Table S4 (Microsoft Excel format). The association of the four genome-wide significant SNPs or proxies in high LD (*R*2 > 0.8) with other phenotypes in published GWASs retrieved from PhenoScanner database.
- Table S5 (Microsoft Excel format). The association of the four genome-wide significant SNPs with other phenotypes in the Gene Atlas PheWAS on the UK Biobank data.
- Table S7 (Microsoft Excel format). Variant effect prediction of the four genome-wide significant SNPs.
- Table S11 (Microsoft Excel format). Direct lookup of the lead GWAS SNPs in LEPR/LEPROT and TFAP2B locus on methylation QTL (FDR < 1%) in blood drawn at five different life stages: mother’s pregnancy (~29.2 years, SD = 4.4 years) and middle age (~47.5 years, SD = 4.5 years), and offspring’s birth (0 years), childhood (~7.5 years, SD = 0.15 years), and adolescence (~17.1 years, SD = 1.0 years).
- Table S12 (Microsoft Excel format). Cross-trait genetic correlations between five early growth traits and 80 other GWAS phenotypes from LD score regression analyses.
- Table S14 (Microsoft Excel format). Lookup of the GIANT consortium BMI-associated SNPs on the stage 1 GWAS meta-analyses of the six early growth traits.
- Table S15 (Microsoft Excel format). The GRS of adult BMI using SNP weights from the GIANT consortium applied to the early growth trait summary statistics from the stage 1 GWAS meta-analyses.
- Table S18 (Microsoft Excel format). SNP heritability of the early growth traits estimated with SumHer and LD score.
- Table S19 (Microsoft Excel format). Individual contributions of authors.

**Files in this Data Supplement:**

- Adobe PDF - aaw3095\_SM.pdf
- Table S4
- Table S12
- Table S1, S2, S5, S7, S11, S14, S15, S18, S19
