# Supplementary material for: GWAS on longitudinal growth traits reveals different genetic factors influencing infant, child, and adult BMI
Source: Sci Adv. 2019 Sep 4;5(9):eaaw3095. doi: 10.1126/sciadv.aaw3095 (PMC6904961; doi:10.1126/sciadv.aaw3095)
Supplement: Download PDF [file aaw3095_SM.pdf]

Science Advances
AAAS

advances.sciencemag.org/cgi/content/full/5/9/eaaw3095/DC1

Supplementary Materials for

GWAS on longitudinal growth traits reveals different genetic factors influencing infant, child, and adult BMI

Alexessander Couto Alves*, N. Maneka G. De Silva*, Ville Karhunen, Ulla Sovio, Shikta Das, H. Rob Taal, Nicole M. Warrington, Alexandra M. Lewin, Marika Kaakinen, Diana L. Cousminer, Elisabeth Thiering, Nicholas J. Timpson, Tom A. Bond, Estelle Lowry, Christopher D. Brown, Xavier Estivill, Virpi Lindi, Jonathan P. Bradfield, Frank Geller, Doug Speed, Lachlan J. M. Coin, Marie Loh, Sheila J. Barton, Lawrence J. Beilin, Hans Bisgaard, Klaus Bønnelykke, Rohia Alili, Ida J. Hatoum, Katharina Schramm, Rufus Cartwright, Marie-Aline Charles, Vincenzo Salerno, Karine Clément, Annique A. J. Claringbould, BIOS Consortium, Cornelia M. van Duijn, Elena Moltchanova, Johan G. Eriksson, Cathy Elks, Bjarke Feenstra, Claudia Flexeder, Stephen Franks, Timothy M. Frayling, Rachel M. Freathy, Paul Elliott, Elisabeth Widén, Hakon Hakonarson, Andrew T. Hattersley, Alina Rodriguez, Marco Banterle, Joachim Heinrich, Barbara Heude, John W. Holloway, Albert Hofman, Elina Hyppönen, Hazel Inskip, Lee M. Kaplan, Asa K. Hedman, Esa Läärä, Holger Prokisch, Harald Grallert, Timo A. Lakka, Debbie A. Lawlor, Mads Melbye, Tarunveer S. Ahluwalia, Marcella Marinelli, Iona Y. Millwood, Lyle J. Palmer, Craig E. Pennell, John R. Perry, Susan M. Ring, Markku J. Savolainen, Fernando Rivadeneira, Marie Standl, Jordi Sunyer, Carla M. T. Tiesler, Andre G. Uitterlinden, William Schierding, Justin M. O'Sullivan, Inga Prokopenko, Karl-Heinz Herzig, George Davey Smith, Paul O'Reilly, Janine F. Felix, Jessica L. Buxton, Alexandra I. F. Blakemore, Ken K. Ong, Vincent W. V. Jaddoe, Struan F. A. Grant*, Sylvain Sebert*, Mark I. McCarthy*, Marjo-Riitta Järvelin*, Early Growth Genetics (EGG) Consortium

*Corresponding author. Email: m.jarvelin@imperial.ac.uk (M.-R.J.); mark.mccarthy@drl.ox.ac.uk (M.I.M.); grants@chop.edu (S.F.A.G.); sylvain.sebert@oulu.fi (S.S.)

Published 4 September 2019, *Sci. Adv.* **5**, eaaw3095 (2019)
DOI: 10.1126/sciadv.aaw3095

The PDF file includes:

Legends for tables S1 and S2
Table S3. The SNP selection criteria used for selecting loci from stage 1 GWAS meta-analysis data and proxies used for follow-up in stage 2.
Legend for tables S4 and S5
Table S6. Conditional analysis in the NFBC1966 data ($N = 2585$) of the BMI-AP association with the lead GWAS SNP rs9436303 adjusting for the early-onset obesity SNP rs11208659.
Legend for table S7
Table S8. Biological and functional mechanisms of the nearest genes to the four genome-wide significant SNPs.
Table S9. Top eQTLs (FDR <1%) in five ex vivo tissues in high LD ($R^2 < 0.8$) with the lead GWAS SNPs in *LEPR/LEPROT* locus.
Table S10. Direct lookup on eQTL ($P < 0.001$) data in five ex vivo tissues of the lead GWAS SNPs in *LEPR/LEPROT* locus.
Legends for tables S11 and S12

Table S13. The directional consistency between phenotypic and genetic correlations for the same trait.
Legends for tables S14 and S15
Table S16. Gene set enrichment analysis (MAGENTA) of biological pathways based on the discovery GWAS.
Table S17. Detailed description of IGF-1 signaling pathway associated with AGE-AR (FDR < 0.05) in MAGENTA gene set enrichment analysis.
Legends for tables S18 and S19
Fig. S1. Graphical illustration of height and weight growth patterns and the derived measures of early growth traits used in the present study.
Fig. S2. Summary of study design.
Fig. S3. The participating studies with their geographical location.
Fig. S4. The Manhattan plot and quantile-quantile plot of the association *P* values for the six early growth phenotypes from stage 1 genome-wide association analyses.
Fig. S5. Regional association and forest plots of the three genome-wide significant loci associated with early growth traits that have been previously linked with adult BMI.
Fig. S6. Heterogeneity analyses of the GWAS lead SNP rs9436303 at *LEPR/LEPROT* locus.
Fig. S7. Regional plots of the GWAS and GTEx cis-eQTL data used in the colocalization analysis of the early growth–associated loci.
Fig. S8. Tissue-specific PPs of colocalization of *TFAP2B*.
Fig. S9. Genomic annotation analysis of the colocalized variants involved in the regulation of *LEPR*, *LEPROT*, and *TFAP2B* gene expression.
Fig. S10. Adult BMI GRS analysis of early growth traits.
Note S1. Literature search for epidemiological associations between early growth traits and childhood and adult traits
Note S2. Cohort description (see also tables S1 and S2 for genotyping details and figs. S1 to S3)
Note S3. Funding and acknowledgments by the study
References (*61–104*)

Other Supplementary Material for this manuscript includes the following:

(available at advances.sciencemag.org/cgi/content/full/5/9/eaaw3095/DC1)

Table S1 (Microsoft Excel format). Study characteristics, exclusions, genotyping, quality control, and imputation of stage 1 studies.
Table S2 (Microsoft Excel format). Study characteristics, exclusions, genotyping, and quality control in stage 2 studies.
Table S4 (Microsoft Excel format). The association of the four genome-wide significant SNPs or proxies in high LD ($R^2 > 0.8$) with other phenotypes in published GWASs retrieved from PhenoScanner database.
Table S5 (Microsoft Excel format). The association of the four genome-wide significant SNPs with other phenotypes in the Gene Atlas PheWAS on the UK Biobank data.
Table S7 (Microsoft Excel format). Variant effect prediction of the four genome-wide significant SNPs.
Table S11 (Microsoft Excel format). Direct lookup of the lead GWAS SNPs in LEPR/LEPROT and TFAP2B locus on methylation QTL (FDR < 1%) in blood drawn at five different life stages: mother's pregnancy (~29.2 years, SD = 4.4 years) and middle age (~47.5 years, SD = 4.5 years), and offspring's birth (0 years), childhood (~7.5 years, SD = 0.15 years), and adolescence (~17.1 years, SD = 1.0 years).

Table S12 (Microsoft Excel format). Cross-trait genetic correlations between five early growth traits and 80 other GWAS phenotypes from LD score regression analyses.
Table S14 (Microsoft Excel format). Lookup of the GIANT consortium BMI-associated SNPs on the stage 1 GWAS meta-analyses of the six early growth traits.
Table S15 (Microsoft Excel format). The GRS of adult BMI using SNP weights from the GIANT consortium applied to the early growth trait summary statistics from the stage 1 GWAS meta-analyses.
Table S18 (Microsoft Excel format). SNP heritability of the early growth traits estimated with SumHer and LD score.
Table S19 (Microsoft Excel format). Individual contributions of authors.

SUPPLEMENTARY TABLES

Table S1. Study characteristics, exclusions, genotyping, quality control, and imputation of stage 1 studies.

Table S2. Study characteristics, exclusions, genotyping, and quality control in stage 2 studies.

Table S3. The SNP selection criteria used for selecting loci from stage 1 GWAS meta-analysis data and proxies used for follow-up in stage 2.

Phenotype	Chr	Nearest gene	lead SNP	Proxy1[b]	Proxy2[b]	*P*-value	FDR[c]	Beta[d]	Beta corrected for Winner's curse Sensitivity analysis[e]			Justification for follow-up
									$<10^{-5}$	$<10^{-6}$	$<10^{-7}$	
**PWV**	2	*TMEM18*[a]	rs2860323	rs6548238 [r^2=0.85; P=1.6 x 10^{-4}]	rs7567570 [r^2=1; P=5.9 x 10^{-5}]	5.91 x10^{-5}	5.6 x10^{-1}	0.09	0.06	0.01	0.01	Adult and child BMI(*61, 62*) and early onset extreme obesity associated locus(*12, 63*)
**BMI-AP**	1	*LEPR/ LEPROT*	rs9436303	rs9436301 [r^2=1; P=6.4 x 10^{-8}]	rs9436302 [r^2=0.8; P=1.4 x 10^{-7}]	4.15 x 10^{-9}	4.0 x10^{-2}	0.13	0.13	0.13	0.13	*P* <1 x10^{-7}
**BMI-AP**	5	*PCSK1*	rs10515235	rs13153770 [r=0.95; P=1.3 x 10^{-6}]	rs13158163 [r^2=1; P=3.4 x 10^{-6}]	1.23 x 10^{-6}	7.3 x10^{-1}	0.09	0.08	0.05	0.01	P < 1 x 10^{-5} & severe obesity(*64*) and fasting glucose(*65*) associated locus
**Age-AR**	16	*FTO*	rs1421085	rs9939609 [r^2=0.9; P=1.6 x 10^{-7}]	rs1558902 [r^2=1; P=6.3 x 10^{-8}]	6.14 x 10^{-8}	4.0 x10^{-3}	-0.1	-0.1	-0.1	-0.06	*P* <1 x10^{-7}
**Age-AR**	5	*Intergenic RANBP3L and SLC1A3*	rs2956578	rs2956577 [r^2=1; P=6.7 x 10^{-8}]	rs2967066 [r=1; P=6.7 x 10^{-8}]	6.73 x 10^{-8}	7.0 x10^{-3}	0.11	0.11	0.11	0.08	*P* <1 x10^{-7}
**Age-AR**	6	*TFAP2B*	rs2817419	rs2635727 [r^2=0.85; P=3.2 x 10^{-6}]	rs4715208 [r^2=0.85; P=3.2 x 10^{-6}]	2.96 x 10^{-6}	5.0 x10^{-1}	-0.1	-0.1	-0.07	-0.01	*P* < 1 x 10^{-5} & BMI associated locus(*44*)
**BMI-AR**	4	*GNPDA2*	rs10938397	rs12641981 [r^2=1; P=3.1 x 10^{-6}]	rs13130484 [r^2=1; P=3.4 x 10^{-6}]	1.41 x 10^{-7}	4.0 x10^{-2}	0.09	0.07	0.01	0.01	*P* < 1 x 10^{-5} & known BMI associated locus(*66*)
**BMI-AR**	11	*DLG2*	rs2055816	rs7116340 [r^2=1; P=1.7 x 10^{-7}]	rs1354397 [r^2=1; P=1.7 x 10^{-7}]	5.41 x 10^{-6}	1.3 x10^{-1}	-0.13	-0.13	-0.13	-0.13	*P* < 1 x 10^{-5} & possible candidate implicated in glucose metabolism(*43*)

All SNPs with *P* < 1 x 10^{-7} or *P* < 1 x 10^{-5} in/near candidate genes were selected for follow-up in stage 2.

The two-stage joint-analysis design does not necessarily require FWER/FDR<0.05 in stage 1 (discovery stage).

AP=adiposity peak in infancy, AR=adiposity rebound, PWV=peak weight velocity, FDR=false discovery rate.

[a]rs2860323 in the *TMEM18* locus was selected as an exception due to its potential role in early onset severe obesity(*12, 63*) and childhood(*62*) and adult BMI(*61*).

[b]Proxy1 or Proxy2 was used in follow-up studies when the GWAS SNP was not available. Values in [] represents the linkage disequilibrium with GWAS lead SNP and the association *P*-value with the relevant early growth trait.

[c]FDR attached to the prioritized SNP *P*-value. FDR estimated on stage 1 (discovery stage) GWAS meta-analysis data (see **Methods**).

[d]Beta of the SNP association with the phenotype in the stage 1 (discovery stage) GWAS meta-analysis

[e]Bias-corrected effect sizes (conservative estimates) for winner's curse as a function of the P-value cut-offs P < {10^{-5}, 10^{-6}, 10^{-7}} applied to stage 1 GWAS meta-analysis.

Table S4. The association of the four genome-wide significant SNPs or proxies in high LD (R^2 > 0.8) with other phenotypes in published GWASs retrieved from PhenoScanner database.

Table S5. The association of the four genome-wide significant SNPs with other phenotypes in the Gene Atlas PheWAS on the UK Biobank data. UK Biobank is a sample of UK adults aged between 40 and 69 years. Childhood phenotypes are questionnaire-based and were ascertained retrospectively.

Table S6. Conditional analysis in the NFBC1966 data (N = 2585) of the BMI-AP association with the lead GWAS SNP rs9436303 adjusting for the early-onset obesity SNP rs11208659.

SNP	Position	*Effect allele/other allele*	Frequency of effect allele	Model 1[a]				Model 2[b]			
				β	95% CI		*P*	β	95% CI		*P*
**rs9436303**	35868592	G/A	0.22	0.20	0.13	0.28	*2.22×10^{-7}*	0.19	0.11	0.27	*2.87×10^{-6}*
rs11208659	35951198	C/T	0.08	-0.08	-0.18	0.02	*0.10*	-0.08	-0.18	0.02	*0.11*

[a]Model 1: z[log]BMI at AP=SNP + GA + SEX. Individual effect of each SNP was tested using a linear regression model where each SNP was included as independent variable and logBMI at AP Z-score as the dependent variable adjusted gestational age [GA] and sex.

[b]Model 2. z [log]BMI at AP=rs9436303 + GA + SEX + SNP. Conditional effect of the lead SNP rs9436303 was tested using a linear regression model where the lead SNP was included as the independent variable and logBMI at AP Z-score as the dependent variable adjusted for rs112008659.

Table S7. Variant effect prediction of the four genome-wide significant SNPs. ClinVar is the clinical significance of the effect allele obtained from the ClinVar database. LoFtool indicates the loss of function intolerance percentile, lower percentiles indicate less tolerance to the derived (effect) allele. AF is the allele frequency from the1000 genomes project. APPRIS database of transcript isoforms indicates the principal or alternative isoform based on transcript abundance. Biotype is the GENCODE annotation of the transcript or regulatory feature.

Table S8. Biological and functional mechanisms of the nearest genes to the four genome-wide significant SNPs.

GWAS lead SNP	Phenotype	Position	In/near gene	Biological function	Molecular mechanisms	Other Names
rs9436303	BMI-AP	1p31	*LEPR/LEPROT*	Cell surface receptor linked signal transduction, energy reserve metabolic process, multicellular organismal development	Hematopoietin/interferon-class cytokine receptor activity, protein binding	*CD295; OBR*
rs1421085	Age-AR	16q12.2	*FTO*	RNA methylation; regulation of lipid storage	Unknown	*FTO; KIAA1752; MGC5149*
rs2817419	Age-AR	6p12	*TFAP2B*	Kidney development, nervous system development, regulation of transcription from RNA polymerase II promoter, transcription	Protein dimerization activity, sequence-specific DNA binding, transcription co-activator activity	*TFAP2B; AP-2B; AP2-B; MGC21381*
rs10938397	BMI-AR	4p12	*GNPDA2*	N-acetylglucosamine metabolic process, carbohydrate metabolism	Glucosamine-6-phosphate deaminase activity, isomerase activity	*GNPDA2; SB52*

LEPR/LEPROT: Leptin receptor/leptin receptor overlapping transcript*; FTO:* Fat mass and obesity associated*; TFAP2B:* Transcription factor AP-2 beta [activating enhancer binding protein 2 beta]*; GNPDA2:* Glucosamine-6-phosphate deaminase 2, BMI-AP (BMI at Adiposity peak), Age-AR (Age at Adiposity Rebound) & BMI-AR (BMI at Adiposity Rebound).
Biological function and molecular mechanisms are based on information given on Gene Ontology Consortium database [http://geneontology.org].

Table S9. Top eQTLs (FDR < 1%) in five ex vivo tissues in high LD (R^2 < 0.8) with the lead GWAS SNPs in *LEPR/LEPROT* locus.

GWAS lead SNP	Best eQTL marker	eQTL gene	Transcript	Best eQTL *P*	R^2	Tissue	Study
rs9436303	rs9436301	*LEPROT*	AK023598	2.00×10^{-90}	0.98	Subcutaneous fat	deCODE
rs9436303	rs9436303	*LEPROT*	AK023598	4.51×10^{-12}	1	Omental fat	Kaplan
rs9436303	rs9436745*	*LEPR*	NA	1.77×10^{-50}	0.83	Whole Blood	BIOS

R^2 refers to the linkage disequilibrium between GWAS and eQTL SNPs

Only associations where the eQTL and lead GWAS SNP showed a R^2>=0.8 are reported

Table S10. Direct lookup on eQTL (*P* < 0.001) data in five ex vivo tissues of the lead GWAS SNPs in *LEPR/LEPROT* locus.

GWAS lead SNP	Tissue	Study	Nearest gene to locus$	Chr.	EA/OA	MAF	Direction of association for EA	eQTL Gene	Transcript*	*eQTL P-value*
rs9436303	**Liver**	**Kaplan**	*LEPR/LEPROT*	1	G/A	0.19	-	*LEPROT*	AK023598	1.38×10^{-12}
rs9436303	**Liver**	**Kaplan**	*LEPR/LEPROT*	1	G/A	0.19	+	*LEPROT*	NM_017526	1.50×10^{-9}
rs9436303	**Omental fat**	**Kaplan**	*LEPR/LEPROT*	1	G/A	0.19	-	*LEPROT*	AK023598	4.51×10^{-12}
rs9436303	**Omental fat**	**Kaplan**	*LEPR/LEPROT*	1	G/A	0.19	+	*LEPR*	BC036581	4.83×10^{-4}
rs9436303	**Omental fat**	**Kaplan**	*LEPR/LEPROT*	1	G/A	0.19	+	*LEPROT*	NM_017526	2.81×10^{-12}
rs9436303	**Subcutaneous fat**	**Kaplan**	*LEPR/LEPROT*	1	G/A	0.19	-	*LEPROT*	AK023598	2.24×10^{-4}
rs9436303	**Subcutaneous fat**	**Kaplan**	*LEPR/LEPROT*	1	G/A	0.19	+	*LEPR*	BC036581	1.98×10^{-5}
rs9436303	**Subcutaneous fat**	**Kaplan**	*LEPR/LEPROT*	1	G/A	0.19	+	*LEPROT*	NM_017526	1.76×10^{-9}
rs9436303	**Whole Blood**	**Kora**	*LEPR/LEPROT*	1	G/A	0.19	+	*LEPR*	NM_001003679	1.59×10^{-6}
rs9436303	**Whole Blood**	**Kora**	*LEPR/LEPROT*	1	G/A	0.19	+	*LEPR*	NM_001003679	6.39×10^{-6}
rs9436303	**Whole Blood**	**Kora**	*LEPR/LEPROT*	1	G/A	0.19	+	*LEPROT*	NM_017526	3.67×10^{-11}
rs9436303	**Whole Blood**	**Kora**	*LEPR/LEPROT*	1	G/A	0.19	-	*SLC35D1*	NM_015139	7.58×10^{-5}
rs9436303	**Subcutaneous fat**	**deCODE**	*LEPR/LEPROT*	1	G/A	0.19	+	*LEPROT*	NM_017526	1.50×10^{-51}
rs9436303	**Subcutaneous fat**	**deCODE**	*LEPR/LEPROT*	1	G/A	0.19	-	*LEPROT*	AK023598	1.69×10^{-89}
rs9436303	**Subcutaneous fat**	**MuTHER**	*LEPR/LEPROT*	1	G/A	0.23	+	*LEPROT*	NM_017526	4.17×10^{-8}
rs9436303	**Skin**	**MuTHER**	*LEPR/LEPROT*	1	G/A	0.23	+	*LEPROT*	NM_017526	2.07×10^{-5}
rs9436303*	**Whole blood**	**BIOS**	*LEPR/LEPROT*	1	G/A	0.19	+	*LEPR*	NA	3.66×10^{-50}

Only genes located in a distance +/- 1 MB from the lead SNPs are reported. EA=Effect allele OA=Other allele. $ The lead SNP rs9436303 is in a region with two overlapping genes - *LEPR* and *LEPROT*. MAF=Minor Allele Frequency. eQTL =Expression Quantitative Trait Locus). PP=Posterior probability.NA=Not Available.

Table S11. Direct lookup of the lead GWAS SNPs in *LEPR/LEPROT*, and *TFAP2B* locus on methylation QTL (FDR < 1%) in blood drawn at five different life stages: mother's pregnancy (~29.2 years, SD = 4.4 years) and middle age (~47.5 years, SD = 4.5 years), and offspring's birth (0 years), childhood (~7.5 years, SD = 0.15 years), and adolescence (~17.1 years, SD = 1.0 years). Effect sizes is the difference in median proportion methylated between homozygote groups. Phenotypes associated with methylation probe levels are reported based on PubMed literature search of human data. h^2 is the chip SNP heritability calculated with GWAS summary statistics using SumHer and LD-score regression. The mean chi-squared denotes the mean of all GWAS SNP chi-square statistics. P denotes the significance of the heritability estimate using the Wald test

Table S12. Cross-trait genetic correlations between five early growth traits and 80 other GWAS phenotypes from LD score regression analyses. BH p denotes the Benjamini-Hochberg false discovery correction of the p value. Genetic correlations of Age-AP with other traits could not be quantified, due to low mean chi-square of the Age-AP GWAS summary statistics

Table S13. The directional consistency between phenotypic and genetic correlations for the same trait.

Trait 1	Trait 2	r_g (SE)	*P*-value	Observational association[$]
PHV	FEV_1	-0.49 (0.24)	0.04	0(*67*)
PHV	FVC	-0.51 (0.26)	0.04	0(*67*)
PWV	Childhood obesity	0.53 (0.10)	7.8×10^{-8}	+(*68*)
PWV	Birth length	0.50 (0.14)	3.0×10^{-4}	+(*69*)
PWV	Waist circumference	0.23 (0.07)	8.0×10^{-4}	+(*69*)
PWV	BMI	0.19 (0.07)	6.0×10^{-3}	+(*70*) +(*71*) +(*69*)
PWV	HDL cholesterol	-0.21 (0.09)	2.1×10^{-2}	0(*69*)
BMI-AP	Childhood obesity	0.65 (0.11)	1.2×10^{-8}	+(*68*)+(*72*)
BMI-AP	BMI	0.26 (0.07)	2.0×10^{-4}	+(*68*)+(*73*)+(*72*)+(*3*)+(*74*)*****+(*75*)*****
BMI-AP	SBP	-0.16 (0.08)	0.04	0(*3*)+(*74*)^*****+(*73*)
AGE-AR	BMI	-0.72 (0.08)	3.1×10^{-18}	-(*8*) -(*3*) -(*76*) -(*70*) -(*77*) -(*78*) -(*79*)* -(*80*)* -(*81*) -(*82*)* -(*83*)* -(*84*)^ -(*85*)* -(*86*)* -(*87*)* -(*88*)* -(*89*)* -(*90*)*
AGE-AR	Obesity class 1	-0.75 (0.11)	1.8×10^{-12}	-(*79*)* -(*91*)* -(*92*)* -(*86*)*
AGE-AR	Waist circumference	-0.62 (0.09)	8.4×10^{-12}	-(*3*) -(*8*) -(*93*) -(*80*)*
AGE-AR	Age at Menarche	0.44 (0.07)	1.9×10^{-9}	+(*91*)*
AGE-AR	Childhood obesity	-0.84 (0.11)	1.9×10^{-9}	-(*94*)*
AGE-AR	Fasting insulin	-0.58 (0.13)	8.7×10^{-6}	- (*3*)
AGE-AR	HDL cholesterol	0.27 (0.09)	2.3×10^{-3}	+(*3*) +(*77*)$* 0(*8*) 0(*93*)
AGE-AR	DBP	-0.20 (0.07)	4.4×10^{-3}	-(*8*)^ -(*95*)* -(*3*) - (*77*)$
AGE-AR	Adiponectin	-0.31 (0.11)	6.1×10^{-3}	+(*70*)^
AGE-AR	Fasting glucose	-0.24 (0.11)	0.02	-(*8*) -(*3*) 0(*93*)
AGE-AR	HOMA-IR	-0.38 (0.15)	0.01	-(*93*)
AGE-AR	SBP	-0.13 (0.06)	0.04	-(*8*)^ -(*95*)^* -(*3*) -(*77*)$ 0(*78*)
BMI-AR	BMI	0.64 (0.08)	1.6×10^{-15}	+(*3*)+(*70*)+(*85*)*+(*86*)*+(*89*)*
BMI-AR	Obesity class 1	0.66 (0.09)	2.8×10^{-13}	+(*92*)*
BMI-AR	Waist circumference	0.48 (0.08)	6.1×10^{-10}	+(*3*)
BMI-AR	Fasting insulin	0.26 (0.12)	0.02	+(*3*)

Genetic correlations were estimated with LD score regression and phenotypic correlations are reported in the literature

r_g =genetic correlation. [$]Relevant reference number reporting the observational association. “0” =no association, “+”= positive association “-“=inverse association. *****Association was adjusted for fewer or no

potential confounders. ^Associations only observed in females and $ Associations only observed in males in those studies that were sex stratified. Body Mass Index (BMI), Peak Height Velocity (PHV), Peak Weight Velocity (PWV), BMI at Adiposity Peak (BMI-AP), Age-AR (Age at Adiposity Rebound), BMI-AR (BMI at Adiposity Rebound), Forced Vital Capacity (FVC), Forced Expiratory Volume 1 (FEV_1), Systolic Blood Pressure (SBP), Diastolic Blood Pressure (DBP) & Homeostatic Model Assessment of Insulin Resistance (HOMA-IR)**.**

Table S14. Lookup of the GIANT consortium BMI-associated SNPs on the stage 1 GWAS meta-analyses of the six early growth traits.

Table S15. The GRS of adult BMI using SNP weights from the GIANT consortium applied to the early growth trait summary statistics from the stage 1 GWAS meta-analyses. N number of samples in stage 1 meta-analysis. Effect size is the effect of BMI on other early growth traits. h^2_{grs} is the additive pseudo-heritability. P denotes the significance of the BMI effect on the early growth traits using the Wald test implemented in GTX package. P_{het} denotes the significance of the BMI SNP effects heterogeneity using the test implemented in the GTX package. $P_{MR\text{-}PRESSO}$ denotes the significance of horizontal pleiotropy in Mendelian randomization with BMI SNPs as instrumental variables using MR-PRESSO package.

Table S16. Gene set enrichment analysis (MAGENTA) of biological pathways based on the discovery GWAS. FDR obtain by looking up the permutation P values at the 75 and 95 percentiles.

Phenotype	Database	Gene set	Gene set size	GSEA *P*-value [95th percentile cut-off]	FDR [95th percentile cut-off]	Expected genes > 95th percentile cut-off	Observed genes > 95 % cut-off	GSEA *P*-value [75th percentile cut-off]	FDR [75th percentile cut-off]	Expected genes > 75th percentile cut-off	Observed genes > 75th percentile cut-off
**Age-AR**	**Ingenuity**	**IGF-I signalling**	**18**	**0.002**	**0.04**	**1**	**5**	**0.06**	**0.56**	**5**	**8**
**Age-AR**	**PANTHER**	**Apolipoprotein**	**17**	**0.001**	**0.07**	**1**	**5**	**0.01**	**0.21**	**4**	**9**
**Age-AR**	PANTHER	Transporter	68	0.002	0.20	3	10	0.005	0.15	17	27
**Age-AR**	PANTHER	Actin binding cytoskeletal protein	74	0.003	0.22	4	10	0.06	0.47	19	25
**Age-AR**	PANTHER	Carbohydrate phosphatase	7	0.04	0.34	0	2	0.55	0.94	2	2
**Age-AR**	PANTHER	Vesicle coat protein	39	0.01	0.36	2	6	0.37	0.96	10	11
**Age-AR**	GO	Lipoprotein metabolic process	16	0.006	0.42	1	4	0.36	0.79	4	5
**Age-AR**	GO	Negative regulation of insulin receptor signalling pathway	16	0.007	0.45	1	4	0.19	0.68	4	6
**Age-AR**	GO	Neuromuscular process controlling balance	23	0.004	0.46	1	5	0.35	0.79	6	7
**Age-AR**	GO	Glycoprotein binding	22	0.005	0.48	1	5	0.003	0.35	6	12
**BMI-AR**	GO	Cellular response to oxidative stress	8	0.006	0.21	0	3	0.03	0.50	2	5
**BMI-AR**	GO	DNA-dependent ATPase activity	26	0.001	0.22	1	6	0.17	0.68	7	9
**BMI-AR**	Ingenuity	TR RXR activation	57	0.006	0.23	3	8	0.45	1.00	14	15
**BMI-AR**	GO	Cell fate specification	13	0.003	0.23	1	4	0.02	0.47	3	7
**BMI-AR**	GO	Transport vesicle	36	0.001	0.24	2	7	0.09	0.68	9	13
**BMI-AR**	Ingenuity	Huntington's disease signaling	60	0.003	0.26	3	9	0.09	0.77	15	20
**BMI-AR**	GO	Forebrain development	47	4.0×10^{-4}	0.26	2	9	8.0×10^{-4}	0.31	12	22
**BMI-AR**	GO	Camp biosynthetic process	13	0.002	0.26	1	4	0.07	0.60	3	6
**BMI-AR**	GO	Amino acid transmembrane transporter activity	23	0.005	0.27	1	5	0.04	0.54	6	10
**BMI-AP**	PANTHER	Signaling molecule	57	0.07	0.92	3	6	0.45	1.00	14	15
**BMI-AP**	PANTHER	Methyltransferase	83	0.02	0.93	4	9	0.03	0.88	21	29
**BMI-AP**	PANTHER	Interleukin signaling pathway	56	0.95	0.97	3	1	0.79	0.96	14	12
**BMI-AP**	PANTHER	Inflammation mediated by chemokine and cytokine signaling pathway	81	0.92	0.98	4	2	0.66	0.97	20	19
**BMI-AP**	PANTHER	Integrin signalling pathway	115	0.84	0.98	6	4	0.51	1.00	29	29
**BMI-AP**	PANTHER	Other cytoskeletal proteins	30	1.00	0.98	2	0	0.05	0.71	8	12
**BMI-AP**	PANTHER	Synaptic vesicle trafficking	29	1.00	0.98	1	0	0.16	1.00	7	10
**BMI-AP**	PANTHER	Other ion channel	36	1.00	0.98	2	0	0.04	0.71	9	14
**BMI-AP**	PANTHER	Alzheimer disease-amyloid secretase pathway	23	1.00	0.98	1	0	0.99	0.99	6	2
**BMI-AP**	PANTHER	Other RNA-binding protein	147	0.94	0.98	7	4	0.29	0.99	37	40

Results are shown for the three early growth phenotypes showing genome-wide significant associations. Gene set enrichment analysis [GSEA] *P*-value and false discovery rate [FDR] were computed for all biological gene-sets. Results are presented for the top 10 pathways ranked by FDR [95th percentile]. Age-AR (Age at Adiposity Rebound), & BMI-AR (BMI at Adiposity Rebound) & BMI-AP (BMI at Adiposity peak)

Table S17. Detailed description of IGF-1 signaling pathway associated with AGE-AR (FDR < 0.05) in MAGENTA gene set enrichment analysis.

Database	Gene set	Gene*	Gene *P*-value	Chromosome	Position	*P*-value
**Ingenuity**	IGF-I Signaling	*PXN*	0.008	12	119279035	7.01×10^{-4}
**Ingenuity**	IGF-I Signaling	*GRB10*	0.009	7	50654821	1.66×10^{-4}
**Ingenuity**	IGF-I Signaling	*JUN*	0.03	1	59020580	0.001
**Ingenuity**	IGF-I Signaling	*SRF*	0.03	6	43264137	0.002
**Ingenuity**	IGF-I Signaling	*IGF1*	0.04	12	101422482	9.42×10^{-4}
**Ingenuity**	IGF-I Signaling	*SHC1*	0.07	1	153322945	0.003
**Ingenuity**	IGF-I Signaling	*BAD*	0.23	11	63868503	0.02
**Ingenuity**	IGF-I Signaling	*RASA1*	0.25	5	86692623	0.01
**Ingenuity**	IGF-I Signaling	*CASP9*	0.29	1	15704239	0.01
**Ingenuity**	IGF-I Signaling	*PTK2*	0.33	8	142177722	0.01
**Ingenuity**	IGF-I Signaling	*IGF1R*	0.38	15	97227070	0.005
**Ingenuity**	IGF-I Signaling	*RAF1*	0.50	3	12772562	0.04
**Ingenuity**	IGF-I Signaling	*GRB2*	0.52	17	70853306	0.04
**Ingenuity**	IGF-I Signaling	*NEDD4*	0.55	15	54054124	0.02
**Ingenuity**	IGF-I Signaling	*PTPN11*	0.57	12	111390797	0.06
**Ingenuity**	IGF-I Signaling	*FOS*	0.67	14	74739401	0.03
**Ingenuity**	IGF-I Signaling	*MAPK8*	0.72	10	49268952	0.07
**Ingenuity**	IGF-I Signaling	*PDPK1*	0.94	16	2424783	0.29

***** *ELK1* gene was removed from the analysis as it was on the chromosome X and was not analyzed. Results for only the top 10 significant genes ($P < 0.05$) from each pathway are presented.

Table S18. SNP heritability of the early growth traits estimated with SumHer and LD score.

Table S19. Individual contributions of authors.

SUPPLEMENTARY FIGURES

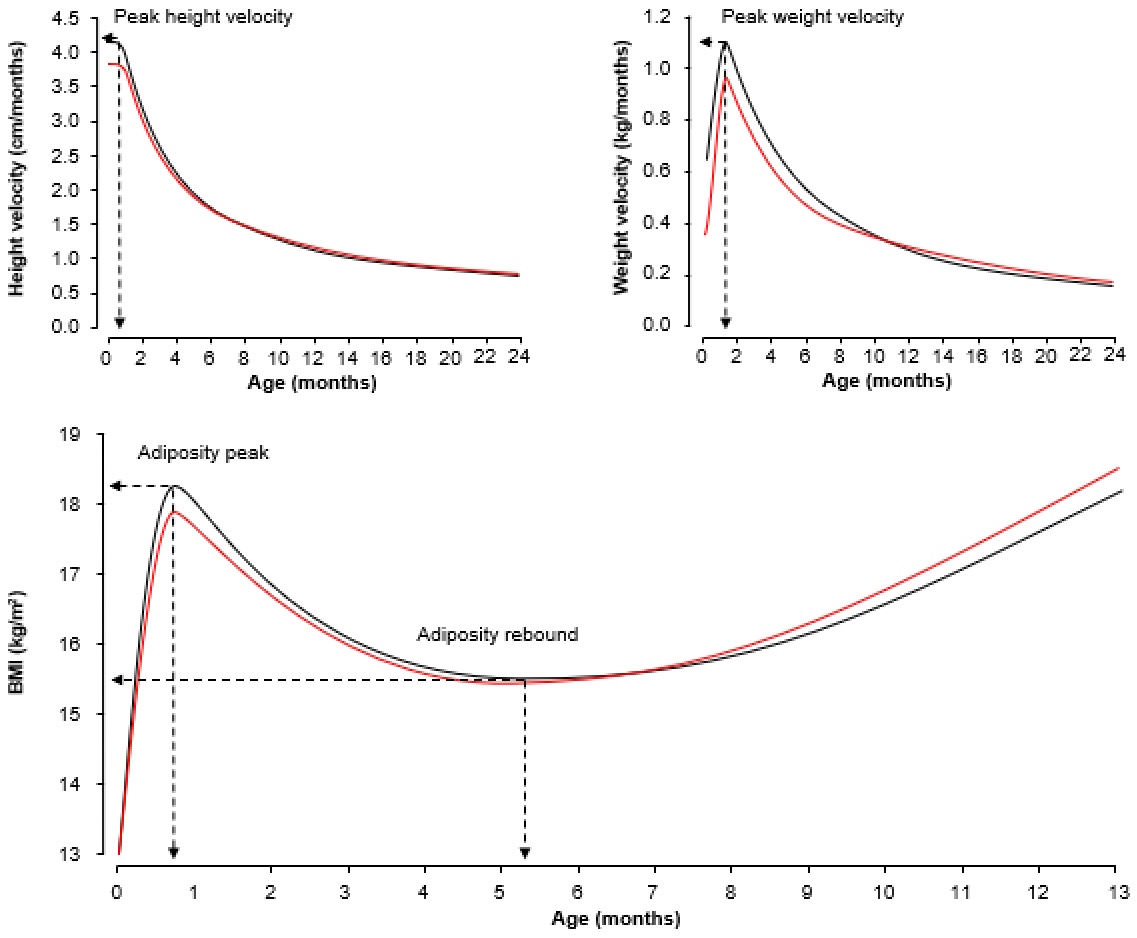

Fig. S1. Graphical illustration of height and weight growth patterns and the derived measures of early growth traits used in the present study. A) Peak Height Velocity (PHV); B) Peak Weight Velocity (PWV); C) Age and BMI at Adiposity Peak (AP) and Rebound (AR). The growth curves for males are in blue and for females in red. Curves are based on the Northern Finland Birth Cohort (NFBC) 1986 data.

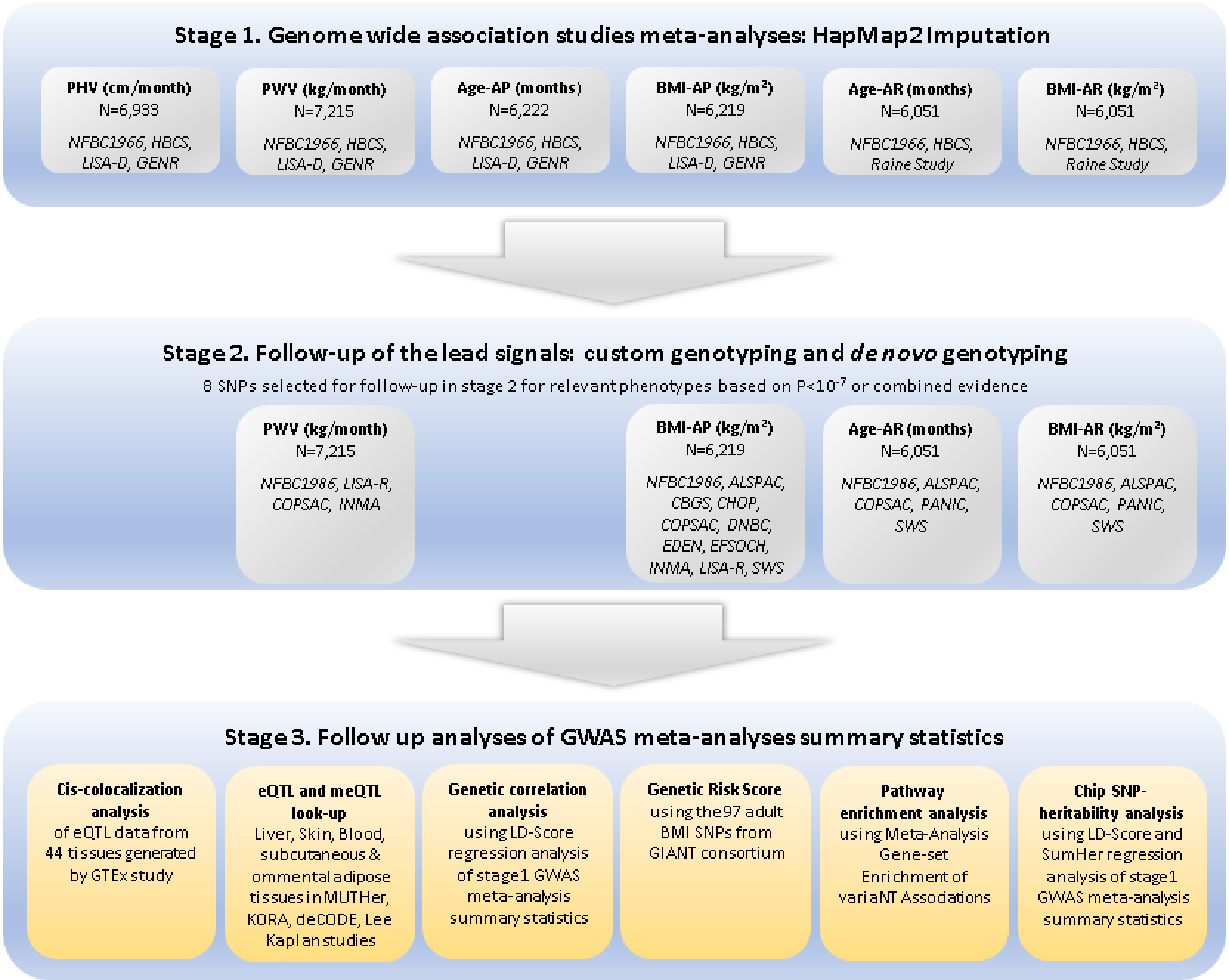
Stage 1. Genome wide association studies meta-analyses: HapMap2 Imputation
PHV (cm/month) N=6,933 NFBC1966, HBCS, LISA-D, GENR
PWV (kg/month) N=7,215 NFBC1966, HBCS, LISA-D, GENR
Age-AP (months) N=6,222 NFBC1966, HBCS, LISA-D, GENR
BMI-AP (kg/m²) N=6,219 NFBC1966, HBCS, LISA-D, GENR
Age-AR (months) N=6,051 NFBC1966, HBCS, Raine Study
BMI-AR (kg/m²) N=6,051 NFBC1966, HBCS, Raine Study
Stage 2. Follow-up of the lead signals: custom genotyping and de novo genotyping
8 SNPs selected for follow-up in stage 2 for relevant phenotypes based on P<10-7 or combined evidence
PWV (kg/month) N=7,215 NFBC1986, LISA-R, COPSAC, INMA
BMI-AP (kg/m²) N=6,219 NFBC1986, ALSPAC, CBGS, CHOP, COPSAC, DNBC, EDEN, EFSOCH, INMA, LISA-R, SWS
Age-AR (months) N=6,051 NFBC1986, ALSPAC, COPSAC, PANIC, SWS
BMI-AR (kg/m²) N=6,051 NFBC1986, ALSPAC, COPSAC, PANIC, SWS
Stage 3. Follow up analyses of GWAS meta-analyses summary statistics
Cis-colocalization analysis of eQTL data from 44 tissues generated by GTEx study
eQTL and meQTL look-up Liver, Skin, Blood, subcutaneous & ommental adipose tissues in MUTHer, KORA, deCODE, Lee Kaplan studies
Genetic correlation analysis using LD-Score regression analysis of stage1 GWAS meta-analysis summary statistics
Genetic Risk Score using the 97 adult BMI SNPs from GIANT consortium
Pathway enrichment analysis using Meta-Analysis Gene-set Enrichment of variaNT Associations
Chip SNP-heritability analysis using LD-Score and SumHer regression analysis of stage1 GWAS meta-analysis summary statistics

Fig. S2. Summary of study design. The Flowchart shows the studies and total sample size used in Stage 1 genome-wide association meta-analyses and in Stage 2 follow up for each early growth trait leading to Stage 3 follow-up analyses. Stage 1 included up to 7215 European ancestry children from up to four population-based studies and stage 2 follow up included up to 16,550 European ancestry children from up to 11 studies.

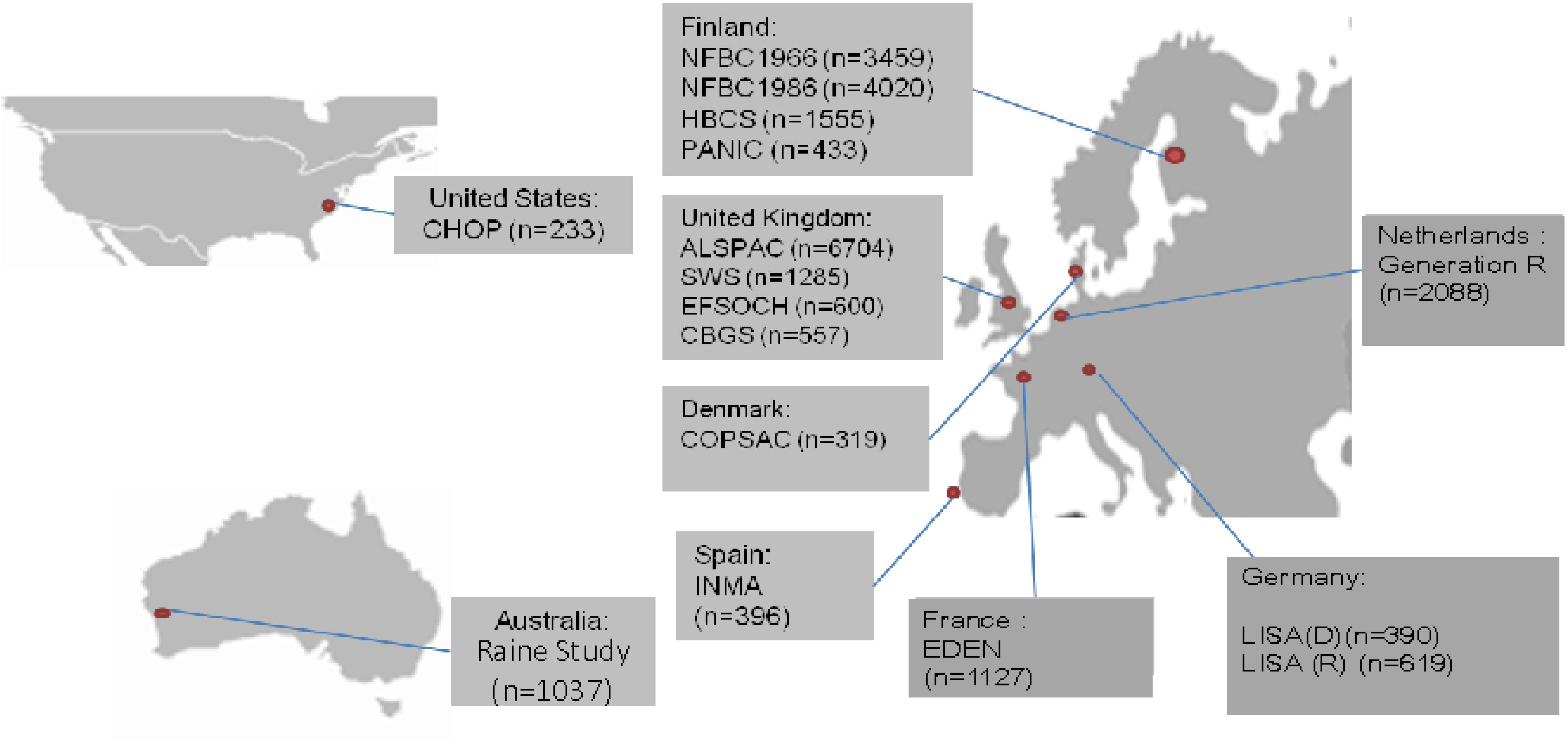

Fig. S3. The participating studies with their geographical location. Avon Longitudinal Study of Parents and Children [ALSPAC], Cambridge Baby Growth Study [CBGS], Children's Hospital of Philadelphia [CHOP], COpenhagen Study on Asthma in Children [COPSAC], Danish National Birth Cohort [DNBC], Étude des Déterminants pré et postnatals du développement et de la santé de l'ENfant [EDEN], Helsinki Birth Cohort Study [HBCS], INfancia y Medio Ambiente [Environment and Childhood] Project [INMA], Lifestyle – Immune System – Allergy Study [LISA D, R],The Exeter Family Study of Childhood Health [EFSOCH], Northern Finland Birth Cohort Study 1966 [NFBC1966], Northern Finland Birth Cohort Study 1986 [NFBC1986], The Generation R Study, The Physical Activity and Nutrition in Children [PANIC] Study, The Southampton Women's Survey [SWS], The Western Australian Pregnancy Cohort [Raine] Study. Early Growth Genetics Consortium [EGG].

A. Peak height velocity (PHV)

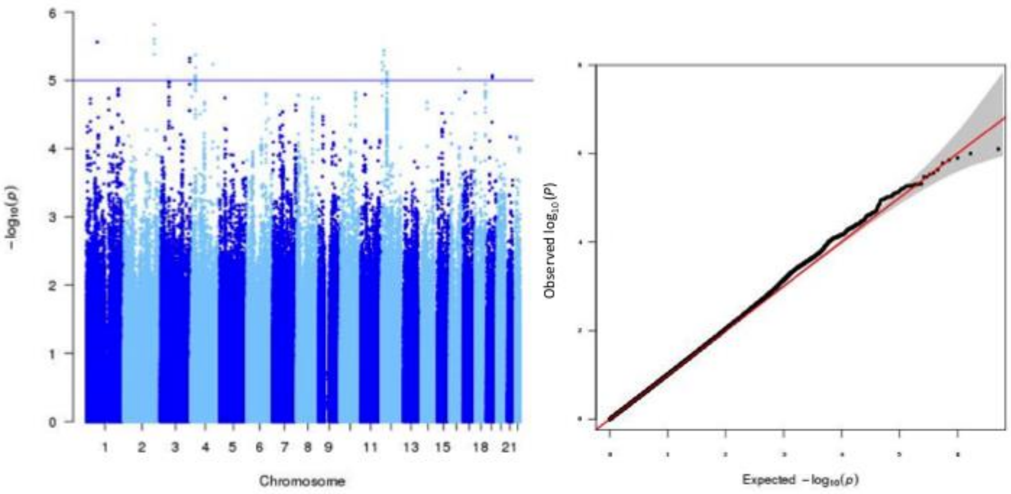

B. Peak weight velocity (PWV)

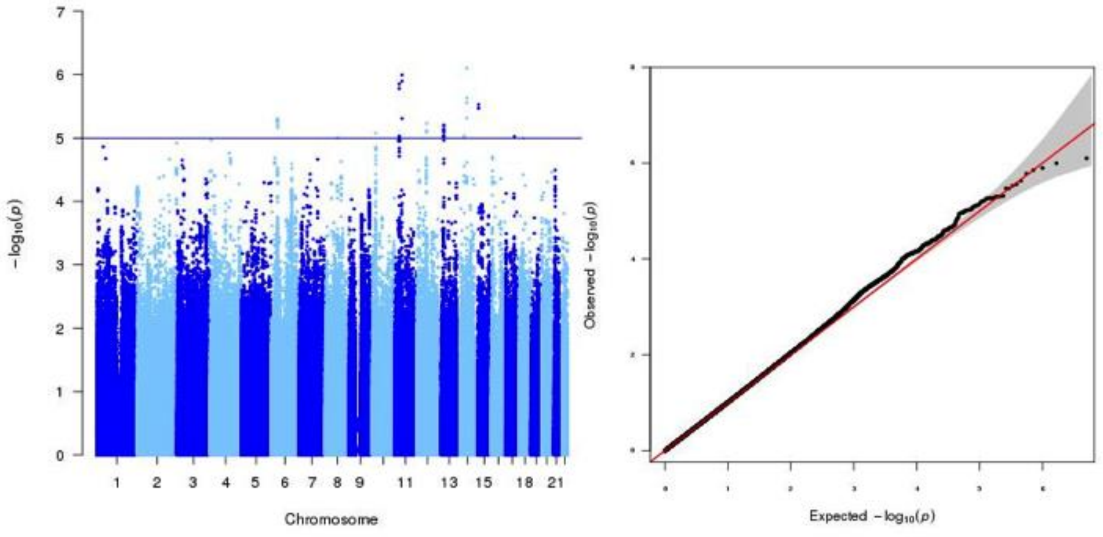

C. Age at adiposity peak (AGE-AP)

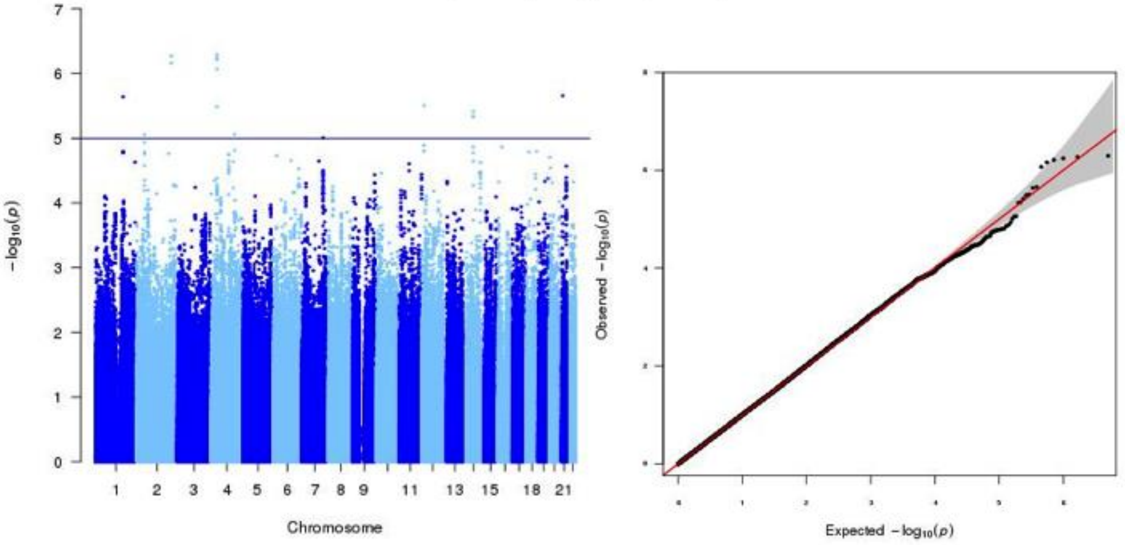

D. BMI at adiposity peak (BMI-AP)

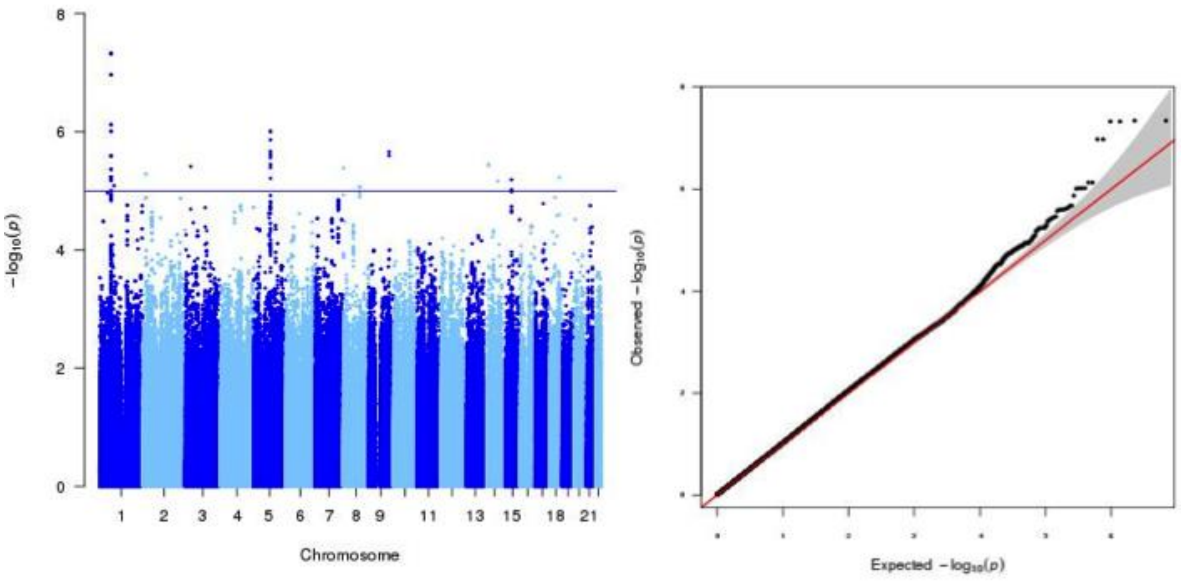

E. Age at adiposity rebound (AGE-AR)

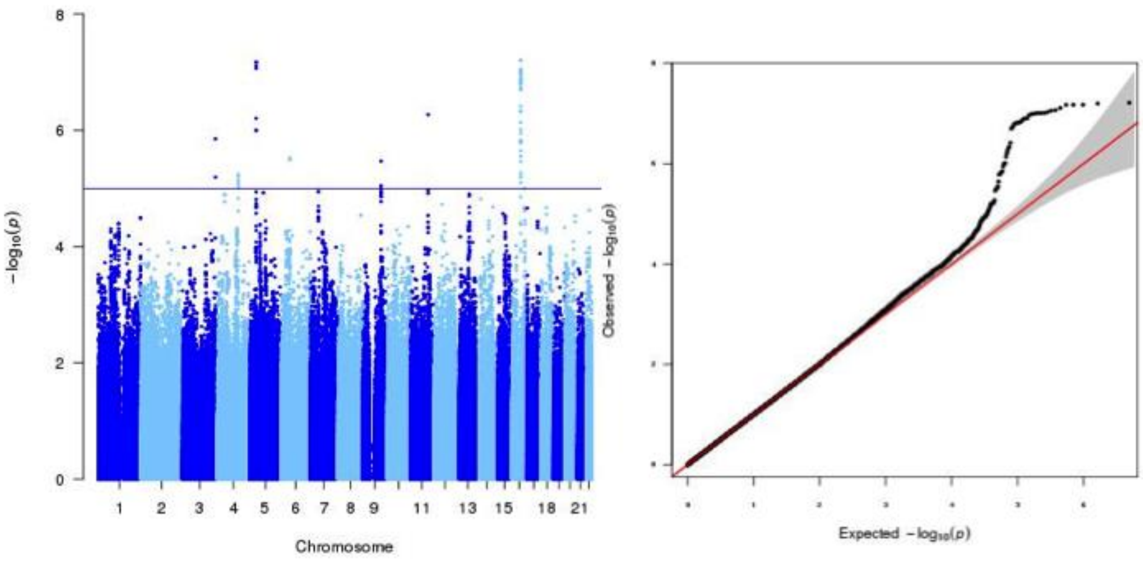

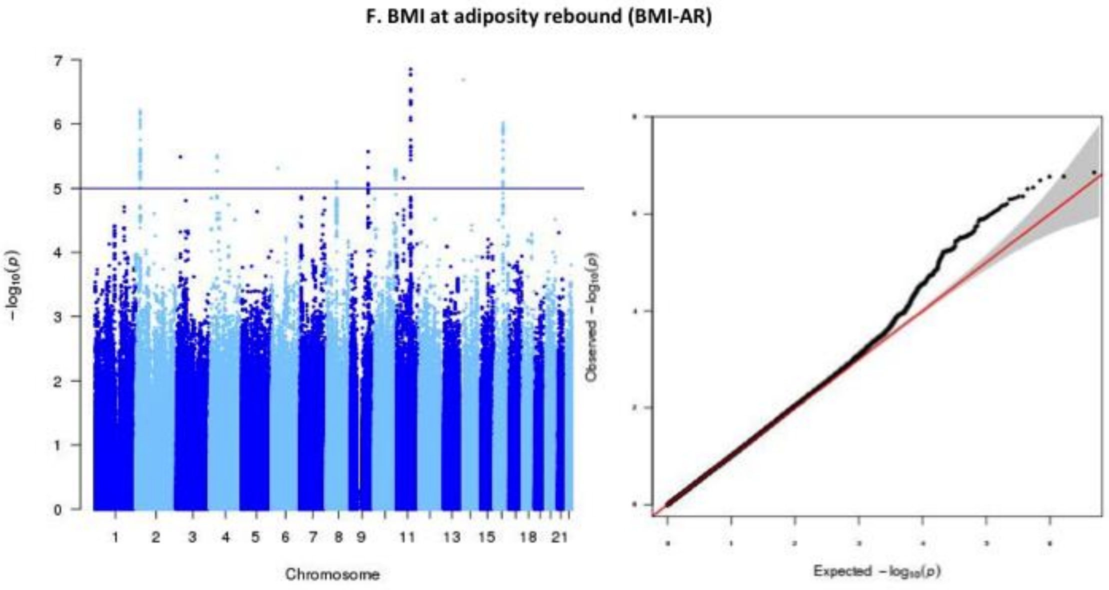

Fig. S4. The Manhattan plot and quantile-quantile plot of the association *P* values for the six early growth phenotypes from stage 1 genome-wide association analyses. In the Manhattan plot, the -log10 of the *P*-value for each of SNPs is plotted against the genomic position [NCBI Build 36]. In the QQ-plot of 2.5 million SNPs from the meta-analysis in up to N=7,215 discovery samples the black dots represent observed *P*-values and the red lines represent expected *P*-values under the null distribution.

A)

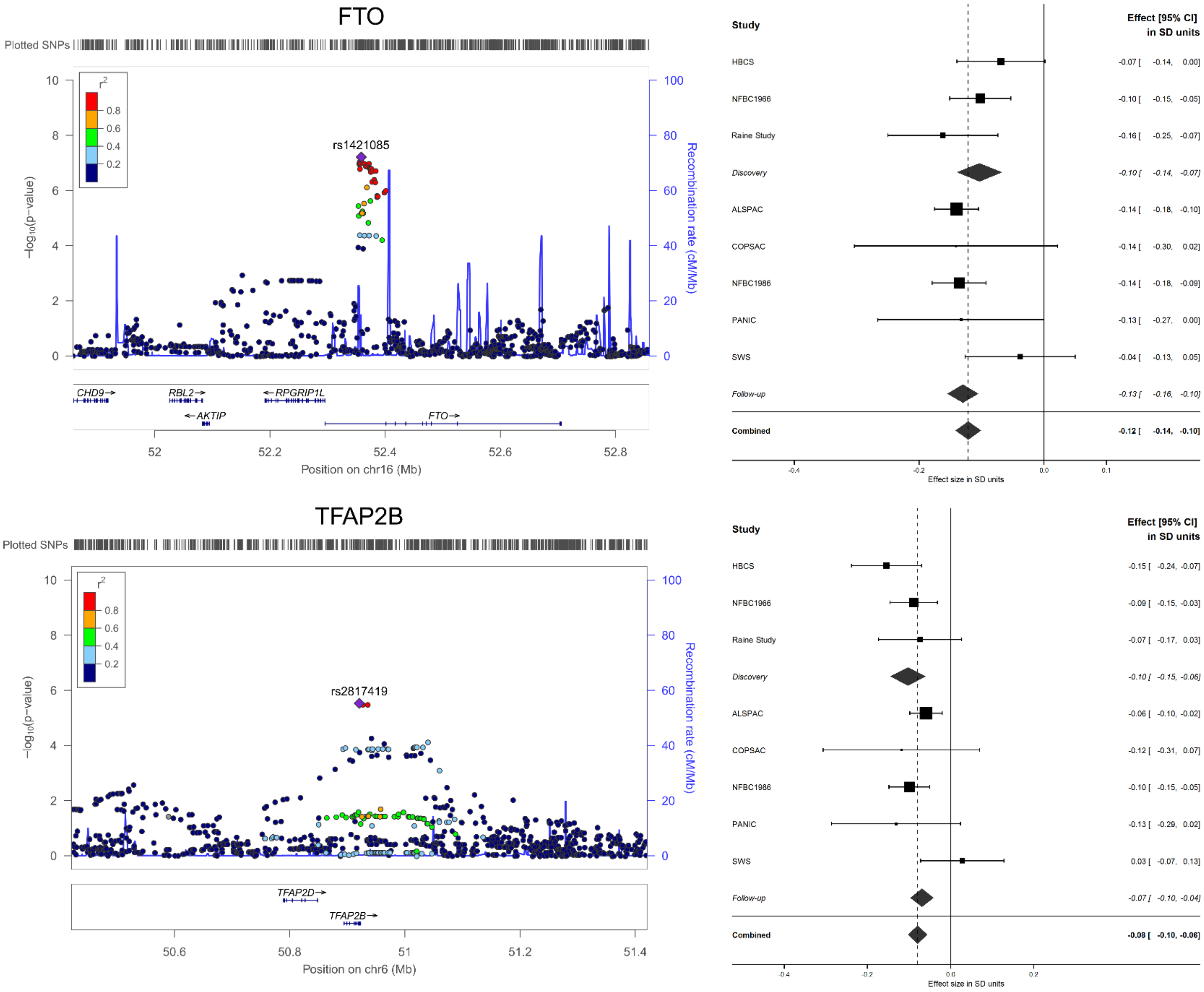
FTO
Plotted SNPs
rs1421085
$-\log_{10}$(p-value)
Recombination rate (cM/Mb)
r2
0.8
0.6
0.4
0.2
CHD9
RBL2
RPGRIP1L
AKTIP
FTO
Position on chr16 (Mb)
Study
Effect [95% CI] in SD units
HBCS -0.07 [-0.14, 0.00]
NFBC1966 -0.10 [-0.15, -0.05]
Raine Study -0.16 [-0.25, -0.07]
Discovery -0.10 [-0.14, -0.07]
ALSPAC -0.14 [-0.18, -0.10]
COPSAC -0.14 [-0.30, 0.02]
NFBC1986 -0.14 [-0.18, -0.09]
PANIC -0.13 [-0.27, 0.00]
SWS -0.04 [-0.13, 0.05]
Follow-up -0.13 [-0.16, -0.10]
Combined -0.12 [-0.14, -0.10]
Effect size in SD units
TFAP2B
Plotted SNPs
rs2817419
$-\log_{10}$(p-value)
Recombination rate (cM/Mb)
TFAP2D
TFAP2B
Position on chr6 (Mb)
Study
Effect [95% CI] in SD units
HBCS -0.15 [-0.24, -0.07]
NFBC1966 -0.09 [-0.15, -0.03]
Raine Study -0.07 [-0.17, 0.03]
Discovery -0.10 [-0.15, -0.06]
ALSPAC -0.06 [-0.10, -0.02]
COPSAC -0.12 [-0.31, 0.07]
NFBC1986 -0.10 [-0.15, -0.05]
PANIC -0.13 [-0.29, 0.02]
SWS 0.03 [-0.07, 0.13]
Follow-up -0.07 [-0.10, -0.04]
Combined -0.08 [-0.10, -0.06]
Effect size in SD units

B)

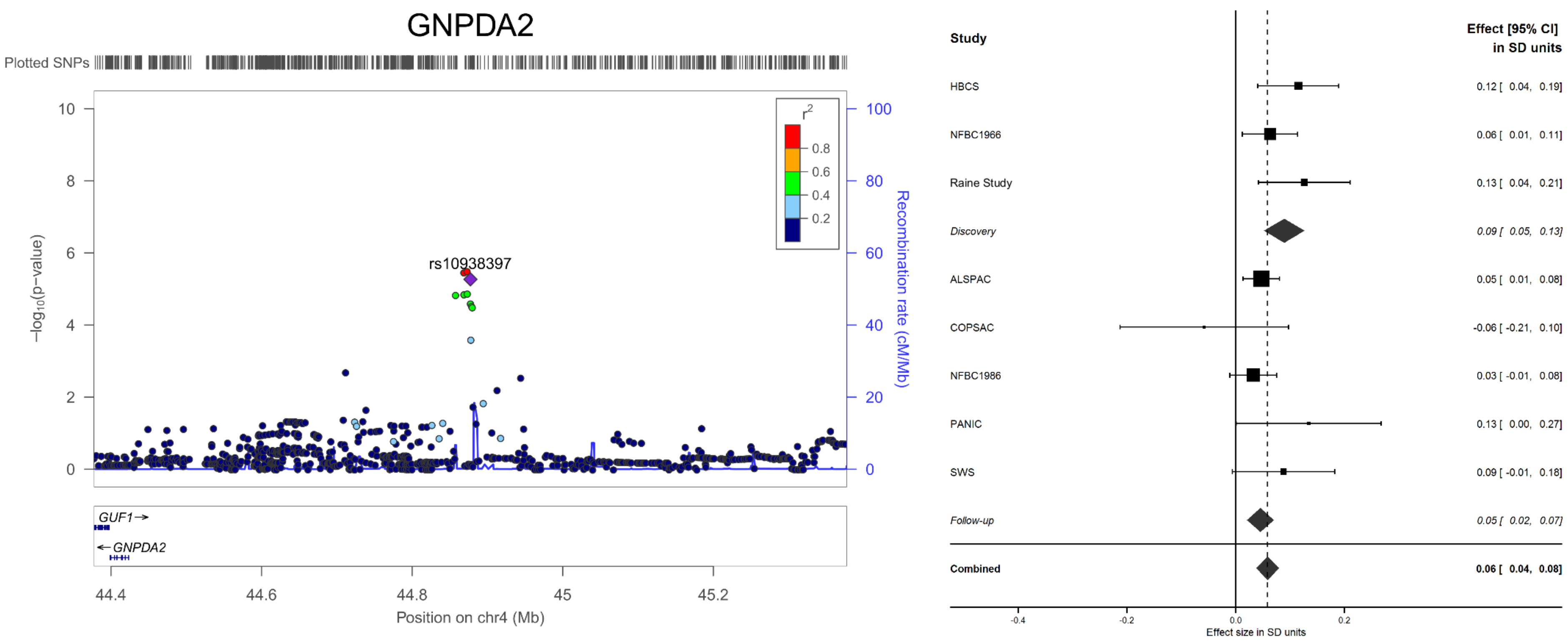

Fig. S5. Regional association and forest plots of the three genome-wide significant loci associated with early growth traits that have been previously linked with adult BMI. A) AGE at AR and **B)** BMI at AR. Purple diamond indicates the most significantly associated SNP in stage 1 meta-analysis, and circles represent the other SNPs in the region with coloring from blue to red corresponding to r^2 values from 0 to 1 with the index SNP. The SNP position refers to the NCBI build 36. Estimated recombination rates are from HapMap build 36. Forest plots from the meta-analysis for each of the identified loci are plotted abreast. Effect size [95% CI] in each individual study, discovery, follow-up and combined meta-analysis stages are presented from fixed effects models.

a)

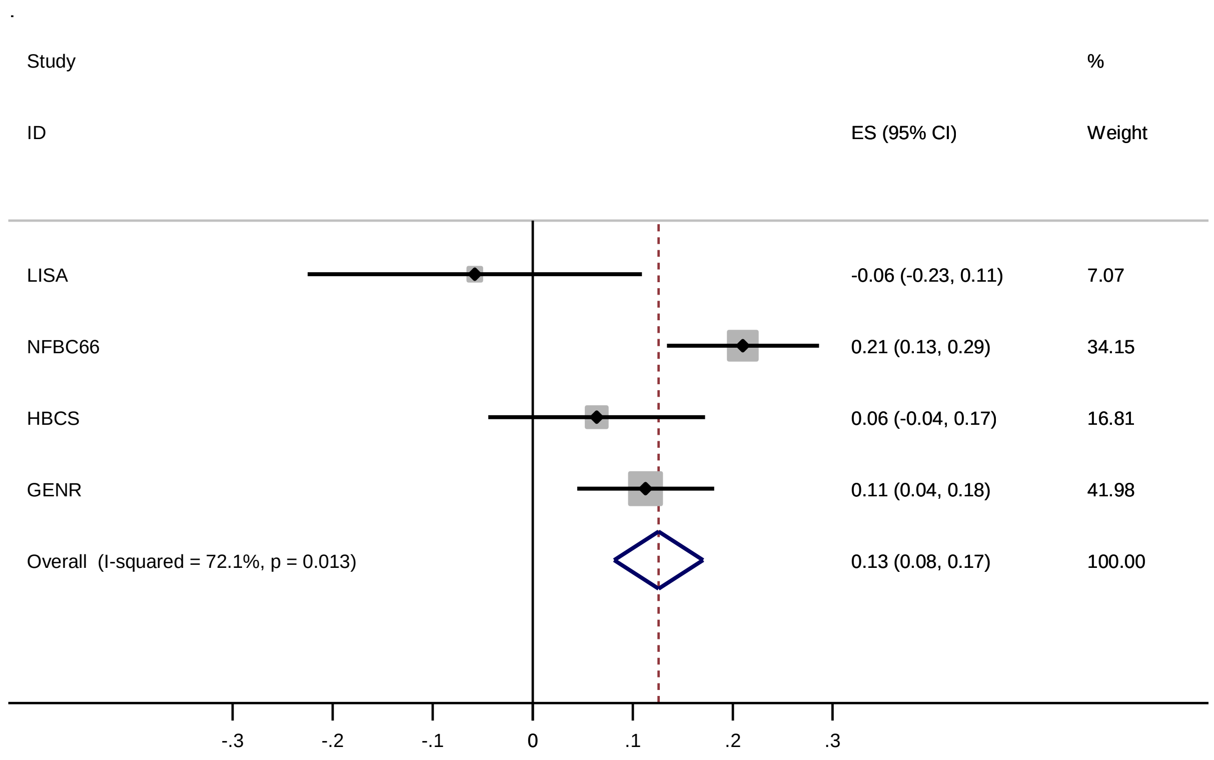

Study
ID
%
ES (95% CI)
Weight
LISA
-0.06 (-0.23, 0.11)
7.07
NFBC66
0.21 (0.13, 0.29)
34.15
HBCS
0.06 (-0.04, 0.17)
16.81
GENR
0.11 (0.04, 0.18)
41.98
Overall (I-squared = 72.1%, p = 0.013)
0.13 (0.08, 0.17)
100.00
-.3
-.2
-.1
0
.1
.2
.3

b)

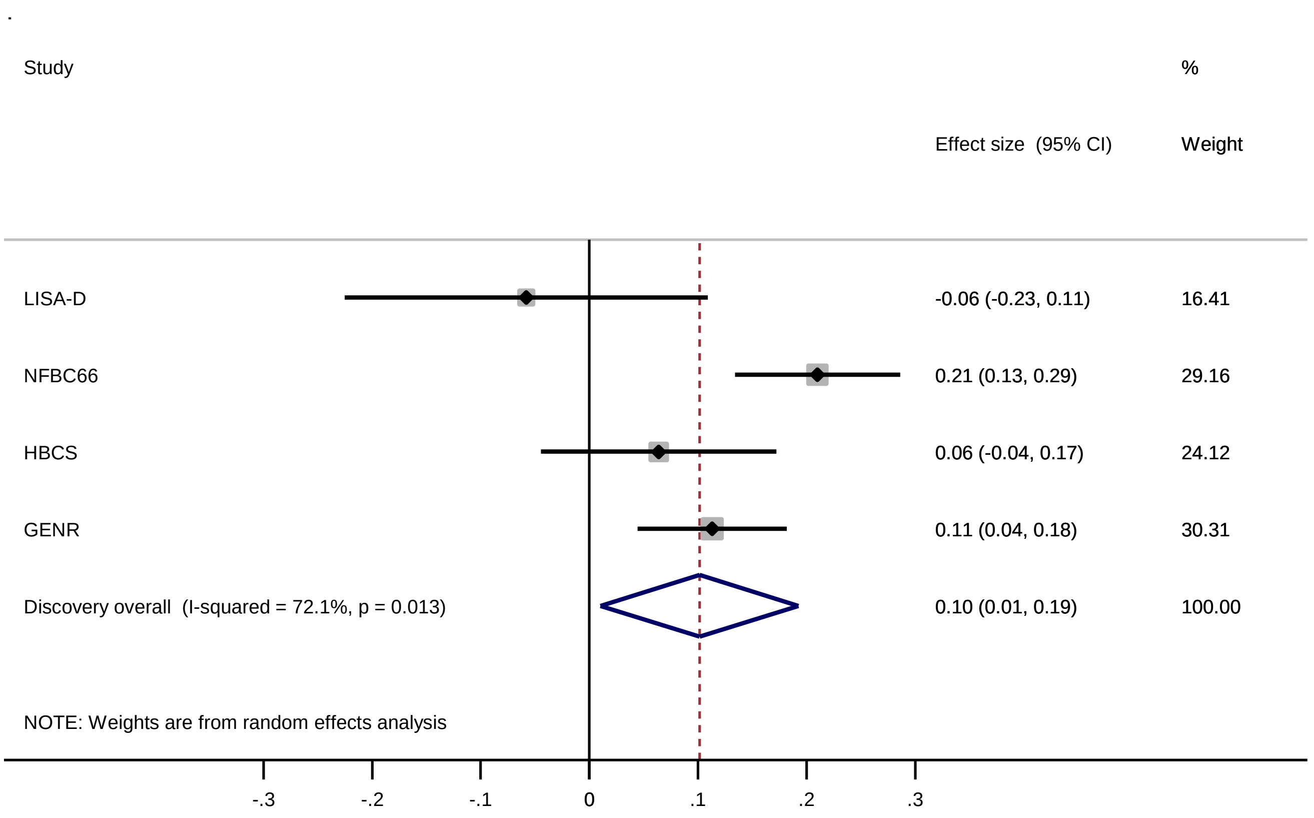
Study
%
Effect size (95% CI)
Weight
LISA-D
-0.06 (-0.23, 0.11)
16.41
NFBC66
0.21 (0.13, 0.29)
29.16
HBCS
0.06 (-0.04, 0.17)
24.12
GENR
0.11 (0.04, 0.18)
30.31
Discovery overall (I-squared = 72.1%, p = 0.013)
0.10 (0.01, 0.19)
100.00
NOTE: Weights are from random effects analysis
-.3
-.2
-.1
0
.1
.2
.3

c)

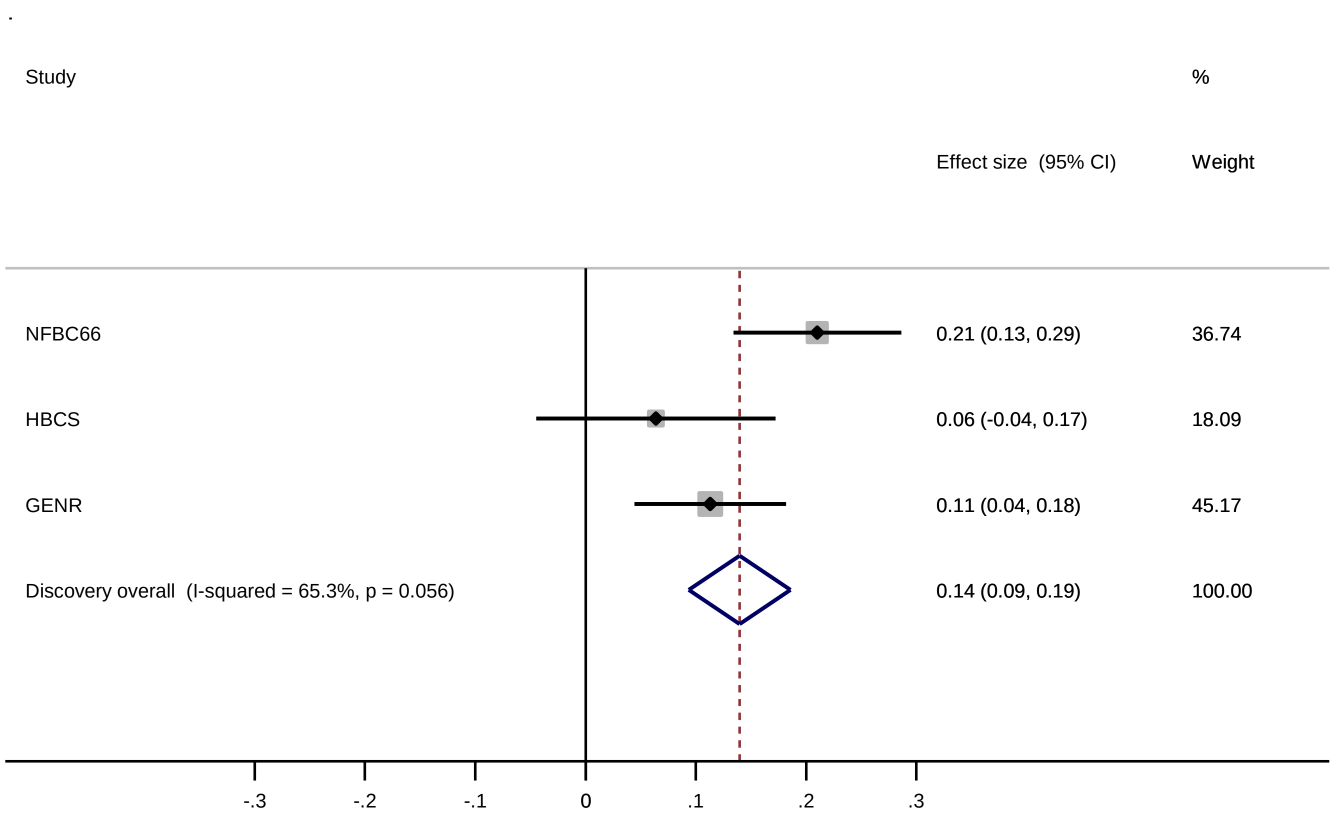
.
Study
%
Effect size (95% CI)
Weight
NFBC66
0.21 (0.13, 0.29)
36.74
HBCS
0.06 (-0.04, 0.17)
18.09
GENR
0.11 (0.04, 0.18)
45.17
Discovery overall (I-squared = 65.3%, p = 0.056)
0.14 (0.09, 0.19)
100.00
-.3
-.2
-.1
0
.1
.2
.3

d)

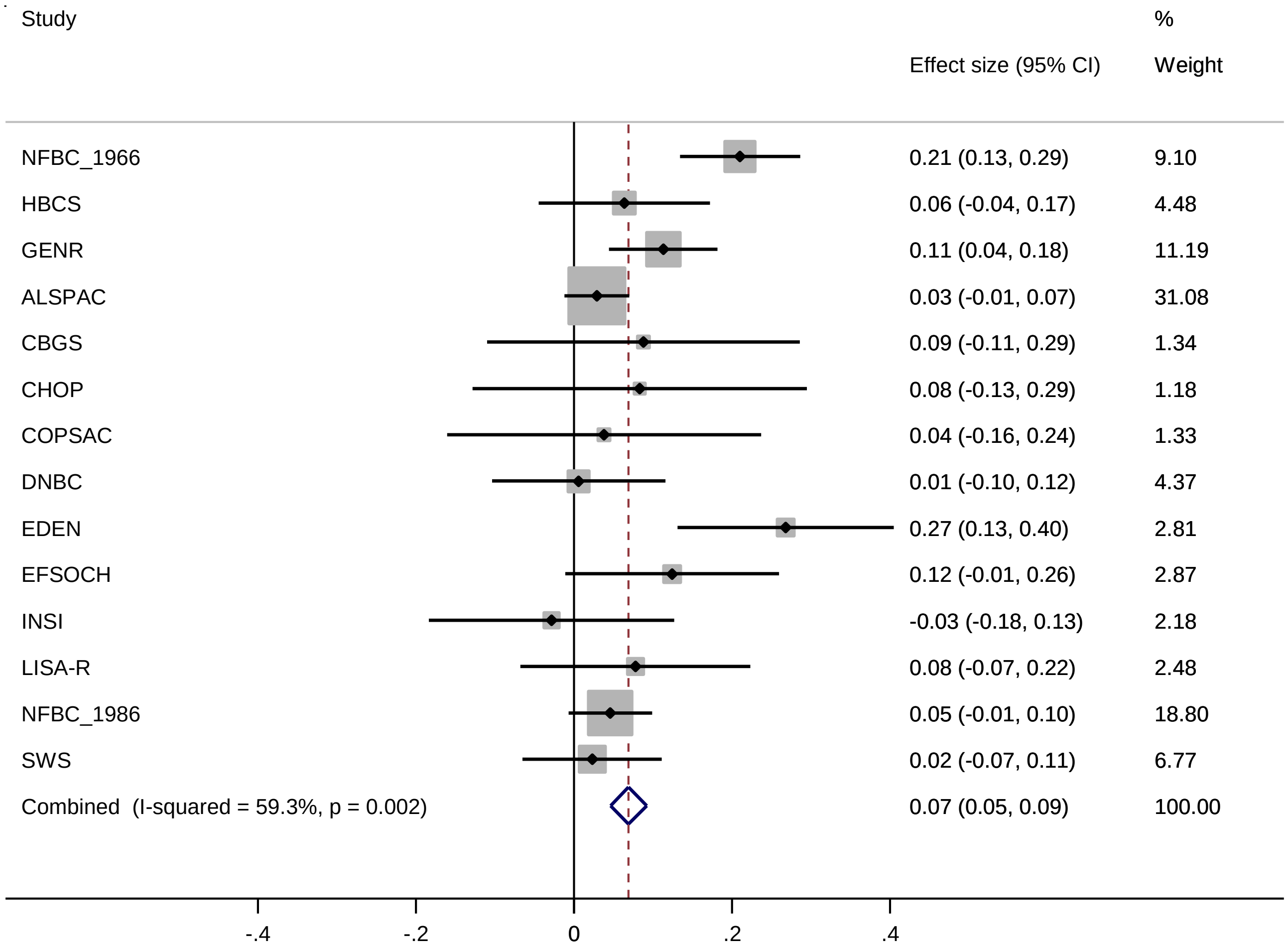
Study
%
Effect size (95% CI)
Weight
NFBC_1966
0.21 (0.13, 0.29)
9.10
HBCS
0.06 (-0.04, 0.17)
4.48
GENR
0.11 (0.04, 0.18)
11.19
ALSPAC
0.03 (-0.01, 0.07)
31.08
CBGS
0.09 (-0.11, 0.29)
1.34
CHOP
0.08 (-0.13, 0.29)
1.18
COPSAC
0.04 (-0.16, 0.24)
1.33
DNBC
0.01 (-0.10, 0.12)
4.37
EDEN
0.27 (0.13, 0.40)
2.81
EFSOCH
0.12 (-0.01, 0.26)
2.87
INSI
-0.03 (-0.18, 0.13)
2.18
LISA-R
0.08 (-0.07, 0.22)
2.48
NFBC_1986
0.05 (-0.01, 0.10)
18.80
SWS
0.02 (-0.07, 0.11)
6.77
Combined (I-squared = 59.3%, p = 0.002)
0.07 (0.05, 0.09)
100.00
-.4
-.2
0
.2
.4

e)

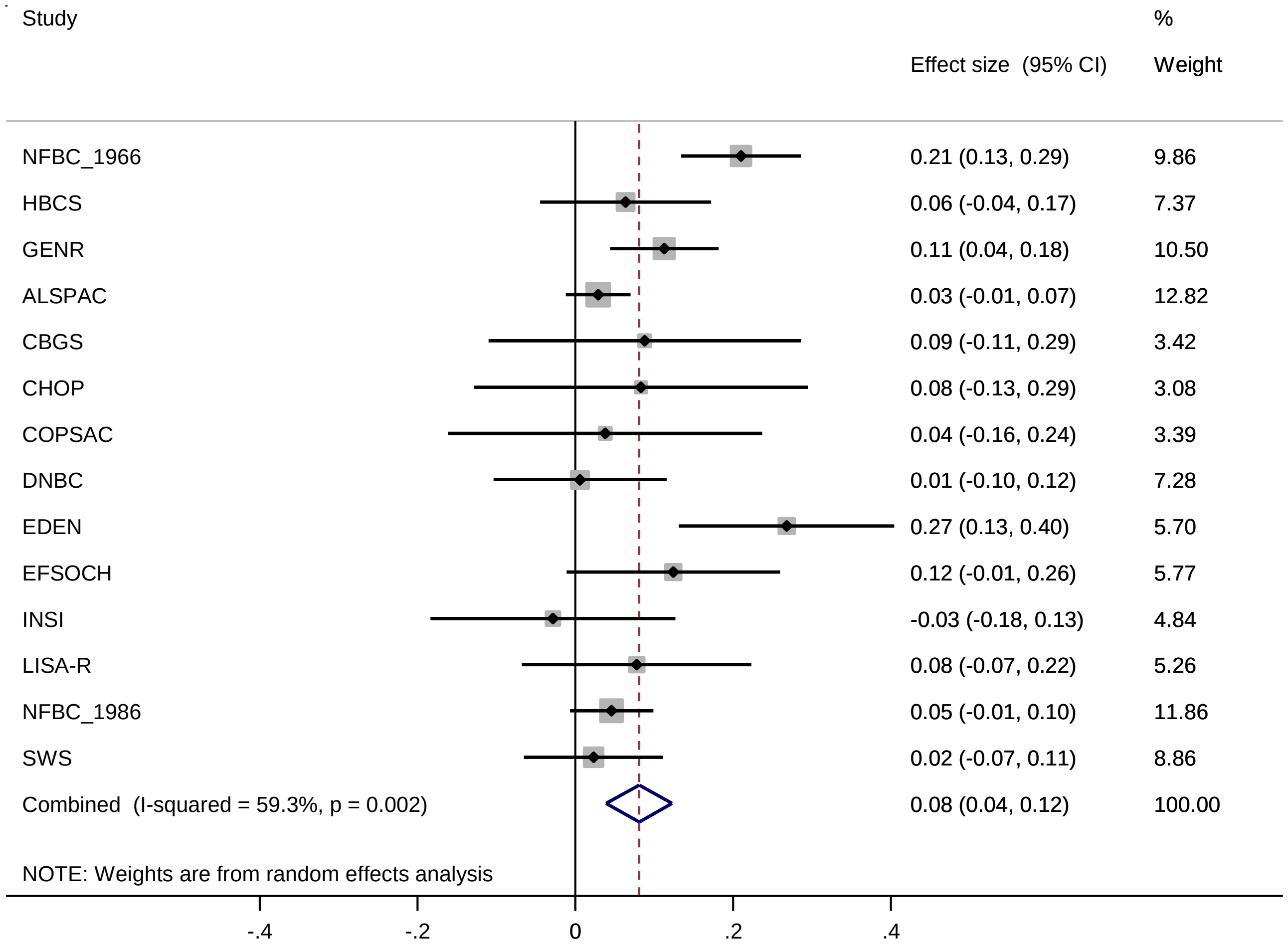
Study
%
Effect size (95% CI)
Weight
NFBC_1966
0.21 (0.13, 0.29)
9.86
HBCS
0.06 (-0.04, 0.17)
7.37
GENR
0.11 (0.04, 0.18)
10.50
ALSPAC
0.03 (-0.01, 0.07)
12.82
CBGS
0.09 (-0.11, 0.29)
3.42
CHOP
0.08 (-0.13, 0.29)
3.08
COPSAC
0.04 (-0.16, 0.24)
3.39
DNBC
0.01 (-0.10, 0.12)
7.28
EDEN
0.27 (0.13, 0.40)
5.70
EFSOCH
0.12 (-0.01, 0.26)
5.77
INSI
-0.03 (-0.18, 0.13)
4.84
LISA-R
0.08 (-0.07, 0.22)
5.26
NFBC_1986
0.05 (-0.01, 0.10)
11.86
SWS
0.02 (-0.07, 0.11)
8.86
Combined (I-squared = 59.3%, p = 0.002)
0.08 (0.04, 0.12)
100.00
NOTE: Weights are from random effects analysis
-.4
-.2
0
.2
.4

f)

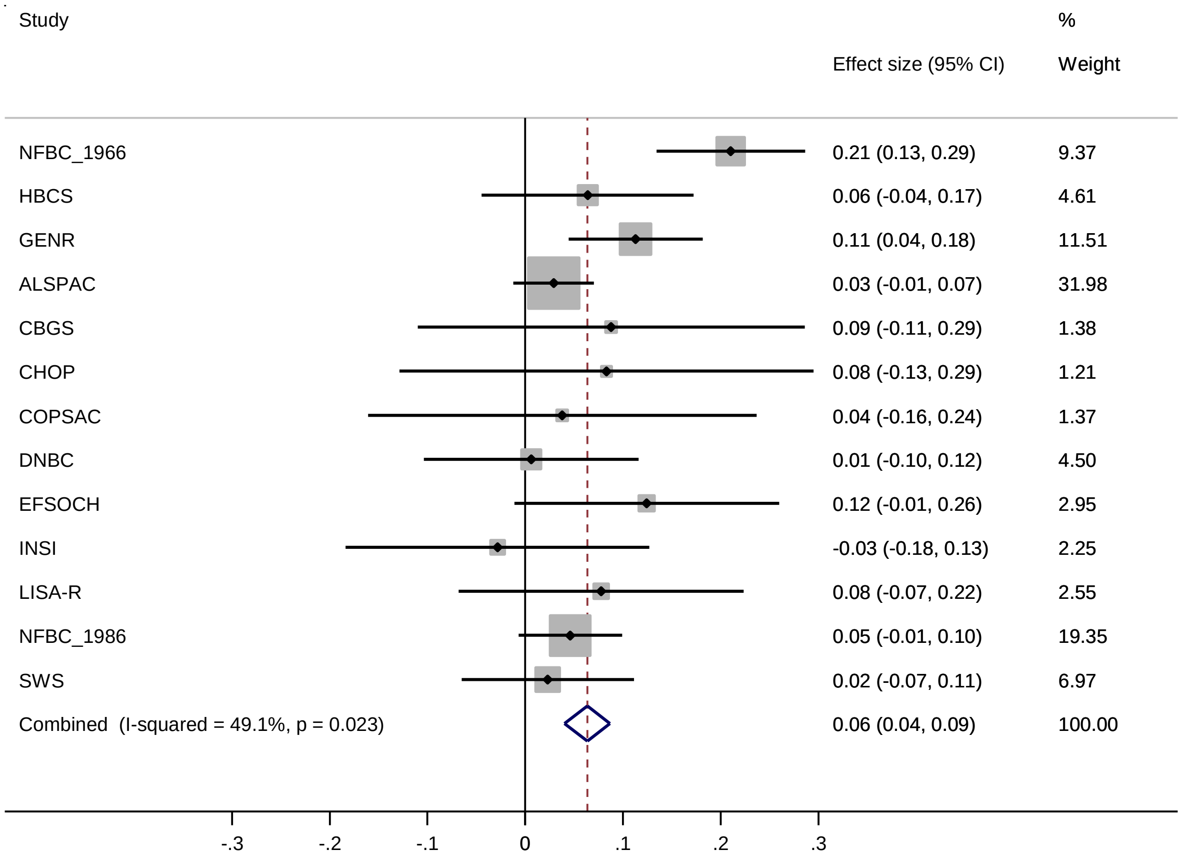

Study
%
Effect size (95% CI)
Weight
NFBC_1966
0.21 (0.13, 0.29)
9.37
HBCS
0.06 (-0.04, 0.17)
4.61
GENR
0.11 (0.04, 0.18)
11.51
ALSPAC
0.03 (-0.01, 0.07)
31.98
CBGS
0.09 (-0.11, 0.29)
1.38
CHOP
0.08 (-0.13, 0.29)
1.21
COPSAC
0.04 (-0.16, 0.24)
1.37
DNBC
0.01 (-0.10, 0.12)
4.50
EFSOCH
0.12 (-0.01, 0.26)
2.95
INSI
-0.03 (-0.18, 0.13)
2.25
LISA-R
0.08 (-0.07, 0.22)
2.55
NFBC_1986
0.05 (-0.01, 0.10)
19.35
SWS
0.02 (-0.07, 0.11)
6.97
Combined (I-squared = 49.1%, p = 0.023)
0.06 (0.04, 0.09)
100.00
-.3
-.2
-.1
0
.1
.2
.3

g)

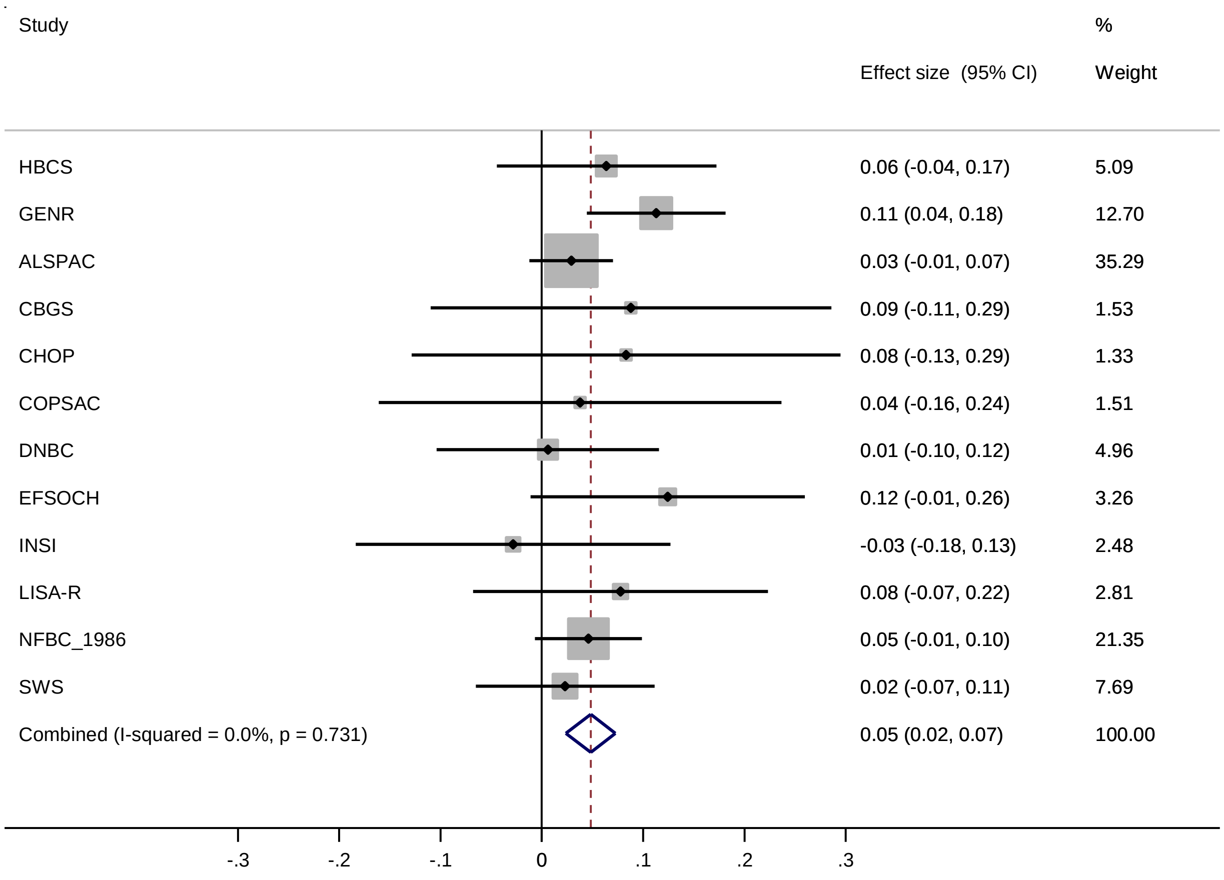
Study
%
Effect size (95% CI)
Weight
HBCS
0.06 (-0.04, 0.17)
5.09
GENR
0.11 (0.04, 0.18)
12.70
ALSPAC
0.03 (-0.01, 0.07)
35.29
CBGS
0.09 (-0.11, 0.29)
1.53
CHOP
0.08 (-0.13, 0.29)
1.33
COPSAC
0.04 (-0.16, 0.24)
1.51
DNBC
0.01 (-0.10, 0.12)
4.96
EFSOCH
0.12 (-0.01, 0.26)
3.26
INSI
-0.03 (-0.18, 0.13)
2.48
LISA-R
0.08 (-0.07, 0.22)
2.81
NFBC_1986
0.05 (-0.01, 0.10)
21.35
SWS
0.02 (-0.07, 0.11)
7.69
Combined (I-squared = 0.0%, p = 0.731)
0.05 (0.02, 0.07)
100.00
-.3
-.2
-.1
0
.1
.2
.3

Fig. S6. Heterogeneity analyses of the GWAS lead SNP rs9436303 at *LEPR/LEPROT* locus. a) Discovery stage fixed effect inverse variance meta-analysis of rs9436303 using all four studies. There was heterogeneity between the discovery stage studies (I^2=72.1%, *P*=0.01). b) Discovery stage random effect inverse variance meta-analysis of rs9436303 using all four studies. Heterogeneity was still present between studies (I^2=72.1%, *P*=0.013) and the random effect meta-analysis did not change the point estimates. c) Discovery stage fixed effect meta-analysis excluding the directionally inconsistent LISA-D study. Heterogeneity between discovery stage studies was reduced by removing the LISA-D study (I^2=65.3%, *P*=0.06) but the point estimates were unchanged with or without the LISA-D study. d) Fixed effect meta-analysis of rs9436303 using discovery and replication studies. There was moderate heterogeneity between the studies (I^2=59.3 %, *P*=0.002). e) Random effect meta-analysis of rs9436303 using discovery and replication studies. Moderate heterogeneity between the studies was still present I^2=59.3 %, *P*=0.002) and the point estimates did not change. f) Fixed effect meta-analysis of rs9436303 in discovery and replication studies excluding the EDEN study which showed inflated results from the point estimates on the forest plots. Removing the EDEN study reduced the heterogeneity but still some heterogeneity was present between the studies (I^2=49.1%, *P*=0.02) but the overall point estimate was essentially the same. g) Fixed effect meta-analysis of rs9436303 in discovery and replication studies by further excluding the NFBC1966 study, which also showed inflated, results from the point estimates. Removing both EDEN and NFBC1966 from meta-analysis showed no heterogeneity between studies (I^2=0.0%, *P*=0.73) and the overall point estimate was essentially the same.

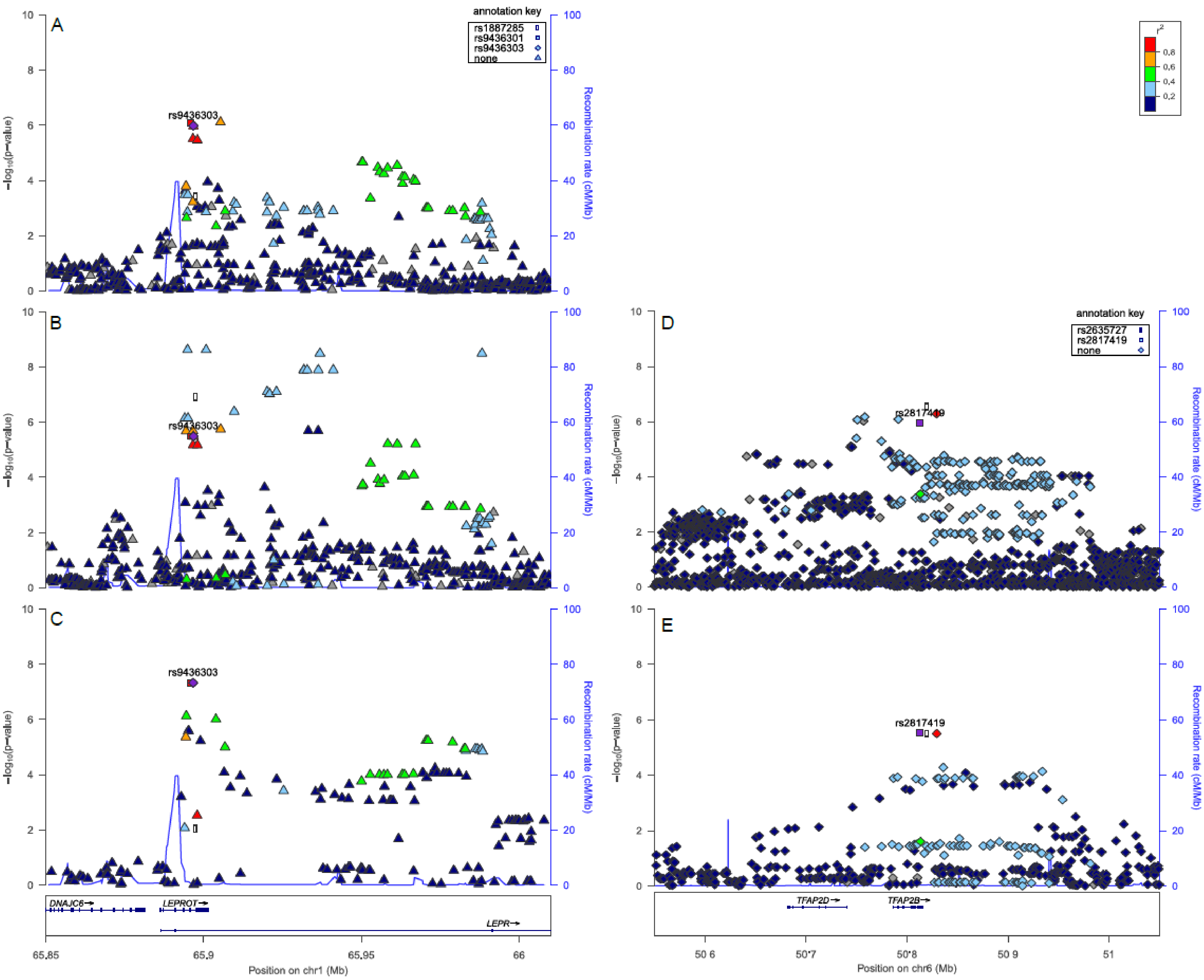
A
B
C
D
E
annotation key
rs1887285
rs9436301
rs9436303
none
rs2635727
rs2817419
rs9436303
rs2817419
r2
0,8
0,6
0,4
0,2
−log10(p−value)
Recombination rate (cM/Mb)
DNAJC6
LEPROT
LEPR
TFAP2D
TFAP2B
65,85
65,9
65,95
66
Position on chr1 (Mb)
50 6
50 7
50 8
50 9
51
Position on chr6 (Mb)

Fig. S7. Regional plots of the GWAS and GTEx cis-eQTL data used in the colocalization analysis of the early growth–associated loci. The y-axis shows the –log10 GWAS p-values and –log10 eQTL p-values of A) *LEPROT* in thyroid tissue (n=399), B) *LEPR* in EBV-transformed lymphocytes (n=117) C) BMI-AP (n=6,219), D) TFPAB2 in testis (n=255), E) Age-AR (n=6,051). The x-axis shows the physical position on the chromosome in hg19 build. Gene annotations from UCSC Genome Browser. –log10 GWAS p-values in the y axis are from stage 1 GWAS analyses. All eQTL association p-values obtained from the Genotype-Tissue Expression (GTEx) project.

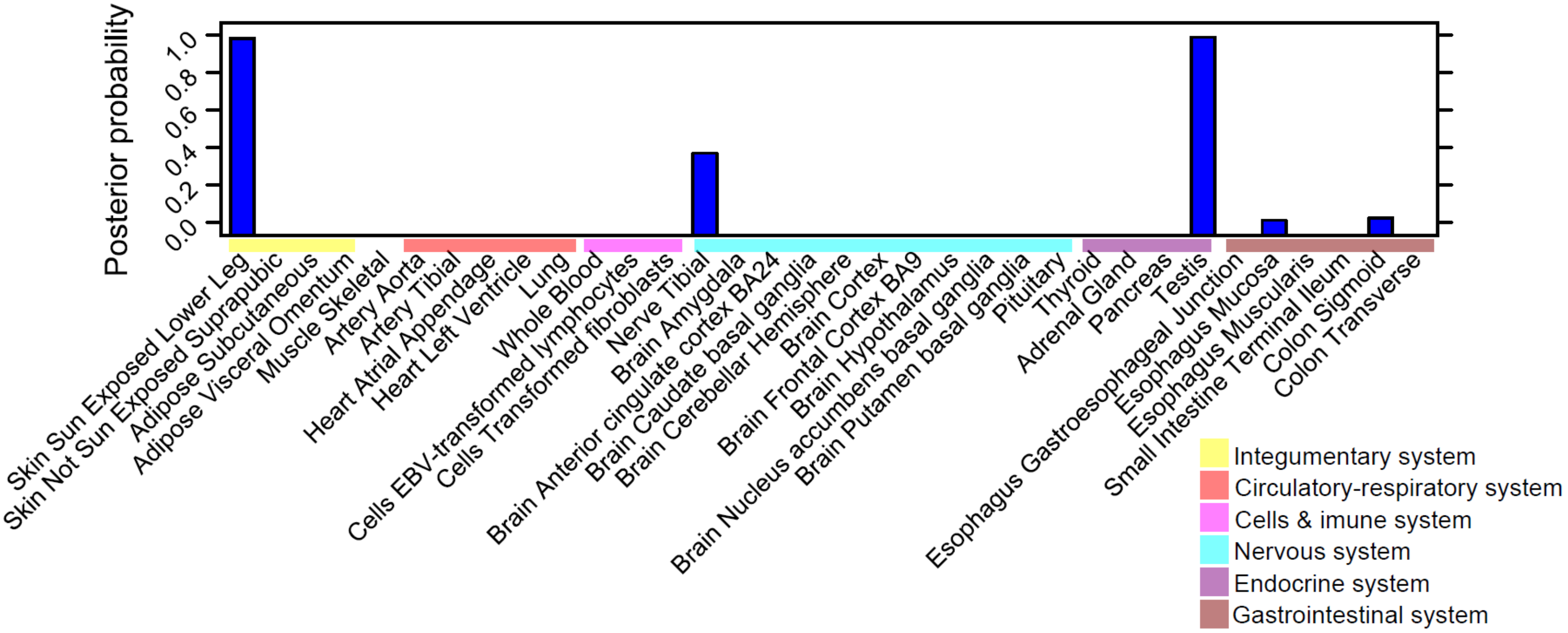

Fig. S8. Tissue-specific PPs of colocalization of *TFAP2B*. *TAFP2B* eQTL in Skin and Testis colocalized (P>0.95) with Age-AR variant rs2817419. Posterior probability of eQTL and GWAS SNP sharing a causal variant regulating the gene expression levels of *TFAP2B*. Colocalization reported for GTEX eQTLs data in 34 tissues that express at least one of the genes.

Query SNP: rs9436303 and variants with r^2 >= 0.8

A

chr	pos (hg38)	LD (r^2)	LD (D')	variant	Ref	Alt	AFR freq	AMR freq	ASN freq	EUR freq	SiPhy cons	Promoter histone marks	Enhancer histone marks	DNAse	Proteins bound	Motifs changed	NHGRI/EBI GWAS hits	GRASP QTL hits	Selected eQTL hits	GENCODE genes	dbSNP func annot
1	65430244	0.99	1	rs9436301	T	C	0.35	0.23	0.12	0.21		5 tissues	11 tissues	MUS,VAS		HDAC2		1 hit	6 hits	LEPROT	intronic
1	65430991	1	1	rs9436303	A	G	0.35	0.23	0.12	0.21			13 tissues	SKIN,VAS		HNF4,Zbtb3			5 hits	LEPROT	intronic
1	65431004	1	1	rs9436304	G	C	0.35	0.23	0.12	0.21			13 tissues	SKIN,VAS					2 hits	LEPROT	intronic

Query SNP: rs2817419 and variants with r^2 >= 0.8

B

chr	pos (hg38)	LD (r^2)	LD (D')	variant	Ref	Alt	AFR freq	AMR freq	ASN freq	EUR freq	SiPhy cons	Promoter histone marks	Enhancer histone marks	DNAse	Proteins bound	Motifs changed	NHGRI/EBI GWAS hits	GRASP QTL hits	Selected eQTL hits	GENCODE genes	dbSNP func annot
6	50845193	1	1	rs2817419	G	A	0.69	0.78	0.80	0.75		ESC, IPSC	5 tissues	ESC		Evi-1,Rad21				TFAP2B	3'-UTR
6	50853227	0.93	0.99	rs2635727	T	C	0.72	0.79	0.80	0.76			ESDR			Irx,Zfp187		1 hit		5.6kb 3' of TFAP2B	
6	50861758	0.91	0.98	rs4715208	A	G	0.52	0.78	0.79	0.76			ESDR			BCL				14kb 3' of TFAP2B	

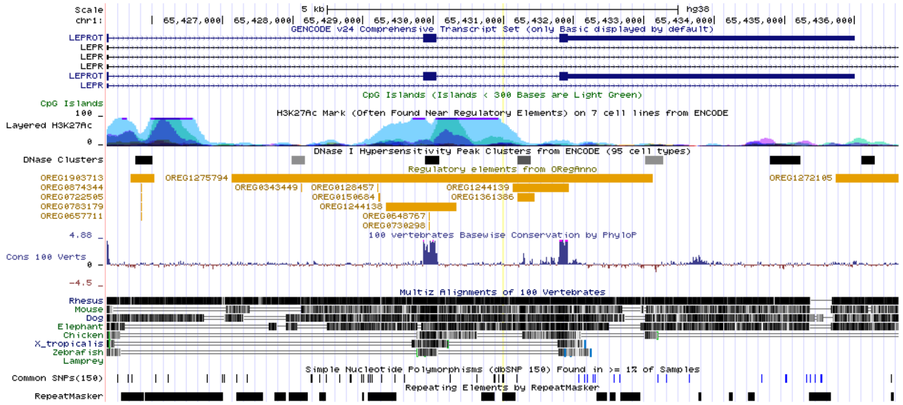

C

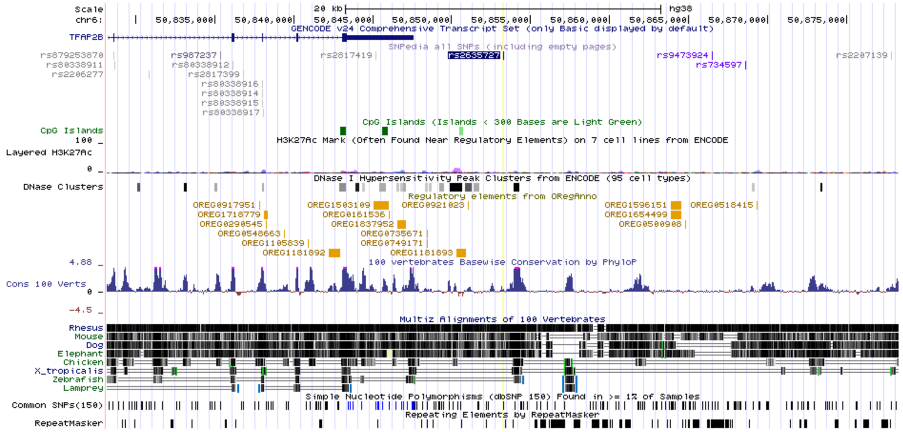

D

Fig. S9. Genomic annotation analysis of the colocalized variants involved in the regulation of LEPR, LEPROT, and TFAP2B gene expression. HaploReg analyses show that both A) rs9436303 and B) rs2817419 overlap regions of regulatory elements, open chromatin, predicted

enhancers, and eQTLs, which we further investigate in the Genome Browser. Genome Browser visualization shows regions of regulatory elements and histone marks overlapping C) rs9436303 (*LEPR/LEPROT*) and D) rs2817419 (*TFAP2B*) loci.

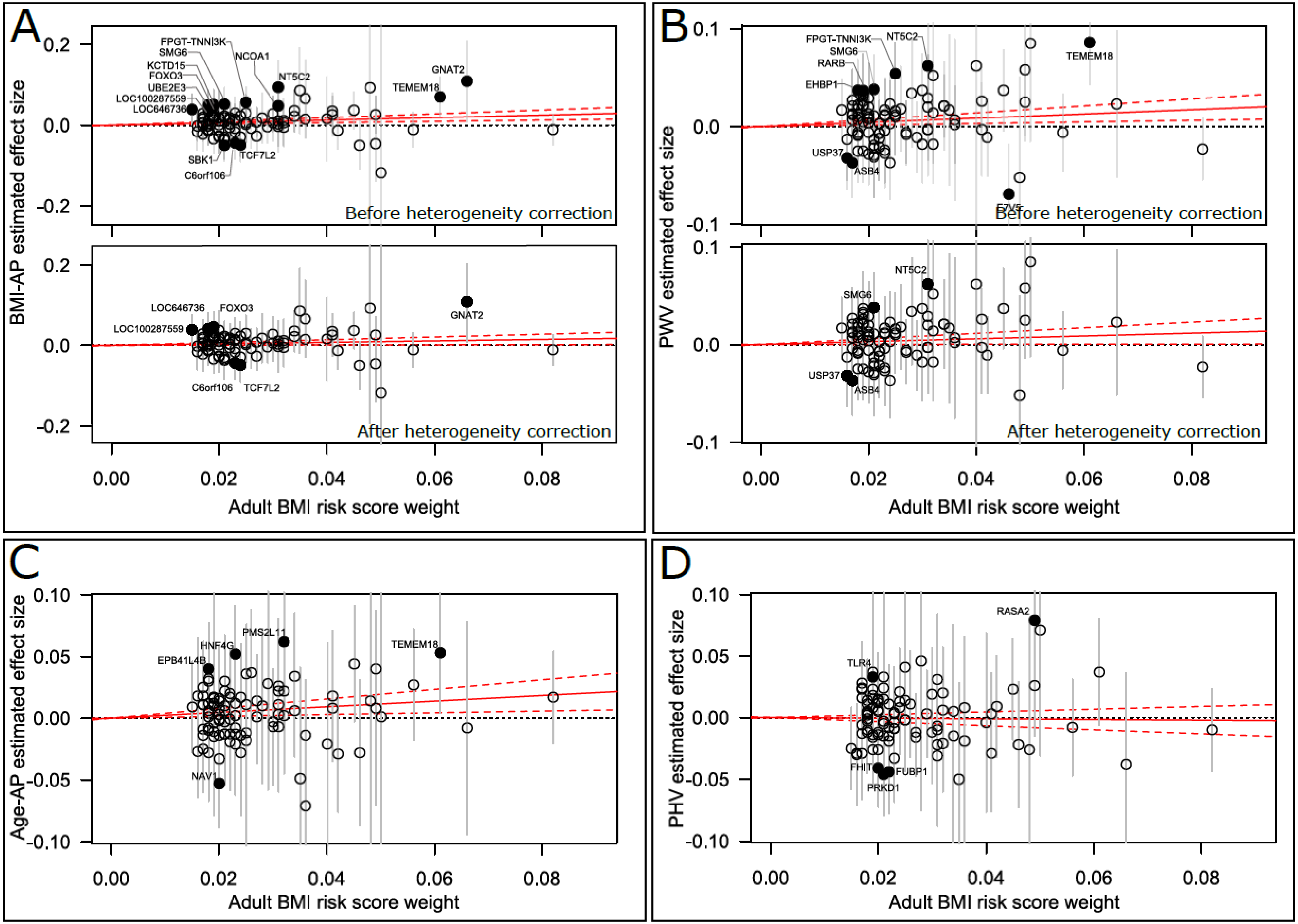

A
BMI-AP estimated effect size
FPGT-TNNI3K
SMG6
KCTD15
FOXO3
UBE2E3
LOC100287559
LOC646736
NCOA1
NT5C2
GNAT2
TEMEM18
SBK1
C6orf106
TCF7L2
Before heterogeneity correction
LOC646736
FOXO3
LOC100287559
GNAT2
C6orf106
TCF7L2
After heterogeneity correction
Adult BMI risk score weight
B
PWV estimated effect size
FPGT-TNNI3K
NT5C2
SMG6
RARB
EHBP1
TEMEM18
USP37
ASB4
Before heterogeneity correction
NT5C2
SMG6
USP37
ASB4
After heterogeneity correction
Adult BMI risk score weight
C
Age-AP estimated effect size
PMS2L11
HNF4G
EPB41L4B
TEMEM18
NAV1
Adult BMI risk score weight
D
PHV estimated effect size
RASA2
TLR4
FHIT
FUBP1
PRKD1
Adult BMI risk score weight

Fig. S10. Adult BMI GRS analysis of early growth traits. Scatter plots show on the x axis the effect size estimates (SD units) of the 97 adult BMI-associated SNP in GIANT consortium and in the y axis the corresponding effect size estimates (SD units) of the looked-up SNP of stage 1 meta-analysis GWAS on A) BMI at AP, B) PWV, C) Age at AP, D PWV. The effect size of the adult BMI increasing allele is plotted. Stage 1 meta-analysis GWAS SNPs with a P<0.05 are plotted with a solid circle and labeled with the nearest gene name. The 95% confidence intervals are denoted by grey skewers crossing the circle. The solid red line is the estimated effect of the genetic risk score on the early growth phenotype taking into account the uncertainty of the point estimates. The dashed red line is the 95% confidence interval of the predicted effect. GIANT BMI was inverse-normal rank transformed prior to association analysis.

Note S1. Literature search for epidemiological associations between early growth traits and childhood and adult traits

We conducted a literature review, aiming to locate epidemiological evidence for observational associations between the six early growth phenotypes under investigation in the present study and phenotypes related to body composition/anthropometry, cardio-metabolic disease, psychiatric disorders, neurological disease, lung function and educational attainment, in childhood or adulthood. An electronic search of Medline (Ovid) from inception until 7 October 2016 was carried out using the terms "peak weight velocity" OR "peak height velocity" OR "BMI peak" OR "adiposity peak" OR "BMI rebound" OR "adiposity rebound"; 685 results were returned. Abstracts were reviewed and papers were included if (a) they studied a sample judged likely to be representative of the general population, (b) they included exposures which were judged to be comparable to the phenotypes under investigation in the present study (peak weight velocity, peak height velocity, age and BMI at adiposity peak, age and BMI at adiposity rebound), (c) they included outcomes for which one or more significant genetic correlations were found in the LD-score regression analysis presented here (see table S14), (d) the full text of the paper was available in English and was accessible at time of searching and (e) no papers were found which studied the sample in question at a later time point. Overall 31 studies were included and are summarized in **table S13**. It should be noted that the aim was to gain an indication of the evidence available; this was not a systematic review and as such some relevant studies may have been missed.

Note S2. Cohort description (see also tables S1 and S2 for genotyping details and figs. S1 to S3)

Avon Longitudinal Study of Parents and Children [ALSPAC]: ALSPAC is a prospective study, which recruited 14,541 pregnant women resident in Avon, UK with expected dates of delivery 1st April 1991 to 31st December 1992. 14,541 is the initial number of pregnancies for which the mother enrolled in the ALSPAC study and had either returned at least one questionnaire or attended a "Children in Focus" clinic by 19/07/99. Of these *initial* pregnancies, there was a total of 14,676 fetuses, resulting in 14,062 live births and 13,988 children who were alive at 1 year of age. When the oldest children were approximately 7 years of age, an attempt was made to bolster the initial sample with eligible cases who had failed to join the study originally. As a result, when considering variables collected from the age of seven onwards (and potentially abstracted from obstetric notes) there are data available for more than the 14,541 pregnancies mentioned above. The number of **new pregnancies** not in the initial sample (known as Phase I enrolment) that are currently represented on the built files and reflecting enrolment status at the age of 18 is 706 (452 and 254 recruited during Phases II and III respectively), resulting in an additional 713 children being enrolled. The phases of enrolment are described in more detail in the cohort profile paper(*96*). The total sample size for analyses using any data collected after the age of seven is therefore 15,247 pregnancies, resulting in 15,458 fetuses. Of this **total sample** of 15,458 fetuses, 14,775 were **live births** and 14,701 were **alive at 1 year of age**. A 10% sample of the ALSPAC cohort, known as the **Children in Focus (CiF) group**, attended clinics at the University of Bristol at various time intervals between 4 to 61 months of age. The CiF group were chosen at random from the last 6 months of ALSPAC births (1432 families attended at least one clinic). Excluded were those mothers who had moved out of the area or were lost to follow-up, and those partaking in another study of infant development in Avon.
We used child's data from the first four clinical examinations at 6weeks, 9months, 18 months and pre - school [the heights and weights are reported to health visitors] and then clinic visits at ages 7,8,9,10,11, ~12 and ~14 yrs for follow-up analyses due sparse data in early childhood. Height was measured at the clinics to the last complete mm using the Harpenden Stadiometer and weight using the Tanita Body Fat Analyser [Model TBF 305] to the nearest 50g. Children [n=6704 in the present study] were genotyped using Illumina HumanHap550 quad genome-wide SNP genotyping platform by 23andMe subcontracting the Wellcome Trust Sanger Institute, Cambridge, UK and the Laboratory Corporation of America, Burlington, NC, USA. A common set of SNPs [present in both genotyping platforms] were extracted and the resulting raw genome-wide data was subjected to standard quality control methods. Individuals were excluded on the basis of having incorrect sex assignments; minimal [0.34] or excessive [0.36] heterozygosity; disproportionate levels of individual missingness [>3%] and evidence of cryptic relatedness [PI HAT >0.11]. The remaining individuals were assessed for evidence of population stratification by multidimensional scaling [MDS] analysis, using CEU, YRI, JPT and CHB individuals from the HapMap as reference ethnic groups. Those clustering with the CEU individuals were included in this study. SNPs which passed an exact test of Hardy-Weinberg equilibrium [P>5x10-7] were considered for analysis. The imputation was conducted with MACH 1.0 Markov Chain Haplotyping software17. Ethical approval for the study was obtained from the ALSPAC Law and Ethics Committee and the Local Research Ethics Committee. Please note that the study website contains details of all the data that is available through a fully searchable data dictionary, web page: http://www.bris.ac.uk/alspac/researchers/data-access/data-dictionary/.

Cambridge Baby Growth Study [CBGS]: This birth cohort study was recruited from mothers attending a single antenatal centre in Cambridge UK and comprises 1662 infants [born 2001-2009], representative of the South Cambridgeshire population. Mothers were approached and recruited-their first antenatal clinic appointment during early pregnancy by trained paediatric research nurses. Offspring were measured-0, 3, 12, 18 and 24 months by Paediatric research nurses. The study was approved by the local Cambridge research ethics committee and all mothers gave informed written consent. The current data are based on 557 infants in whom DNA samples were available from cord blood samples collected-birth. Individual SNP genotyping for replication was performed by TaqMan [Applied Biosystems]-the MRC Epidemiology Unit, Cambridge UK. For all SNPs, call rates were >99% and Hardy Weinberg Equilibrium P-values >0.05.

Children's Hospital of Philadelphia [CHOP]: All subjects were consecutively recruited from the Greater Philadelphia area from 2006 to 2010-the Children's Hospital of Philadelphia. Our study cohort consisted of

1,445 obese children and 2,802 lean children of European ancestry. In the present study 233 with growth and genetic data were included in replication. All of these participants had their blood drawn in to an 8ml EDTA blood collection tube and were subsequently DNA extracted for genotyping. All subjects were biologically unrelated and were aged between 2 and 18 years old. Height and weight measurements were collected from medical records. The study was approved by the Institutional Review Board of the Children's Hospital of Philadelphia. Parental informed consent was given for each study participant for both the blood collection and subsequent genotyping. Self-reported ethnicity was confirmed by multidimensional scaling methodologies. We performed high throughput genome-wide SNP genotyping, using the Illumina Infinium™ II HumanHap550 BeadChip technology [Illumina, San Diego],-the Center for Applied Genomics-CHOP.

COpenhagen Study on Asthma in Children [COPSAC]: The COPSAC birth cohort study is a prospective clinical study of a birth cohort of 411 infants born to mothers with a history of asthma. The newborns were enrolled-the Age of 1 month with regular follow-ups. The families attended the clinical research unit, not the family practitioner, for diagnosis and treatment of any respiratory or skin-related symptoms. Participants were assessed-the COPSAC clinical research unit-six monthly intervals; additional visits were arranged immediately upon the onset of symptoms. All growth parameters including height and weight were measured and obtained by the COPSAC clinicians and family, disease and developmental history was obtained using structured questions. High throughput genome-wide SNP genotyping was performed using the Illumina Infinium™ II HumanHap550 v1, v3 or quad BeadChip platform [Illumina, San Diego],-the Children's Hospital of Philadelphia's Center for Applied Genomics. In the replication we had 319 subjects with genetic and growth data. The study has been approved by the Ethics Committee for CopenhAgen [KF 01-289/96] and The Danish Data Protection Agency [2008-41-1754] and informed consent was obtained from both parents.

Danish National Birth Cohort [DNBC]: The Danish National Birth Cohort [DNBC] is a collection of data on 92,274 pregnant women recruited between 1996 and 2002. The children were followed through childhood and adolescence. The BMI data refer to the fourth interview when the child was 18 months. Mothers were asked to report height and weight from the child exams-Age 5 and 13 months and the record closest to Age 9 months was used in the replication analysis. Genotype data were available from a genome-wide association study on preterm birth performed with the Illumina 660 Quad chip, and genotypes for all SNPs from the HapMap phase 2 CEU reference set were imputed with MACH 1.0. The present analyses included 861 children for replication [447 boys, 414 girls]. Written informed consent was obtained-recruitment from all participating mothers on behalf of themselves and their children. The DNBC study protocol was approved by the Danish Scientific Ethical Committee and the Danish Data Protection Agency.

Étude des Déterminants pré et postnatals du développement et de la santé de l'ENfant [EDEN]: The EDEN study [Study of pre- and post-natal determinants of children's growth and development] is an on-going mother-child cohort in two centres, Nancy and Poitiers [France], with follow-up of the child until their fifth birthday. This study enrolled 2002 women during pregnancy and has been described previously in detail(*97*). The study was approved by the ethics committee [CCPPRB] of Kremlin Bicêtre [France] and by the Data Protection Authority "Comission Nationale de l'Informatique et des Libertés" [CNIL]. DNA was extracted from cord blood samples collected in 1367 of the 1907 births. Individual SNP genotyping was performed by TaqMan [Applied Biosystems]-the MRC Epidemiology Unit, Cambridge UK. For all SNPs, call rates were >99% and Hardy Weinberg Equilibrium P-values >0.05. Children had clinical examinations performed by the same midwife research assistants-birth and-one year, and 1127 children were included in replication. Age of one year, research midwives measured the weight of the mother alone and then holding their infant wearing light clothes using a Teraillon SL-351; infant weight was obtained by subtraction. A somatometer was used to measure infant length with a precision of 5 mm [NM Medical, Asnières, France]. Informed written consents

were obtained from the parents-enrollment for themselves and after delivery for the newborn. Please note that the study website contains the study contacts and further details, web page: http://eden.vjf.inserm.fr

The Exeter Family Study of Childhood Health [EFSOCH]: The Exeter Family Study of Childhood Health is a prospective study, set up to test the fetal insulin hypothesis, and to identify genetic polymorphisms that play a role in determining birth weight and early postnatal growth. We recruited 1017 families from a postcode-defined area in central Exeter. The parents gave informed consent for themselves and their children to participate in the study, and ethical approval was obtained from the local Institutional Review Board. Specific inclusion criteria were established to obtain a homogeneous, non-diabetic, UK Caucasian cohort. Detailed anthropometric measurements were taken from both parents-28 weeks' gestation and from their children-birth, 12 weeks, 1 year and 2 years of age. Parental and offspring DNA were extracted to allow molecular genetic analysis of candidate genes implicated in fetal growth. This comprehensive data set is available on a disease-free cohort, and detailed preparatory work has ensured high levels of quality control. Genotyping of the rs9436303 and rs10515235 polymorphisms was performed by LGC Genomics Ltd, Hoddesdon, Herts, UK [formerly Kbioscience; www.kbioscience.co.uk], using their own system of fluorescence-based competitive allele-specific PCR [KASPar]. The genotyping call rates were 92.0% and 95.1%, respectively. There was no evidence of deviation from Hardy-Weinberg equilibrium [P > 0.3]. The Exeter Family Study of Childhood Health (EFSOCH) was supported by South West NHS Research and Development, Exeter NHS Research and Development, the Darlington Trust and the Peninsula National Institute of Health Research (NIHR) Clinical Research Facility at the University of Exeter. The views expressed in this paper are those of the authors and not necessarily those of NIHR, the NHS or the Department of Health.

The Generation R Study: The Generation R Study is a population-based prospective cohort study from fetal life until young adulthood. All children were born between April 2002 and January 2006. This study is designed to identify early environmental and genetic determinants of growth, development and health from fetal life until young adulthood and has been described previously in detail(*98, 99*). Detailed measurements were performed using ultrasound, imaging techniques and physical examinations and biological samples were collected. The analysis was restricted to Caucasian individuals with genome-wide data and sufficient growth measurements available for growth modeling [n=2088]. Length was measured to the nearest millimeter and weight was recorded by well-trained staff in community health centers using standardized procedures. Visits to the community health centers were based on the national routine health care program. Sex- and age-adjusted standard deviation scores [SDS] were constructed using Growth Analyser 3.0 [http://www.growthanalyser.org; Dutch Growth Research Foundation, Rotterdam, the Netherlands]. The reference curve for body mass index in the Netherlands, 1997 was used. Cord blood for DNA isolation was available in 59% of all live-born participating children. Sex-mismatch rate between genome-based sex and midwive-record based sex was low [<0.5%], indicating that possible contamination of maternal DNA was extremely low. Missing cord blood samples were mainly due to logistical constraints-the delivery. Individual genotype data were extracted from the genome-wide Illumina 610 Quad Array51. The study has been approved by the Medical Ethics Committee of the Erasmus Medical Center, Rotterdam. Written informed consent was obtained from all participants or their parent[s].

Helsinki Birth Cohort Study [HBCS]: The Helsinki Birth Cohort Study [HBCS] includes individuals born at Helsinki University Central Hospital between the years 1934 and 1944. Birth records were collected on 4,630 men and 4,130 women born in this hospital and living in Finland in 1971. Serial measurements of height and weight until age 12 were extracted from child welfare clinic and school health care records, with an average of 10 measurements between birth and 2 years, and 8 measurements between 2 and 11 years of age. Between 2001 and 2004, when the individuals were between 59 and 70 years old, a representative subset of 928 males and 1075 females returned for clinical examinations, when adult stature was measured and blood was taken for DNA extraction. Genotyping was performed on the Illumina 670 Quad chip and calling was done with the

Illuminus algorithm at Sanger Centre on a total of 1566 samples and 1555 of them were included in discovery stage of the present study. Pre-imputation quality control filters included: call rate 95%, MAF > 0.01, HWE 1x10-6. Closely related individuals were screened by examining pair-wise IBD estimates. >0.2 IBD-sharing was used as cut-off. Population stratification was examined using multidimensional scaling analyses, but there were no outliers. Imputation was performed with MACH, and the analysis software was MACH2QTL. Post-imputation quality control filters were R2HAT >0.3 and MAF >0.01. HBCS has been approved by local ethics committees.

Lifestyle – Immune System – Allergy Study [LISA]: In the population-based prospective birth cohort study "Influences of Lifestyle-related Factors on the Immune System and the Development of Allergies in Childhood" [LISAplus] parents of neonates admitted to maternity hospitals in Munich, Leipzig, Wesel, and Bad Honnef, Germany were contacted. In total, 3097 healthy, term [gestational age over 37 weeks] neonates with a birth weight over 2500g were recruited between December 1997 and January 1999. Additionally, a total of 5991 mothers and their new-borns were recruited into the "German Infant Study on the Influence of Nutrition Intervention PLUS Environmental and Genetic Influences on Allergy Development" [GINIplus] between September 1995 and June 1998 in Munich and Wesel. Weight and height were measured with high time-resolute by the family physician during the first two years of life and obtained via medical records. DNA was collected at age 6 & 10 years. In the discovery analysis up to 390 children from the LISAplus study Munich were included. DNA was analysed using the Affymetrix Human SNP Array 5.0 for each individual. After quality control [MAF>1%, HWE>0.01, call rate per SNP and person >95%] genome-wide data was called using BRLMM-P algorithm and imputed in IMPUTE. For replication, an independent sample of up to 619 children from the LISAplus and GINIplus study Munich was analysed. DNA was analysed using the Affymetrix Human SNP Array 5.0 or 6.0 for each individual. Genotypes were called using BRLMM-P algorithm [5.0], respectively BIRDSEED V2 algorithm [6.0], imputed in IMPUTE2. For both studies, approval by the local Ethics Committees [Bavarian Board of Physicians, University of Leipzig, and Board of Physicians of North-Rhine-Westphalia] and written informed consent from participant's parents were obtained.

Northern Finland Birth Cohort Study 1966 [NFBC1966]: The NFBC1966 study [http://kelo.oulu.fi/NFBC/] includes 12,058 live born individuals, of European descent, with expected dates of birth during 1966 in the provinces of Oulu and Lapland, in Finland. Cohort has been followed up since early pregnancy until adult age. Prospective growth measurements [>20 from birth until the age of 16 years] were obtained from communal child health clinics(*38*). All those living in northern Finland or in the capital area were invited to a clinical examination and blood sampling-age 31 years(*100*). DNA was extracted from 5753 individuals and Illumina's HumanCNV370-Duo DNA Analysis BeadChip was used to obtain genome-wide genetic data. It contains an informative set of tag SNPs derived from the HapMap European-derived [CEU] sample(*101*). Imputation was performed on 328,007 SNPs using IMPUTE software version 0.3.1 58, applying information threshold of >0.4 and MAF threshold of >1%. GWAS and growth data for current analyses were available for 3459 children. The University of Oulu Ethics Committee and the Ethical Committee of Northern Ostrobothnia Hospital District have approved the study. Participants provided written informed consent.

Northern Finland Birth Cohort Study 1986 [NFBC1986]: The NFBC1986 study includes 9432 live-born individuals with expected dates of birth between July 1st 1985 and June 30th 1986 in the provinces of Oulu and Lapland, in Finland. Cohort has been followed up since early pregnancy until adolescence. Prospective growth measurements [>20 from birth until the age of 16 years] were obtained from communal child health clinics(*38*). Blood samples were collected as part of the 16-year follow-up and DNA was extracted for 6266 individuals. For 4020 children both growth and genetic data were available. The DNA samples were processed-Imperial College London, UK and custom genotyping was performed-LGC Genomics Ltd, Hoddesdon, Herts, UK [formerly Kbioscience]. The University of Oulu Ethics Committee and the Ethical Committee of Northern Ostrobothnia Hospital District have approved the study. Participants provided written informed consent.

The Western Australian Pregnancy Cohort [Raine] Study: The Raine Study was started as a randomized controlled trial to evaluate the effects of repeated ultrasound in pregnant women in Perth, Western Australia. In total, 2,900 pregnant women were recruited between 1989 and 1991 prior to 18-weeks gestation-the King Edward Memorial Hospital [Perth, Western Australia]. Women were randomized to repeated ultrasound measurements-18, 24, 28, 34 and 38 weeks gestation or to a single ultrasound assessment-18-weeks. Children have been assessed for growth [measures-visits], development and health at average ages of 1, 2, 3, 5, 8, 10, 14 and 17. DNA was collected-the year 14 and 17 follow-ups. The study was conducted with appropriate institutional ethics approval, and written informed consent was obtained from mothers-all follow-ups and participants-the year 17 follow-up. DNA was collected using standardized procedures from 74% of all adolescents who attended the 14 year follow-up on and a further 5%-the 17 year follow-up measurements. Genotype data was extracted from the genome-wide Illumina 660 Quad Array for each individual. Analysis(*102, 103*) was restricted to individuals with genome-wide data and BMI [n=1037].

INfancia y Medio Ambiente [Environment and Childhood] Project [INMA]: Population-based birth cohorts were established as part of the INMA – INfancia y Medio Ambiente [Environment and Childhood] Project in several regions of Spain following a common protocol. The present analysis uses the INMA cohorts of Valencia and Sabadell [Catalonia] established between 2003 and 2006 [n=396] for replication. The INMA project aims to study the associations between pre- and postnatal environmental exposures and growth, health, and development from early fetal life until adolescence. Pregnant women were enrolled during the 1st trimester of pregnancy-public primary health care centers or public hospitals. Detailed measurements were performed using ultrasound and physical examinations and biological samples. Informed consent was obtained from all participants and the study was approved by the Hospital Ethics Committees in each participating region. Cord blood for DNA isolation was available for 909/1409 [64.5%] of all live-born participating children and 752 samples of these were selected for the genome-wide genotyping using the HumanOmni1-Quad Beadchip [Illumina]. Sex-mismatch rate between genome-based sex and midwife-record based sex was 0, indicating that possible contamination of maternal DNA was extremely low.

The Physical Activity and Nutrition in Children [PANIC] Study. The Physical Activity and Nutrition in Children [PANIC] Study is a long-term controlled exercise and diet intervention study in a population sample of 512 primary school children conducted in the University of Eastern Finland. Baseline examinations- at age 6-8 years were performed in 2007-2009. Half of children were allocated into the exercise and diet intervention group and another half into the control group. The 2-year follow-up examinations- at age of 8-10 years [participation rate 86%] were done in 2009-2011. Overweight, obesity, body composition, fetal and early childhood growth and development were assessed comprehensively-baseline and 2-year follow-up. DNA was collected-the baseline and 2-year follow-up and analyzed using the Metabochip array. In total 433 children were included into the replication stage of the present study [genotypes retrieved from Metabochip Array]. The PANIC Study protocol was approved by the Research Ethics Committee of the Hospital District of Northern Savo in 2006. Both children and their parents gave their written informed consent.

The Southampton Women's Survey [SWS]: Between 1998 and 2002 all general practitioners in Southampton were asked to help us recruit their female patients aged 20-34 years to the study. Of those women contacted about the study, 12 583 [75%] agreed and were interviewed by a research nurse. All interviews were conducted between April 1998 and October 2002. Women who subsequently become pregnant were recruited into the pregnancy phase of the SWS. They visited the SWS Ultrasound Unit-the Princess Anne Hospital in Southampton-11, 19 and 34 weeks gestation where fetal and placental size and blood flows were measured using ultrasound. -11 and 34 weeks, the women were interviewed by research nurses, and anthropometric measurements of the baby were made within 48 hours of birth. By the end of 2007, when the pregnancy follow-up stopped, 3159 babies had been born to SWS women. Cord blood samples were obtained-birth from which DNA has been extracted. The children were followed up-home-6

months, 1, 2, and 3 years of age. Thereafter a subsample was seen-4 years and further subsamples-6, 8 and 10-12 years are currently being assessed. -all visits body composition is assessed by anthropometry and/or DXA. Buccal swabs are being taken-the 6, 8 and 10-12-year visits from which further DNA is being extracted. The custom genotyping was performed-LGC Genomics Ltd, Hoddesdon, Herts, UK [formerly Kbioscience]. The Southampton Women's Survey has received approval for all stages of the study from Southampton and South West Hampshire Local Research Ethics Committee. Follow-up of the children and sample collection/analysis was carried out under Institutional Review Board approval [Southampton and SW Hampshire Research Ethics Committee] with written informed consent. Investigations were conducted according to the principles expressed in the Declaration of Helsinki.

UK Biobank: The UK Biobank recruited over 500 000 adults aged 37–73 years in 2006–10 from across the UK. Participants provided samples and a range of information via questionnaires, physical measures, sample assays, accelerometry, multimodal imaging, genome-wide genotyping and longitudinal follow-up for a wide range of health-related outcomes and is described in detailed elsewhere(*104*).

Note S3. Funding and acknowledgments by the study

Avon Longitudinal Study of Parents And Children [ALSPAC]: We are extremely grateful to all the families who took part in this study, the midwives for their help in recruiting them, and the whole ALSPAC team, which includes interviewers, computer and laboratory technicians, clerical workers, research scientists, volunteers, managers, receptionists and nurses. GWAS data was generated by Sample Logistics and Genotyping Facilities at Wellcome Sanger Institute and LabCorp (Laboratory Corportation of America) using support from 23andMe. D. A. Lawlor's contribution to this paper is funded by European Research Council under the European Union’s Seventh Framework Programme (FP7/2007-2013) ERC grant agreement 669545. Debbie A. Lawlor, Nicholas J. Timpson, Susan M. Ring and George Davey Smith work in a Unit that receives funding from the University of Bristol and UK Medical Research Council (MC_UU_1201/1 and MC_UU_1201/5). Debbie .A. Lawlor is a UK National Institute of Health Research Senior Investigator (NF-SI-0166-10196). The Wellcome Trust (Grant ref: 102215/2/13/2) and the University of Bristol provide core support for ALSPAC.

Cambridge Baby Growth Study [CBGS]: We are grateful to the children and the parents who participated in this study, and the staff and facilities-the Wellcome Trust Clinical Research Facility [Addenbrooke’s Clinical Research Centre], Cambridge for assistance in infant assessments. We thank the CBGS co-investigators Prof Ieuan Hughes, Prof David Dunger and Dr Carlo Acerini, and Paediatric research nurses Suzanne Smith, Anne-Marie Wardell and Karen Forbes-the Department of Paediatrics, University of Cambridge. The CBGS was supported by the European Union, the World Cancer Research Foundation International, the Medical Research Council, the Newlife Foundation and the NIHR Cambridge Biomedical Research Centre.

Children’s Hospital Of Philadelphia [CHOP]: The authors thank the network of primary care clinicians and the patients and families for their contribution to this project and to clinical research facilitated by the Pediatric Research Consortium [PeRC]-The Children’s Hospital of Philadelphia. R. Chiavacci, E. Dabaghyan, A. [Hope] Thomas, K. Harden, A. Hill, C. Johnson-Honesty, C. Drummond, S. Harrison, F. Salley, C. Gibbons, K. Lilliston, C. Kim, E. Frackelton, F. Mentch, G. Otieno, K. Thomas, C. Hou, K. Thomas and M.L. Garris provided expert assistance with genotyping and/or data collection and management. The authors would also like to thank S. Kristinsson, L.A. Hermannsson and A. Krisbjörnsson of Raförninn ehf for extensive software design and contributions. This research was financially supported by an Institute Development Award from the Children’s Hospital of Philadelphia, a Research Development Award from the Cotswold Foundation, the Daniel B. Burke Endowed Chair for Diabetes Research and NIH grant R01 HD056465.

Copenhagen Study on Asthma in Children [COPSAC]: We gratefully express our gratitude to the children and families of the COPSAC2000 cohort study for all their support and commitment. We acknowledge and appreciate the unique efforts of the COPSAC research team. COPSAC is funded by private and public research funds listed on www.copsac.com. The Lundbeck Foundation; The Danish Strategic Research Council; the Pharmacy Foundation of 1991; Augustinus Foundation; the Danish Medical Research Council and The Danish Pediatric Asthma Centre provided the core support for COPSAC research center. No pharmaceutical company was involved in the study. The funding agencies did not have any role in design and conduct of the study; collection, management, and interpretation of the data; or preparation, review, or approval of the manuscript.

Danish National Birth Cohort [DNBC]: Funding support for the Danish National Birth Cohort [DNBC] was provided by the Danish National Research Foundation, the Danish Pharmacists’ Fund, the Egmont Foundation, the March of Dimes Birth Defects Foundation, the Augustinus Foundation and the Health Fund of the Danish Health Insurance Societies. The genotype data were derived from a GWAS of preterm birth in the Gene Environment Association Studies [GENEVA] consortium funded by the NIH Genes, Environment and Health Initiative [GEI, U01HG004423]. Genotyping was performed-Johns Hopkins University Center for Inherited Disease Research, with support from the NIH GEI [U01HG004438]. The study protocol was approved

by the Danish Scientific Ethical Committee and the Danish Data Protection Agency for all Danish subjects. The analysis was supported in part by grants from the Lundbeck Foundation [R19-A2059] and the Danish Medical Research Council [09-065592].

Étude des Déterminants pré et postnatals du dévelopement et de la santé de l'ENfant [EDEN]: The EDEN study was supported by grants from Fondation pour la Recherche Médicale, French Ministry of Research, Institut National de la Santé et de la Recherche Médicale [INSERM], French Ministry of Health, French Agency for Occupational and Environmental Health Safety, French National Institute for Population Health Surveillance, University of Paris–Sud, French National Institute for Health Education, Nestlé, Mutuelle Générale de l'Education Nationale, French Speaking Association for the Study of Diabetes and Metabolism, and French Research Agency.

Exeter Family Study Of Childhood Health [EFSOCH]: "The Exeter Family Study of Childhood Health (EFSOCH) was supported by South West NHS Research and Development, Exeter NHS Research and Development, the Darlington Trust and the Peninsula National Institute of Health Research (NIHR) Clinical Research Facility at the University of Exeter. The views expressed in this paper are those of the authors and not necessarily those of NIHR, the NHS or the Department of Health. Rachel M. Freathy is a Sir Henry Dale Fellow (Wellcome Trust and Royal Society grant: 104150/Z/14/Z). Timothy M. Frayling is supported by the European Research Council grant: 323195 SZ-24550371- GLUCOSEGENES-FP7-IDEAS-ERC. Andrew T. Hattersley is a Wellcome Trust Senior Investigator (098395/Z/12/Z) and an NIHR Senior Investigator (NF-SI-0611-10219).

The Generation R Study [GenR]: The Generation R Study [GenR]:The Generation R Study is conducted by the Erasmus Medical Center in close collaboration with the School of Law and Faculty of Social Sciences of the Erasmus University Rotterdam, the Municipal Health Service Rotterdam area, Rotterdam, the Rotterdam Homecare Foundation, Rotterdam and the Stichting Trombosedienst & Artsenlaboratorium Rijnmond [STAR-MDC], Rotterdam. We gratefully acknowledge the contribution of children and parents, general practitioners, hospitals, midwives and pharmacies in Rotterdam. The generation and management of GWAS genotype data for the Generation R Study were done-the Genetic Laboratory of the Department of Internal Medicine, Erasmus MC, The Netherlands. We would like to thank Karol Estrada, Dr. Tobias A. Knoch, Anis Abuseiris, Luc V. de Zeeuw, and Rob de Graaf, for their help in creating GRIMP, BigGRID, MediGRID, and Services@MediGRID/D-Grid, [funded by the German Bundesministerium fuer Forschung und Technology; grants 01 AK 803 A-H, 01 IG 07015 G] for access to their grid computing resources. We thank Mila Jhamai, Manoushka Ganesh, Pascal Arp, Marijn Verkerk, Lizbeth Herrera and Marjolein Peters for their help in creating, managing and QC of the GWAS database. Also, we thank Karol Estrada and Carolina Medina-Gomez for their support in creation and analysis of imputed data.

The general design of Generation R Study is made possible by financial support from the Erasmus Medical Center, Rotterdam, the Erasmus University Rotterdam, the Netherlands Organization for Health Research and Development (ZonMw), the Netherlands Organisation for Scientific Research (NWO), the Ministry of Health, Welfare and Sport and the Ministry of Youth and Families. Additionally, we received funding from the Netherlands Organization for Health Research and Development (VIDI 016. 136. 361), the European Union's Horizon 2020 research and innovation programme under grant agreement No 633595 (DynaHEALTH), the European Union's Horizon 2020 research and innovation programme under grant agreement No 733206(LIFECYCLE), , European Research Council (ERC Consolidator Grant, ERC-2014-CoG-648916).

Helsinki Birth Cohort Study [HBCS]: We thank Professor David Barker, Professor Clive Osmond, Associate professors Eero Kajantie and Tom Forsen. Major financial support was received from the Academy of Finland [project grants 209072, 129255 grant] and British Heart Foundation and the Academy of Finland [grants

134839 and 129287]. Additionally, we received funding from the European Union's Horizon 2020 research and innovation programme under grant agreement No 633595 (DynaHEALTH), the European Union's Horizon 2020 research and innovation programme under grant agreement No 733206(LIFECYCLE). The DNA extraction, sample quality control, biobank up-keep and aliquotting was performed-the National Public Health Institute, Helsinki, Finland.

INfancia y Medio Ambiente [Environment and Childhood] Project [INMA]: This study was funded by grants from Instituto de Salud Carlos III [CB06/02/0041, FIS PI041436, PI081151, PI041705, and PS09/00432, FIS-FEDER 03/1615, 04/1509, 04/1112, 04/1931, 05/1079, 05/1052, 06/1213, 07/0314, and 09/02647], Spanish Ministry of Science and Innovation [SAF2008-00357], European Commission [ENGAGE project and grant agreement HEALTH-F4-2007-201413], Fundació La Marató de TV3, Generalitat de Catalunya-CIRIT 1999SGR 00241, Conselleria de Sanitat Generalitat Valenciana, and Fundación Roger Torné. The authors are grateful to Silvia Fochs, Anna Sànchez, Maribel López, Nuria Pey, Muriel Ferrer, Amparo Quiles, Sandra Pérez, Gemma León, Elena Romero, and Amparo Cases for their assistance in contacting the families and administering the questionnaires. The authors would particularly like to thank all the participants for their generous collaboration. A full roster of the INMA Project Investigators can be found-http://www.proyectoinma.org/presentacion-inma/listado-investigadores/en listado-investigadores.html.

Lifestyle – Immune System – Allergy [LISA] Study Munich: The LISAplus study was mainly supported by grants from the Federal Ministry for Education, Science, Research and Technology and in addition from Helmholtz Zentrum Munich [former GSF], Helmholtz Centre for Environmental Research - UFZ, Leipzig, Research Institute-Marien-Hospital Wesel, Pediatric Practice, Bad Honnef for the first 2 years. The 4 year, 6 year, and 10 year follow-up examinations of the LISAplus study were covered from the respective budgets of the involved partners [Helmholtz Zentrum Munich [former GSF], Helmholtz Centre for Environmental Research - UFZ, Leipzig, Research Institute-Marien-Hospital Wesel, Pediatric Practice, Bad Honnef, IUF – Leibniz-Research Institute for Environmental Medicine-the University of Düsseldorf] and in addition by a grant from the Federal Ministry for Environment [IUF Düsseldorf, FKZ 20462296]. The GINIplus study was mainly supported for the first 3 years of the Federal Ministry for Education, Science, Research and Technology [interventional arm] and Helmholtz Zentrum Munich [former GSF] [observational arm]. The 4 year, 6 year, and 10 year follow-up examinations of the GINIplus study were covered from the respective budgets of the 5 study centres [Helmholtz Zentrum Munich [former GSF], Research Institute-Marien-Hospital Wesel, LMU Munich, TU Munich and from 6 years onwards also from IUF - Leibniz Research-Institute for Environmental Medicine-the University of Düsseldorf] and a grant from the Federal Ministry for Environment [IUF Düsseldorf, FKZ 20462296].

Northern Finland Birth Cohort 1966 [NFBC1966] and 1985-86 [NFBC1986]: We thank late Professor Paula Rantakallio [launch of NFBC1966 and 1986], Ms Outi Tornwall and Ms Minttu Jussila [DNA biobanking]. Financial support was received from the Academy of Finland [project grants 104781, 120315, 129269, 1114194, Center of Excellence in Complex Disease Genetics and SALVE], University Hospital Oulu, Biocenter, University of Oulu, Finland [75617], the European Commission [EURO-BLCS, Framework 5 award QLG1-CT-2000-01643], NHLBI grant 5R01HL087679-02 through the STAMPEED program [1RL1MH083268-01], NIH/NIMH [5R01MH63706:02], the Medical Research Council, UK [G0500539, G0600705, PrevMetSyn/SALVE] and the Wellcome Trust [project grant GR069224], UK, ENGAGE project and grant agreement HEALTH-F4-2007-201413, and the EU Framework Programme 7 small-scale focused research collaborative project EurHEALTHAgeing 277849. The DNA extractions, sample quality controls, biobank up-keeping and aliquotting were performed in the National Public Health Institute, Biomedicum Helsinki, Finland and supported financially by the Academy of Finland and Biocentrum Helsinki.

The Western Australian Pregnancy Cohort [Raine] Study: The authors are grateful to the Raine Study participants and their families and to the Raine Study team for cohort coordination and data collection. The

authors gratefully acknowledge the NHMRC for their long term contribution to funding the study over the last 29 years and also the following Institutions for providing funding for core management of the Raine Study: The University of Western Australia, Raine Medical Research Foundation, The Telethon Kids Institute, Women's and Infants' Research Foundation, Curtin University, Edith Cowan University, Murdoch University, The University of Notre Dame Australia. The authors gratefully acknowledge the assistance of the Western Australian DNA Bank [National Health and Medical Research Council of Australia National Enabling Facility]. The authors also acknowledge the support of the National Health and Medical Research Council of Australia [Grant ID's 403981, 003209 and 572613] and the Canadian Institutes of Health Research [Grant ID MOP-82893]. N.M.Warrington is supported by a National Health and Medical Research Council Early Career Fellowship (APP1104818).

The Physical Activity and Nutrition in Children [PANIC] Study: The authors are grateful to all voluntary subjects and their families and the research team. The PANIC Study was financially supported by grants from Ministry of Social Affairs and Health of Finland, Ministry of Education and Culture of Finland, Finnish Innovation Fund Sitra, Social Insurance Institution of Finland, Finnish Cultural Foundation, Juho Vainio Foundation, Foundation for Paediatric Research, Paavo Nurmi Foundation, Paulo Foundation, The Diabetes Research Foundation and Kuopio University Hospital.

Southampton Women's Survey [SWS]: We thank the women of Southampton and their children who gave their time to take part in the study, the general practitioners and midwives in Southampton for their support and the Southampton Women's Survey staff for all their work in recruiting and interviewing the participants, and processing the data and samples. Funding for the components of the Southampton Women's Survey contributing to this research came from the Medical Research Council, the University of Southampton, the Dunhill Medical Trust and the National Institute for Health Research Southampton Biomedical Research Centre.

UK Biobank: UK Biobank has received funding from the UK Medical Research Council, Wellcome Trust, Department of Health, British Heart Foundation, Diabetes UK, Northwest Regional Development Agency, Scottish Government, and Welsh Assembly Government. As described in the manuscript, the MRC and Wellcome Trust played a key role in the decision to establish UK Biobank, a large, population-based, prospective, open access resource that would allow detailed investigations of the genetic and environmental determinants of the diseases of middle and old age. The MRC, Wellcome Trust, Department of Health, and Scottish Chief Scientist Office each have a representative on the UK Biobank Board. The MRC and Wellcome Trust fund the independent Ethics and Governance Council.
